# Author Correction: Dynamic changes of muscle insulin sensitivity after metabolic surgery

**DOI:** 10.1038/s41467-022-29350-0

**Published:** 2022-06-10

**Authors:** Sofiya Gancheva, Meriem Ouni, Tomas Jelenik, Chrysi Koliaki, Julia Szendroedi, Frederico G. S. Toledo, Daniel F. Markgraf, Dominik H. Pesta, Lucia Mastrototaro, Elisabetta De Filippo, Christian Herder, Markus Jähnert, Jürgen Weiss, Klaus Strassburger, Matthias Schlensak, Annette Schürmann, Michael Roden

**Affiliations:** 1 Division of Endocrinology and Diabetology, Medical Faculty, Heinrich-Heine University, Düsseldorf, Germany; 2 Institute for Clinical Diabetology, German Diabetes Center, Leibniz Center for Diabetes Research, Heinrich Heine University, Düsseldorf, Germany; 3 German Center for Diabetes Research (DZD e.V.), Neuherberg, Germany; 4 German Institute of Human Nutrition, Department of Experimental Diabetology, German Institute of Human Nutrition, Potsdam-Rehbruecke, Germany; 5 Laikon University Hospital, Athens, Greece; 6 Division of Endocrinology and Metabolism, Department of Medicine, University of Pittsburgh School of Medicine, Pittsburgh, PA USA; 7 Institute for Clinical Biochemistry and Pathobiochemistry, German Diabetes Center, Leibniz Center for Diabetes Research, Heinrich Heine University, Düsseldorf, Germany; 8 Institute for Biometrics and Epidemiology, German Diabetes Center, Leibniz Center for Diabetes Research, Heinrich Heine University, Düsseldorf, Germany; 9 General Surgery Department, Schön Clinics, Düsseldorf, Germany; 10 Institute of Nutritional Sciences, University of Potsdam, Nuthetal, Germany

**Keywords:** DNA methylation, Obesity

Correction to: *Nature Communications* 10.1038/s41467-019-12081-0, published online 13 September 2019.

The original version of this article included errors in the Results and Supplementary information. During analysis of annotation of gene expression data, the skeletal muscle transcriptome data of the different microarrays used for the samples at different time points (before and after the surgery), was combined and normalized, but the command “merge” of the Software R was used incorrectly in this step. The incorrect R code used in the first submission was: “dataset1_2 <- merge(dataset1[, 1:49], dataset2[, 1:9]) dataset<-merge(dataset1_2,dataset3[,1:31],by=“TargetName”.

These two “merge” commands combined the expression values of all samples of three microarrays. The mistake was that the gene-annotation information-columns were not considered. The “merge” command has the argument “sort”, which by default is TRUE, leading to an alphabetical sorting of the “TargetName” column. This led to a wrong combining of the gene-annotation information-columns and the “TargetName” in a later step.

The data has now been reanalyzed, only columns with the expression data were used, and the command “inner_join” was applied, because this command always keeps the row-order. The correct code is: “dataset1_2 <- inner_join(dataset1[, 1:49], dataset2[, 1:9],by=“TargetName”) dataset<-inner_join(dataset1_2,dataset3,by=“TargetName”. The corrected paper has been reviewed by the original reviewers #1 and #2, and by a new reviewer. The conclusions are not affected. The following changes have been made in the text and figures:

The sentence that read “To examine whether the metabolic differences reflect altered gene transcription, we initially compared the muscle transcriptome between OB before surgery (OB 0 w) and CON and detected 1037 differentially expressed genes (Fig. 3a). Gene ontology (GO) analysis indicated that several genes are implicated in the biosynthesis of long-chain fatty acyl-CoA and fatty acids as well as reactive oxygen species (ROS) metabolism (Fig. 3b; Supplementary Table 3)”. now reads “To examine whether the metabolic differences reflect altered gene transcription, we initially compared the muscle transcriptome between OB before surgery (OB 0 w) and CON and detected 595 differentially expressed genes (Fig. 3a). Gene ontology (GO) analysis indicated that several genes are implicated in the regulation of apoptotic processes (GO:0042981), MAPK cascade (GO:0043410), inflammatory response (GO:0006954, GO:0050729), oxygen transport (GO:0015671), and others (Fig. 3b; Supplementary Table 3).

The sentence that read “Comparing the muscle transcriptome of OB over the time course after surgery identified 783 out of 1287 upregulated and 504 downregulated genes at 2 weeks (Fig. 3a). GO analysis showed significant enrichments in genes controlling catabolic processes, lipid metabolism, and phosphorylation (GO:0016310; Fig. 3c, Supplementary Table 4). Among all transcripts, 1072 were transiently altered at 2 weeks and then returned to baseline values (see also below), whereas 215 remained constantly changed (Fig. 3a). These transcripts comprise genes related to the adenylate cyclase-modulating G-protein coupled receptor signaling pathway, lipid metabolism and regulation of gene expression (Supplementary Fig. 5a, Supplementary Table 5)”. now reads “Comparing the muscle transcriptome of OB over the time course after surgery identified 937 out of 1528 upregulated and 591 downregulated genes at 2 weeks (Fig. 3a). GO analysis showed significant enrichments in genes controlling transcriptional regulation (GO:0000398, GO:0010501), small GTPase-mediated signal transduction (GO:0051056), and negative regulation of insulin receptor signaling (GO:0046627) (Fig. 3c; Supplementary Table 4). Among all transcripts, 1244 were transiently altered at 2 weeks and then returned to baseline values (see also below, Fig. 6b), whereas 203 remained constantly changed (Fig. 3a). These transcripts comprise genes related to regulation of gene expression (GO:0010467), calcium-mediated signaling (GO:0019722), and negative regulation of insulin receptor signaling (GO:0046627) (Supplementary Fig. 5a, Supplementary Table 5)”.

The sentence that read “The genes exhibiting strongest changes at 2 weeks are involved in mitochondrial function (MTUS1, TRMT6), fatty acid metabolism (SLC27A4), calcium signaling (ATP2C2), transcriptional regulation (ZNF329, GTF2E2), protein transport (RAB3D), and inflammatory processes (IL18RAP) (Supplementary Table 1)”. now reads “The genes exhibiting strongest changes at 2 weeks are involved in mitochondrial function (*HMGCS2*), lipid metabolism (*ANGPTL4*, *ABCA1*, and *ABCG1*), calcium signaling (*PCDH15*), protein folding (*DNAJC28*), and inflammatory processes (*CISH*) (Supplementary Table 1)”.

The sentence that read “This revealed 49 mitochondrial, 24 lipid metabolism and 36 calcium-related genes with significantly different expression levels (Fig. 3d)”. now reads “This revealed 70 mitochondrial, 23 lipid metabolism and 60 calcium-related genes with significantly different expression levels (Fig. 3d)”.

The sentences that read “At 12 weeks after surgery, 868 genes were significantly altered (604 up- and 264 downregulated), at 24 weeks 709 genes (563 up- and 146 downregulated) in comparison to their expression before surgery (Fig. 3a). The pathways affected at 12 weeks comprise—among others—glucose homeostasis (GO0042593) and negative regulation of phosphate activity (GO:0006814) (Supplementary Fig. 5b, Supplementary Table 6). At 24 weeks, genes related to positive regulation of GTPase activity (GO:0043547) and protein autophosphorylation (GO:0046777) as well as ion transmembrane transport (GO:0034765) are mostly higher expressed than before surgery (Supplementary Fig. 5c, Supplementary Table 7). At 52 weeks, 1535 transcripts were significantly altered (Fig. 3a; Supplementary Table 6), which comprise genes involved in calcium and sodium transport and interferon-γ signaling (Supplementary Fig. 5b) including upregulation of those contributing to transcription (BACH1), cytoskeletal and tubulin reorganization (CCDC87) (Supplementary Table 8)”. now read “At 12 weeks after surgery, 625 genes were significantly altered (341up- and 284 downregulated), at 24 weeks 756 genes (519 up- and 237 downregulated) in comparison to their expression before surgery (Fig. 3a). The pathways affected at 12 weeks comprise—among others—glycogen metabolic process (GO:0005977), translation (GO:0006413 and GO:0006364), and transmembrane transport (GO:0055085) (Supplementary Fig. 5b, Supplementary Table 6). At 24 weeks, genes related to transmembrane receptor protein tyrosine kinase signaling (GO:0007169), cytoskeleton organization (GO:0007010), and regulation of cell growth (GO:0001558) are mostly higher expressed than before surgery (Supplementary Table 7). At 52 weeks, 1449 transcripts were significantly altered (Fig. 3a, Supplementary Table 8), which comprise genes involved glycogen metabolic process (GO:0005977), protein polyubiquitination/destabilization (GO:0016567, GO:0031648), (Supplementary Table 8) and including upregulation of those contributing to cytoskeletal and tubulin reorganization (*MYH3*, *CCDC87*, and *ACTC1*) (Supplementary Table 8)”.

The sentences that read “This enrichment explains the small overlap between differentially methylated CpGs and differentially expressed genes, as only 1467 CpGs are located in/or close to regions of the 430 differentially expressed genes (Fig. 4a). Among differentially expressed genes involved in mitochondrial function, lipid metabolism and calcium signaling, we detected 17, 4, and 15, respectively, which were affected by DNA methylation at 52 weeks (Fig. 3d, highlighted in magenta)”. now read “This enrichment explains the relatively small overlap between differentially methylated CpGs and differentially expressed genes, as only 2956 CpGs are located in/or close to regions of the 921 differentially expressed genes (Fig. 4a). Among differentially expressed genes involved in mitochondrial function, lipid metabolism, and calcium signaling, we detected 13, 6 and 30, respectively, which were affected by DNA methylation at 52 weeks (Fig. 3d, highlighted in magenta)”.

The sentence that read “However, at 52 weeks, 1467 CpGs were identified in 430 differentially expressed genes (Fig. 4a). GO analysis indicated enrichment in genes linked to 12 biological pathways, including cAMP biosynthesis, lysosomal organization, and muscle cell differentiation (Fig. 4b; Supplementary Table 9). Among those, 230 contain >2 differentially methylated CpGs and levels of altered DNA methylation ranging from 5 to 10%”. now reads “However, at 52 weeks, 2956 CpGs were identified in 921 differentially expressed genes (Fig. 4a). GO analysis indicated enrichment in genes linked to several biological pathways, including protein phosphorylation, glycogen metabolic process (GO:0005977), protein localization to plasma membrane, and vesicle-mediated transport (Fig. 4b; Supplementary Table 9). Among those, 323 contain >2 differentially methylated CpGs and levels of altered DNA methylation are higher than 5%”.

The sentences “Examples are TBC1D1, encoding a Rab-GTPase activating protein, TBC1 domain family member 1, and ASPSCR1, encoding UBX domain-containing tether for GLUT4. Lower promoter methylation of TBC1D1 associated with higher expression (Fig. 5a), whereas hypomethylation of ASPSCR1 in the gene body related to lower expression at 52 weeks (Fig. 5b). Of note, both genes are involved in glucose transport via glucose transporter 4 (GLUT4)20,21. In addition, both NR4A1 (nuclear receptor subfamily 4 group A member 1) and ELOVL5 (ELOVL fatty acid elongase 5) exhibited hypermethylated promoters and hypomethylated gene bodies corresponding to lower expression (Fig. 5c, d). ELOVL5 is involved in the elongation of long-chain polyunsaturated fatty acids 22 and upregulated upon high-fat feeding 23, NR4A1 encodes a nuclear receptor and transcription factor regulating the expression of genes involved in glucose metabolism 24. In addition, the time course of the expression of the listed candidates was evaluated. TBC1D1 expression increased and ASPSCR1 decreased already at 2 weeks, whereas the changes of the expression of ELOVL5 and NR4A1 occurred only at 52 weeks (Supplementary Fig. 9)”. now read “Examples are *PTPRE*, a negative regulator of insulin receptor (IR) signaling in skeletal muscle; *PIK3R1*, a regulator of the PI3-kinase; *MLXIP*, involved in transcriptional activation of glycolytic target genes, and *ACACB*, a mitochondrial enzyme playing a role in fatty acid metabolism (Fig. 5a–d). A hypermethylation in the promoter region of *PTPRE* gene related to lower expression at 52 weeks (Fig. 5a). A lower DNA methylation in the promoter and a higher methylation in the gene body of *PIK3R1* is linked to its higher expression after the surgery (Fig. 5b), whereas a hypomethylation in the gene body of *MLXIP* and *ACACB* associated with their lower expression (Fig. 5c, d). In addition, the time course of the expression of the listed candidates was evaluated. *PTPRE* expression decreased already at 2 weeks, whereas the changes of the expression of *PI3KR1* occurred at 12 weeks and *ACACB* and *MLXIP* at 52 weeks (Supplementary Fig. 9)”.

The sentences that read “Table 2 lists the expression levels of several genes associated with changes in M-value (27), fasting glucose (231), HMW-adiponectin (61), and mitochondrial content (13). GO analysis of the 231 affected genes, which correlated to glucose concentrations, can be linked to regulation of DNA binding (GO:0043388), macromolecule catabolism (GO:0009057), exocytosis (GO:0006887), secretion (GO:0032940), and others (Supplementary Table 10). The correlation analysis of the 430 differentially expressed and methylated genes (at 52 weeks) revealed 43 genes associated with BMI, 189 with M-value, and 31 with HMW-adiponectin (Table 3). The correlations between methylation levels and M-value of the OB group for ASPSCR1 (R2 = 0.233; *p* = 0.0009, Pearson correlation) and ELOVL5 (R2 = 0.234; *p* = 0.0009, Pearson correlation) indicate that their interindividual epigenetic alterations are linked to the improvement in insulin sensitivity (Supplementary Fig. 10).” now read “In order to relate the differential expression and changes in DNA methylation to the primary metabolic phenotypes, e. g. body weight, insulin sensitivity, we calculated their correlation. Table 2 lists the expression levels of several genes associated with changes in M-value (27), fasting glucose (1219), HMW-adiponectin (73), and mitochondrial content (29). GO analysis of the 1219 affected genes, which correlated to glucose concentrations, can be linked to cytoskeleton organization (GO:0007010), calcium signaling (GO:0016338), and others (Supplementary Table 10). The correlation analysis of the 921 differentially expressed and methylated genes (at 52 weeks) revealed 177 genes associated with BMI, 443 with M-value, and 70 with HMW-adiponectin (Table 3). The correlations between methylation levels and M-value of the OB group for *PTPRE* (R2 = 0.286; *p* = 10−4, Pearson correlation) and *PIK3R1* (R2 = 0.312; *p* = 5.10−5, Pearson correlation) indicate that their interindividual epigenetic alterations are linked to the improvement in insulin sensitivity (Supplementary Fig. 10)”.

The sentence “As 1072 transcripts (encoded by 468 genes) were only transiently differentially expressed at 2 weeks (Fig. 3a) and returned to baseline expression levels, we tested whether epigenetic alterations were responsible for this effect”. now reads “As 1150 mRNAs (encoded by 1126 genes) were only transiently differentially expressed at 2 weeks (Fig. 3a) and returned to baseline expression levels, we tested whether epigenetic alterations were responsible for this effect”.

The sentence “Indeed, 93% (438) of these genes showed changes in DNA methylation at 52 weeks (Fig. 6a; Chi-square *p* < 10−255), including genes involved in mitochondrial function (*n* = 22), calcium signaling (*n* = 20), lipid metabolism (*n* = 10). Representative examples comprise FTO, an obesity-related gene encoding α-ketoglutarate dependent dioxygenase 25, and TOMM7, a translocase of outer mitochondrial membrane 7 involved in translocation of pre-proteins into mitochondria 26,27 (Fig. 6b; Supplementary Fig. 9)”. now reads “Indeed, 75% (849) of these genes showed changes in DNA methylation at 52 weeks (Fig. 6a; Chi-square *p* < 10^−255^), including genes involved in mitochondrial function (*n* = 12), calcium signaling (*n* = 11), lipid metabolism (*n* = 4). Representative examples comprise *HMGCS2* (hydroxymethylglutaryl-CoA synthase^20^, and *IMMP2L* a mitochondrial inner membrane protease subunit 2 involved in peptides translocation into mitochondria^21,22^ (Fig. 6b; Supplementary Fig. 9)”.

The sentence “Before surgery, as described previously^13,19^, particularly genes involved in lipid metabolism, such as *SCD5* (stearoyl-CoA desaturase 5), exhibited differential expression” now reads “Before surgery, as described previously^13,19^ particularly genes involved in lipid metabolism, such as *FFAR4* (free fatty acid receptor 4), exhibited differential expression”.

The sentence “Elevated SLC27A4 (fatty acid transporter 4) expression at 2 weeks likely reflects the higher FFA uptake 50. Also, genes related to mitochondria and calcium handling showed higher expression levels, such as ATP2C2, encoding a manganese-transporting calcium ATPase 51, and MCUR1, a key regulator of oxidative phosphorylation 52, encoding a mitochondrial calcium uniporter protein required for calcium”. now reads “Elevated *ABCD3* (ATP-binding cassette sub-family D member 3) or *NAPELD* (N-acyl-phosphatidylethanolamine-hydrolyzing phospholipase D) expression at 2 weeks likely reflects the higher FFA uptake^45,46^. Also, genes related to mitochondria and calcium handling showed higher expression levels, such as *SLC25A25* (calcium-binding mitochondrial carrier protein SCaMC-2) encoding a calcium-dependent mitochondrial solute carrier^47^”.

The sentences “Consequently, elevated MCUR1 expression can be linked to the evanescent stimulation of β-oxidation. Another set of transiently upregulated genes relates to inflammatory processes, such as IL18RAP, encoding the receptor accessory protein of the pro-inflammatory interleukin 18 (IL18)^54^. Interestingly, carriers of polymorphisms in the IL18RAP gene suffer from greater susceptibility to obesity^54^”. now read “Another set of transiently upregulated genes relates to small GTPase-mediated signaling, such as *ARHGAP24, RACGAP1, ITSN1* and to negative regulation of insulin signaling, such as *SOCS1, RPS6KB1*. The latest mediates TNF-alpha-induced insulin resistance by phosphorylating IRS1 at multiple serine residues, resulting in accelerated degradation of IRS1^49−51^”.

The sentence that read “Likewise, MTUS1, possibly contributing to protection against pro-inflammatory response of endothelial cells 55, may be involved in the anti-inflammatory defense at 2 weeks. In addition, MTUS1 has recently been shown to be involved in mitochondrial motility, fusion, and in the maintenance of mitochondrial morphology 56 and its reduction at 2 weeks may serve to support the evidence for alterations of mitochondrial content and function early after surgery.” has now been deleted.

The sentences “Nearly 1500 of the differentially methylated CpGs are associated with altered expression of the corresponding 430 genes, pointing towards the relevance of epigenetic mechanisms in response to weight loss. Metabolic surgery remodels DNA methylation of glucose and lipid metabolism-related genes, such as *TBC1D1*, contributing to GLUT4 translocation^20^, *ASPSCR1*, a tethering protein sequestering GLUT4-containing vesicles^21^ and *ELOVL5*, which is essential for fatty acid synthesis^22^ and known to be upregulated in human skeletal muscle upon overfeeding^23^”. now read “Nearly 3000 of the differentially methylated CpGs are associated with altered expression of the corresponding 921 genes, pointing towards the relevance of epigenetic mechanisms in response to weight loss. Metabolic surgery remodels DNA methylation of genes involved in insulin signaling, such *as PIK3R1* and *PTPRE* and *ACACB* (also designated *ACC2*), which is essential for fatty acid metabolism^59^. Mice lacking *Acc2* exhibit higher fatty acid oxidation rates in the soleus muscle than control mice^60^”.

The sentences “The present study identified close to 100 epigenetic changes associated with altered expression of important metabolic genes, likely contributing to improved insulin sensitivity and lipid metabolism. Pathway enrichment analysis of the 430 differentially expressed and methylated genes indicates that several genes are implicated in the regulation of muscle cell differentiation, intracellular signal transduction, and cAMP biosynthesis, reflecting improved skeletal muscle activity and metabolism^64^. “now reads “The present study identified close to 300 epigenetic changes associated with altered expression of important metabolic genes, likely contributing to improved insulin sensitivity and lipid metabolism. Pathway enrichment analysis of the 921 differentially expressed and methylated genes indicate that several genes are implicated in the regulation of glycogen metabolism, intracellular signal transduction, and cAMP biosynthesis, reflecting improved skeletal muscle activity and metabolism^61”^.

The sentence “Moreover, the present study found that changes in DNA methylation are associated with reprogramming up to 70% of the transiently altered transcripts, which possibly normalize their expression levels at 52 weeks (Fig. 6)”. now reads “Moreover, the present study found that changes in DNA methylation are associated with reprogramming up to 75% of the transiently altered transcripts, which possibly normalize their expression levels at 52 weeks (Fig. 6)”.

The original version of this Article contained an error in Tables 2 and 3. The correct version of the ‘Number of genes’ row in Table 2 states 25 for Body mass index, 1219 for Fasting glucose, 76 for FFA suppression, 73 for HMW-adiponectin, 29 for Mitochondria content, instead of the original values of 17, 231, 11, 61, and 13, respectively. The correct version of the row ‘Number of genes’ in Table 3 states 177 for Body mass index, 443 for M-value, 163 for Fasting glucose, 112 for FFA suppression, 70 for HMW-adiponectin, and 27 for Mitochondrial content, instead of the original values of 43, 189, 98, 38, 31, and 9, respectively.

The original version of the Supplementary Information associated with this Article contained errors in Supplementary Figs. 5, 7, 9, and 10. The HTML has been updated to include a corrected version of the Supplementary Information; the original incorrect versions of these Figures can be found as Supplementary Information associated with this Correction.

All gene ontology terms depicted in Supplementary Fig. 5 are corrected. In Supplementary Fig. 7, the numbers shown in the Venn diagram as well as the overlapping genes (*PIK3R1, HMGCS2, MAPK10…*) were changed.

Genes shown in Supplementary Fig. 9 “*ASPSCR1*, *NR4A1*, *Elovl5*, *TBC1D1*, *TOMM7* and *FTO*” are replaced by the correct candidates: “*ACACB*, *PTPRE*, *PI3KR1*, *MLXIP*, *IMML2P*, *HMGCS2*”. The correlation plots in Supplementary Fig. 10 were substituted with the corrected ones (from “*ASPSCR1*, *ELOVL5*” to “*PTPRE*, *PIK3R1*”).

All genes shown in Supplementary Tables 1–10 are corrected. We included the following information “Gene names given in italics” in the updated manuscript.

In the updated Supplementary Tables 3–10 the Go-terms used in dedicated figures are highlighted in green. Table annotations had been changed accordingly.

In addition, as part of the re-review to resolve this correction, it was also noticed that multiple correction was not applied to the data. This has now been addressed, and in the Statistical Analysis section of the “Methods”, the following text was added “Correction for multiple testing was performed for the methylome data and not for the transcriptome data. The reason for this is to avoid the number of false negatives and oversee relevant effects according to suggestions of John H. McDonald (McDonald, J.H. 2014. Handbook of Biological Statistics (3rd ed.). Sparky House Publishing, Baltimore, Maryland; p. 254–260). However, we provided the results of the multiple correction in the Supplementary Tables from S11 to 15” in the Statistical Analysis section of the “Methods”.

The current version of the manuscript also adds in the Legend of Fig. 3, the text “For gene expression unadjusted *p*-value and DNA methylation data are adjusted for multiple testing with Benjamini Hochberg correction”. In the legend for Fig. 4 the sentence that read “Up- and downregulated genes are indicated by red and blue signals, respectively. *p < 0.05 (paired t test, *n* = 16, methylation data with Benjamini Hochberg correction)” now reads “Up- and downregulated genes are indicated by red and blue signals, respectively. *p < 0.05 (unadjusted *p*-value paired t test for gene expression, *n* = 16, methylation data with Benjamini Hochberg correction)”. The legend of Fig. 5, where it read “Only significantly differentially methylated CpGs are represented; *p < 0.05 (paired t test, *n* = 16, methylation data with Benjamini Hochberg correction)” now reads “Only significantly differentially methylated CpGs are represented; *p < 0.05 (Gene expression unadjusted *p*-value paired t test, *n* = 16, methylation data with Benjamini Hochberg correction)”. The legend of Fig. 6, where it read “*p < 0.05 (two-tailed paired t test, *n* = 16, methylation data with Benjamini Hochberg correction)” now reads “*p < 0.05 (Gene expression unadjusted *p*-value two-tailed paired t test, *n* = 16, methylation data with Benjamini Hochberg correction) (b)”.

The results of the multiple correction testing, requested by the reviewers, are now included in Supplementary Data 1.

The original version of Table 2 and 3, and those of Figures 3, 4, 5, and 6 have now been replaced with corrected versions.

The previous version of Table 2 was:

**Table 2:** Pearson correlations between gene expression levels and indicated clinical parameters.

	Body mass index	*M*-value	Fasting glucose	FFA suppression	HMW-adiponectin	Mitochondrial content (CSA)
Number of genes	17	27	231	11	61	13

The correct version appears as:

**Table 2:** Pearson correlations between gene expression levels and indicated clinical parameters.

	Body mass index	*M*-value	Fasting glucose	FFA suppression	HMW-adiponectin	Mitochondrial content (CSA)
Number of genes	25	27	1219	76	73	29

The previous version of Table 3 was:

**Table 3:** Number of genes differentially expressed and methylated at 52 and exhibiting at least one CpG significantly correlated to the indicated clinical parameters.

	Body mass index	*M*-value	Fasting glucose	FFA suppression	HMW-adiponectin	Mitochondrial content (CSA)
Number of genes	43	189	98	38	31	9

The correct version appears as:

**Table 3:** Number of genes differentially expressed and methylated at 52 and exhibiting at least one CpG significantly correlated to the indicated clinical parameters.

	Body mass index	*M*-value	Fasting glucose	FFA suppression	HMW-adiponectin	Mitochondrial content (CSA)
Number of genes	177	443	163	112	70	27

The previous version of Figure 3 was:

The correct version appears as:

The previous version of Figure 4 was:

The correct version appears as:

The previous version of Figure 5 was:

The correct version appears as:

The previous version of Figure 6 was:

The correct version appears as:

These errors have been corrected in the HTML and PDF versions of the article. The HTML has been updated to include a corrected version of the Supplementary information.

## Supplementary information

Supplementary information

