# Supplementary material for: Dynamic changes of muscle insulin sensitivity after metabolic surgery
Source: Nat Commun. 2019 Sep 13;10:4179. doi: 10.1038/s41467-019-12081-0 (PMC6744497; doi:10.1038/s41467-019-12081-0)

Supplementary information

Gancheva et al. Dynamic changes of muscle insulin sensitivity after metabolic surgery

Supplementary table 1. Top 13 genes with higher expression in skeletal muscle of OB at 2 weeks after metabolic surgery as compared to baseline. Gene names given in italics.

Gene symbol	Gene name	logFold change	P value	Gene function
CISH	cytokine inducible SH2-containing protein	1.36	0.048	SOCS family proteins form part of a classical negative feedback system that regulates cytokine signal transduction. CIS is involved in the negative regulation of cytokines that signal through the JAK-STAT5 pathway such as erythropoietin, prolactin and interleukin 3 (IL3) receptor. Inhibits STAT5 trans-activation by suppressing its tyrosine phosphorylation.
ANGPTL4	angiopoietin-like 4	1.06	0.045	Mediates inactivation of the lipoprotein lipase LPL, and thereby plays a role in the regulation of triglyceride clearance from the blood serum and in lipid metabolism . May also play a role in regulating glucose homeostasis and insulin sensitivity.
LRRC27	leucine rich repeat containing 27	0.90	0.036	unknown
HMGCS2	3-hydroxy-3-methylglutaryl-CoA synthase 2	0.84	0.003	Catalyzes the first irreversible step in ketogenesis, condensing acetyl-CoA to acetoacetyl-CoA to form HMG-CoA, which is converted by HMG-CoA reductase (HMGCR) into mevalonate.
DNAJC28	DnaJ (Hsp40) homolog subfamily C member 28	0.84	0.039	May have a role in protein folding or as a chaperone.
HS6ST2	heparan sulfate 6-O-sulfotransferase 2	0.82	0.045	6-O-sulfation enzyme which catalyzes the transfer of sulfate from 3'-phosphoadenosine 5'-phosphosulfate (PAPS) to position 6 of the N-sulfoglucosamine residue (GlcNS) of heparan sulfate.
CHAC1	ChaC glutathione-specific gamma-glutamylcyclotransferase 1	0.82	0.020	Catalyzes the cleavage of glutathione into 5-oxo-L-proline and a Cys-Gly dipeptide. Acts specifically on glutathione, but not on other gamma-glutamyl peptides . Negative regulator of Notch signaling pathway involved in embryonic neurogenesis: acts by inhibiting Notch cleavage by furin, maintaining Notch in an immature inactive form, thereby promoting neurogenesis in embryos.
INHBB	inhibin beta B	0.78	0.024	Inhibins and activins inhibit and activate, respectively, the secretion of follitropin by the pituitary gland. Inhibins/activins are involved in regulating a number of diverse functions such as hypothalamic and pituitary hormone secretion, gonadal hormone secretion, germ cell development and maturation, erythroid differentiation, insulin secretion, nerve cell survival, embryonic axial development or bone growth, depending on their subunit composition. Inhibins appear to oppose the functions of activins.
ABCG1	ATP-binding cassette subfamily G member 1	0.76	0.002	Catalyzes the efflux of phospholipids such as sphingomyelin, cholesterol and its oxygenated derivatives like 7beta-hydroxycholesterol and this transport is coupled to hydrlysis of ATP. The lipid efflux is ALB-dependent.
ABCA1	ATP binding cassette subfamily A member 1	0.74	0.006	Catalyzes the translocation of specific phospholipids from the cytoplasmic to the extracellular/lumenal leaflet of membrane coupled to the hydrolysis of ATP. Thereby, participates in phospholipid transfer to apoliproteins to form nascent high density lipoproteins/HDLs . Transports preferentially phosphatidylcholine over phosphatidylserine. May play a similar role in the efflux of intracellular cholesterol to apoliproteins and the formation of nascent high density lipoproteins/HDLs.
AMOTL2	angiomotin like 2	0.71	0.003	Regulates the translocation of phosphorylated SRC to peripheral cell-matrix adhesion sites. Required for proper architecture of actin filaments. Inhibits the Wnt/beta-catenin signaling pathway, probably by recruiting CTNNB1 to recycling endosomes and hence preventing its translocation to the nucleus. Participates in angiogenesis. May play a role in the polarity, proliferation and migration of endothelial cells. Selectively promotes FGF-induced MAPK activation through SRC.

ABCC10	ATP binding cassette subfamily C member 10	0.70	0.023	ATP-dependent transporter probably involved in cellular detoxification through lipophilic anion extrusion.
PCDH15	protocadherin-related 15	0.69	0.009	Calcium-dependent cell-adhesion protein. Essential for maintenance of normal retinal and cochlear function.

Supplementary table 2. Top 13 genes with higher expression in skeletal muscle of OB at 52 weeks after bariatric surgery as compared to baseline. Gene names given in italics.

Gene symbol	Gene name	logFold change	P value	Gene function
BEX2	brain expressed X-linked 2	2.39	0.024	Regulator of mitochondrial apoptosis and G1 cell cycle in breast cancer. Protects the breast cancer cells against mitochondrial apoptosis and this effect is mediated through the modulation of BCL2 protein family, which involves the positive regulation of anti-apoptotic member BCL2 and the negative regulation of pro-apoptotic members BAD, BAK1 and PUMA.
NNMT	nicotinamide N-methyltransferase	1.96	0.007	Catalyzes the N-methylation of nicotinamide and other pyridines to form pyridinium ions. This activity is important for biotransformation of many drugs and xenobiotic compounds.
LAD1	ladinin 1	1.56	0.009	Anchoring filament protein which is a component of the basement membrane zone.
MYH3	myosin, heavy chain 3, skeletal muscle, embryonic	1.47	0.033	Muscle contraction.
FST	follistatin	1.19	0.031	Binds directly to activin and functions as an activin antagonist. Specific inhibitor of the biosynthesis and secretion of pituitary follicle stimulating hormone (FSH).
ACTC1	actin, alpha, cardiac muscle 1	1.18	0.036	Actins are highly conserved proteins that are involved in various types of cell motility and are ubiquitously expressed in all eukaryotic cells.
CHRNA1	cholinergic receptor, nicotinic, alpha 1 (muscle)	1.17	0.047	After binding acetylcholine, the AChR responds by an extensive change in conformation that affects all subunits and leads to opening of an ion-conducting channel across the plasma membrane.
EFCAB7	EF-hand calcium binding domain 7	1.05	0.049	Component of the EvC complex that positively regulates ciliary Hedgehog (Hh) signaling. Required for the localization of the EVC2:EVC subcomplex at the base of primary cilia.
ARMCX4	armadillo repeat containing, X-linked 4	0.95	0.004	unknown
PCDH15	protocadherin-related 15	0.89	0.022	Calcium-dependent cell-adhesion protein. Essential for maintenance of normal retinal and cochlear function.
CCDC8	coiled-coil domain containing 8	0.85	0.038	Core component of the 3M complex, a complex required to regulate microtubule dynamics and genome integrity. It is unclear how the 3M complex regulates microtubules, it could act by controlling the level of a microtubule stabilizer (PubMed:24793695, PubMed:24793696). Required for localization of CUL7 to the centrosome (PubMed:24793695).
ST8SIA1	ST8 alpha-N-acetyl-neuraminide alpha-2,8-sialyltransferase 1	0.82	0.013	Involved in the production of gangliosides GD3 and GT3 from GM3; gangliosides are a subfamily of complex glycosphinglolipds that contain one or more residues of sialic acid.
SMAD9	SMAD family member 9	0.81	0.033	Transcriptional modulator activated by BMP (bone morphogenetic proteins) type 1 receptor kinase. SMAD9 is a receptor-regulated SMAD (R-SMAD).

Supplementary table 3. Gene ontology analysis of differentially expressed genes: CON vs OB (0w)**.**The Go-terms colored in green are depicted in the go plot in fig.3b . Gene names given in italics.

GO-term	Pathway	Gene symbol	Number of genes / total number of genes in pathway	Fold enrichment	P-value
GO:0015671	oxygen transport	*HBM, HBG2, HBG1, HBB, HBA2, HBD*	6 / 15	14.4	3.88E-05
GO:0007165	signal transduction	*CALCOCO1, CXCL9, SLC44A2, PIK3CD, SECTM1, TNFSF13B, RASSF3, IMPA2, RASSF6, CASP1, NCK2, PRKACB, CD53, IL4R, RIPK3, IGFBP5, SP110, TRAF1, ITPKB, TYROBP, TNFRSF25, LTB, S100A9, CSF2RB, PIK3R1, IL2RG, RASGRP2, RASGRP1, LILRA3, FYB, PDE11A, SH3BP1, INPP5D, SPOCK2, SH3BP2, CXCR2, PTK2B, APBB1, PAG1, ASIC1, LYN, CCL23, TXNRD1, TNFRSF10C, WWP1, TNFRSF10B, IGF1, ARHGAP27, NR2F2, ARHGAP25, SORL1, RCAN1, ARHGAP30, IL7R*	54 / 1161	1.7	2.52E-04
GO:0042981	regulation of apoptotic process	*BNIP3L, IGFBP3, LRP5, TNFRSF10C, TNFRSF10B, BMP5, RASSF3, RASSF5, PEA15, RASSF6, CARD17, CASP1, GAS1, TNFRSF25, CARD16, BIRC3, BCL2L1*	17 / 213	2.9	3.17E-04
GO:0006955	immune response	*IL1RN, CXCL9, LST1, SECTM1, IL2RG, FYB, TNFSF13B, ICOS, S1PR4, MAP4K2, HLA-H, CCL23, IL4R, SEMA4D, IL18, TNFRSF10C, TNFRSF10B, IL16, HLA-A, HLA-F, VAV1, TGFBR3, CD27, TNFRSF25, LTB, IL7R*	26 / 421	2.2	3.26E-04
GO:0031032	actomyosin structure organization	*CNN2, MYLIP, EPB41, EPB41L2, MYH9, LURAP1*	6 / 27	8.0	7.92E-04
GO:0043410	positive regulation of MAPK cascade	*BANK1, IGFBP3, LPAR1, CD27, TNFRSF25, IGF1, ZNF622, IL6R, SORBS3*	9 / 81	4.0	1.84E-03
GO:0001771	immunological synapse formation	*DOCK8, NCK2, DOCK2, CORO1A*	4 / 10	14.4	2.22E-03
GO:0006954	inflammatory response	*LYN, CCL23, SP100, CXCL9, ITGB2, IL18, TNFRSF10C, CXCR4, PIK3CD, TNFRSF10B, NMI, NFKB2, CXCR1, CLEC7A, CXCR2, S100A12, CD27, CHI3L1, TNFRSF25, LGALS9, S100A9, S100A8*	22 / 379	2.1	2.25E-03
GO:0030593	neutrophil chemotaxis	*CCL23, ITGB2, CXCR2, S100A12, PIK3CD, S100A9, VAV1, S100A8*	8 / 66	4.3	2.33E-03
GO:0043547	positive regulation of GTPase activity	*DOCK4, TRIO, RGS19, DOCK8, CSF2RB, IL2RG, RASGRP2, RASGRP1, RCBTB2, RGS2, RGS3, SH3BP1, ARHGDIB, NCK2, RAP1GAP2, DENND2D, PLEKHG3, CCL23, SEMA4D, FNBP1, HPS1, ARHGAP27, GMIP, ARHGAP25, VAV1, ARHGAP30, CDC42EP5, ARHGEF2, DOCK2*	29 / 565	1.8	2.42E-03
GO:0050729	positive regulation of inflammatory response	*CCL23, IL18, AGTR1, LPL, S100A12, ZP3, S100A9, S100A8*	8 / 73	3.9	4.12E-03
GO:0045087	innate immune response	*LYN, MAP4K2, NCF2, NLRC5, PIK3CD, ZC3HAV1, CORO1A, SIRT2, NFKB2, RNF135, FGR, TYROBP, CLEC7A, ELF4, LCN2, SERPING1, PTK2B, S100A12, CSK, ANG, ARHGEF2, S100A9, S100A8*	23 / 430	1.9	4.66E-03
GO:0045088	regulation of innate immune response	*RNF135, FGR, IRF1, BIRC3*	4 / 13	11.0	4.96E-03
GO:0043123	positive regulation of I-kappaB kinase/NF-kappaB signaling	*PINK1, SLC44A2, TSPAN6, CASP1, LPAR1, S100A12, TNFRSF10B, SECTM1, ZC3HAV1, LGALS9, LURAP1, BIRC3*	12 / 161	2.7	5.44E-03
GO:0008360	regulation of cell shape	*FGR, SEMA4D, CDC42EP5, LST1, PALM, ITGB2, LPAR1, MYH9, PTK2B, CORO1A, MYL12A*	11 / 140	2.8	5.79E-03
GO:0006334	nucleosome assembly	*HIST1H2BM, HIST1H2BO, HIST1H2BI, HIST1H2BF, HIST1H2BH, HIST1H2BB, HIST1H2BD, HIST1H3D, HIST1H3E, HIST1H2BC*	10 / 119	3.0	6.01E-03
GO:0032496	response to lipopolysaccharide	*CXCL9, CASP1, TNFRSF10C, TNFRSF10B, CD27, TNFRSF25, CSF2RB, LGALS9, S100A8, TIMP4, NFKB2, SNCA*	12 / 164	2.6	6.23E-03
GO:0019731	antibacterial humoral response	*HIST1H2BI, HIST1H2BF, ANG, HLA-A, HIST1H2BD, HIST1H2BC*	6 / 44	4.9	7.24E-03
GO:0060337	type I interferon signaling pathway	*ISG20, HLA-H, SP100, IFITM1, IRF1, HLA-A, HLA-F*	7 / 64	3.9	8.61E-03
GO:0050830	defense response to Gram-positive bacterium	*FGR, HIST1H2BI, HIST1H2BF, ANG, HLA-A, IL6R, HIST1H2BD, HIST1H2BC*	8 / 85	3.4	9.42E-03
GO:0007155	cell adhesion	*DDR1, NLGN4Y, MYBPC2, SEMA4D, ITGB2, LAMB1, EMP2, SPG7, PCDH18, SORBS3, MAG, ARVCF, ISLR, SELL, COL6A2, NINJ1, SSPN, COL6A3, ITGBL1, ACKR3, ITGB7, PLXNC1, MPZL3*	23 / 459	1.8	9.72E-03
GO:0051092	positive regulation of NF-kappaB transcription factor activity	*RIPK3, ITGB2, S100A12, TRAF1, LGALS9, ARHGEF2, RAB7B, S100A9, S100A8, NFKB2*	10 / 133	2.7	1.21E-02
GO:0006796	phosphate-containing compound metabolic process	*PTPRD, IMPA2, INPP5D, LHPP*	4 / 18	8.0	1.28E-02
GO:0060333	interferon-gamma-mediated signaling pathway	*HLA-H, SP100, IRF1, HLA-A, NMI, IFI30, HLA-F*	7 / 71	3.5	1.40E-02
GO:0070098	chemokine-mediated signaling pathway	*CCL23, CXCL9, CXCR1, CXCR2, PTK2B, CXCR4, ACKR3*	7 / 71	3.5	1.40E-02
GO:0030335	positive regulation of cell migration	*LYN, FGR, PAK1, SEMA4D, SEMA3B, GCNT2, PTK2B, PIK3CD, LAMB1, IGF1, PIK3R1, CORO1A*	12 / 184	2.3	1.41E-02
GO:0042127	regulation of cell proliferation	*CNN2, FGR, CXCL9, IL4R, AGTR1, TNFRSF10C, PTK2B, TNFRSF10B, CD27, CSK, TNFRSF25, ARHGEF2*	12 / 185	2.3	1.46E-02
GO:0001867	complement activation, lectin pathway	*FCN1, KRT1, MASP1*	3 / 7	15.4	1.48E-02
GO:0044267	cellular protein metabolic process	*IGFBP5, MMP2, IGFBP3, CSF2RB, IGF1, LMCD1, HIST1H3D, HIST1H3E, SNCA*	9 / 118	2.7	1.73E-02
GO:0035556	intracellular signal transduction	*MAP4K2, IGFBP5, WSB2, MAST3, DGKA, ACOT11, GMIP, FYB, SOCS2, TGFBR3, PINK1, TYROBP, NUAK2, RASSF5, INPP5D, ARHGEF2, ZP3, PLCD4, TNS2, PAG1*	20 / 403	1.8	1.83E-02
GO:0007229	integrin-mediated signaling pathway	*FGR, TYROBP, ITGB2, MYH9, PTK2B, ITGBL1, ITGB7, VAV1*	8 / 99	2.9	2.05E-02
GO:0050900	leukocyte migration	*LYN, MAG, SLC7A7, SELL, INPP5D, ITGB2, MYH9, PIK3R1, MMP9*	9 / 122	2.6	2.07E-02
GO:0031295	T cell costimulation	*LYN, PAK1, CSK, PIK3R1, ICOS, VAV1, TNFSF13B*	7 / 78	3.2	2.14E-02
GO:0007166	cell surface receptor signaling pathway	*CD53, IFITM1, TNFRSF10B, ITPKB, TSPAN15, PTPRC, CXCR1, TSPAN6, P2RY1, CXCR2, PTK2B, CD27, TNFRSF25, IL7R, BIRC3*	15 / 274	2.0	2.17E-02
GO:0070207	protein homotrimerization	*MLKL, ALOX5AP, LCN2, ASIC1*	4 / 22	6.5	2.22E-02
GO:0001666	response to hypoxia	*TGFBR3, PAK1, ALAS2, MMP2, UCP2, CASP1, PTK2B, SCAP, CXCR4, ANG, PPARA*	11 / 172	2.3	2.22E-02
GO:0001558	regulation of cell growth	*SOCS2, DDR1, IGFBP5, IGFBP3, HTRA3, AGTR1, RASGRP2*	7 / 80	3.1	2.39E-02
GO:0002480	sing and presentation of exogenous peptide antigen via MHC class I, TA	*HLA-H, HLA-A, HLA-F*	3 / 9	12.0	2.44E-02
GO:0044342	type B pancreatic cell proliferation	*IGFBP5, IGFBP3, SIDT2*	3 / 9	12.0	2.44E-02
GO:0038083	peptidyl-tyrosine autophosphorylation	*LYN, FGR, DDR1, PTK2B, CSK*	5 / 40	4.5	2.45E-02
GO:0009967	positive regulation of signal transduction	*SOCS2, HOMER1, SH3BP2, SLA, GRAP, PAG1*	6 / 61	3.5	2.71E-02
GO:0006968	cellular defense response	*CXCL9, TYROBP, NCF2, CXCR2, PTK2B, TCIRG1*	6 / 62	3.5	2.88E-02
GO:0051897	positive regulation of protein kinase B signaling	*PINK1, IGFBP5, AKR1C3, IL18, GCNT2, CHI3L1, ZP3*	7 / 84	3.0	2.95E-02
GO:0043534	blood vessel endothelial cell migration	*MYH9, PTK2B, EMP2*	3 / 10	10.8	3.00E-02
GO:0006468	protein phosphorylation	*LYN, MAP4K2, TRIO, MLKL, IGFBP3, PIK3CD, PIK3R1, RUNX3, PHKA2, FYB, ERN1, FGR, PAK1, PINK1, NUAK2, AAK1, PTK2B, CSK, ALPK1, HCST, PRKACB*	21 / 456	1.7	3.10E-02
GO:0031623	receptor internalization	*CXCR1, ITGB2, CXCR2, ACKR3, SNCA*	5 / 43	4.2	3.10E-02
GO:0001816	cytokine production	*PIK3CD, S100A9, RASGRP1, S100A8*	4 / 25	5.7	3.12E-02
GO:0002227	innate immune response in mucosa	*HIST1H2BI, HIST1H2BF, HIST1H2BD, HIST1H2BC*	4 / 25	5.7	3.12E-02
GO:0048013	ephrin receptor signaling pathway	*LYN, ACTR2, PAK1, MMP2, NCK2, MMP9, MYL12A*	7 / 86	2.9	3.26E-02
GO:0007596	blood coagulation	*LYN, NFE2, HBG2, HBG1, DOCK8, IRF1, HBB, HBD, PRKACB, HIST1H3D, HIST1H3E*	11 / 184	2.1	3.33E-02
GO:0007204	positive regulation of cytosolic calcium ion concentration	*CD52, P2RY1, CXCR2, AGTR1, LPAR1, PTK2B, CXCR4, ADRA1D, S1PR4*	9 / 134	2.4	3.37E-02
GO:0032148	activation of protein kinase B activity	*PINK1, IL18, ANG, IGF1*	4 / 26	5.5	3.46E-02
GO:0072661	protein targeting to plasma membrane	*TESC, PTCH1, PALM, MYL12A*	4 / 26	5.5	3.46E-02
GO:0002523	leukocyte migration involved in inflammatory response	*ITGB2, S100A9, S100A8*	3 / 11	9.8	3.60E-02
GO:0051279	regulation of release of sequestered calcium ion into cytosol	*LYN, PTK2B, CORO1A*	3 / 11	9.8	3.60E-02
GO:0016064	immunoglobulin mediated immune response	*IL4R, INPP5D, CD27*	3 / 11	9.8	3.60E-02
GO:0014065	phosphatidylinositol 3-kinase signaling	*PIK3CD, IGF1, PIK3R1, SIRT2*	4 / 27	5.3	3.81E-02
GO:0050832	defense response to fungus	*COTL1, S100A12, S100A9, S100A8*	4 / 27	5.3	3.81E-02
GO:0035987	endodermal cell differentiation	*MMP2, ITGB2, LAMB1, MMP9*	4 / 27	5.3	3.81E-02
GO:0007050	cell cycle arrest	*ERN1, CDKN2C, PCBP4, IRF1, GAS1, APBB1, VASH1, MLF1, UHMK1*	9 / 141	2.3	4.35E-02
GO:0010942	positive regulation of cell death	*UCP2, AKR1C3, HBB, HBA2*	4 / 29	4.9	4.58E-02
GO:0033209	tumor necrosis factor-mediated signaling pathway	*TNFRSF10C, PTK2B, TNFRSF10B, CD27, TNFRSF25, LTB, TNFSF13B, BIRC3*	8 / 118	2.4	4.67E-02
GO:0030198	extracellular matrix organization	*DDR1, COL6A2, SPOCK2, ITGB2, COL6A3, LAMB1, ITGB7, HSD17B12, OLFML2A, MPZL3, NFKB2*	11 / 196	2.0	4.80E-02
GO:0043568	ositive regulation of insulin-like growth factor receptor signaling pathwa	*IGFBP5, IGFBP3, IGF1*	3 / 13	8.3	4.92E-02
GO:0002474	antigen processing and presentation of peptide antigen via MHC class	*HLA-H, SAR1B, HLA-A, HLA-F*	4 / 30	4.8	4.98E-02
GO:0030217	T cell differentiation	*PTPRC, PIK3CD, IL7R, VAV1*	4 / 30	4.8	4.98E-02
GO:0046777	protein autophosphorylation	*ERN1, LYN, FGR, DDR1, PAK1, RIPK3, AAK1, PTK2B, CSK, UHMK1*	10 / 172	2.1	5.14E-02
GO:0045765	regulation of angiogenesis	*SP100, KRT1, PTK2B, EMP2*	4 / 31	4.6	5.41E-02
GO:0006935	chemotaxis	*CCL23, CXCL9, CXCR1, CXCR2, CXCR4, ACKR3, LGALS9, DOCK2*	8 / 122	2.4	5.41E-02
GO:0032796	uropod organization	*MYH9, CORO1A*	2 / 2	35.9	5.48E-02
GO:0070488	neutrophil aggregation	*S100A9, S100A8*	2 / 2	35.9	5.48E-02
GO:1903919	negative regulation of actin filament severing	*CSRP3, MYH9*	2 / 2	35.9	5.48E-02
GO:0038111	interleukin-7-mediated signaling pathway	*IL7R, IL2RG*	2 / 2	35.9	5.48E-02
GO:0038112	interleukin-8-mediated signaling pathway	*CXCR1, CXCR2*	2 / 2	35.9	5.48E-02
GO:0033030	negative regulation of neutrophil apoptotic process	*ITPKB, CXCR2*	2 / 2	35.9	5.48E-02
GO:0070374	positive regulation of ERK1 and ERK2 cascade	*CCL23, P2RY1, FFAR4, GCNT2, PTK2B, CHI3L1, ACKR3, ABCA7, LGALS9, RASGRP1*	10 / 175	2.1	5.61E-02
GO:0034097	response to cytokine	*SP100, IL6R, TIMP4, BCL2L1, NFKB2*	5 / 52	3.5	5.62E-02
GO:0050776	regulation of immune response	*HLA-H, IFITM1, TYROBP, SELL, IRF1, ITGB2, ITGB7, HLA-A, HLA-F, HCST*	10 / 178	2.0	6.12E-02
GO:2001244	positive regulation of intrinsic apoptotic signaling pathway	*RIPK3, S100A9, S100A8, BCL2L1*	4 / 33	4.3	6.30E-02
GO:1902042	e regulation of extrinsic apoptotic signaling pathway via death domain re	*PEA15, TNFRSF10B, ARHGEF2, BMP5*	4 / 33	4.3	6.30E-02
GO:0051604	protein maturation	*TSPAN15, TESC, SORL1*	3 / 15	7.2	6.38E-02
GO:1904707	positive regulation of vascular smooth muscle cell proliferation	*ERN1, MMP2, MMP9*	3 / 15	7.2	6.38E-02
GO:0045947	negative regulation of translational initiation	*BANK1, EIF4EBP1, EIF4EBP3*	3 / 15	7.2	6.38E-02
GO:0007173	epidermal growth factor receptor signaling pathway	*NCK2, PTK2B, CSK, PIK3R1, PAG1*	5 / 56	3.2	7.00E-02
GO:0010744	positive regulation of macrophage derived foam cell differentiation	*IL18, AGTR1, LPL*	3 / 16	6.7	7.16E-02
GO:0001503	ossification	*CLEC3B, IFITM1, RUNX3, MMP9, PDLIM7, BMP5*	6 / 80	2.7	7.20E-02
GO:0006865	amino acid transport	*SLC7A7, SLC43A2, SLC16A10, SLC38A4*	4 / 35	4.1	7.27E-02
GO:0050714	positive regulation of protein secretion	*ANG, APBB1, IGF1, KCNN4*	4 / 36	4.0	7.77E-02
GO:0009409	response to cold	*PLAC8, ACOT11, COX2, LPL*	4 / 36	4.0	7.77E-02
GO:0050731	positive regulation of peptidyl-tyrosine phosphorylation	*SEMA4D, PTK2B, IGF1, ARHGEF2, PLPP3, IL6R*	6 / 82	2.6	7.82E-02
GO:0048535	lymph node development	*RIPK3, LTB, IL7R*	3 / 17	6.3	7.97E-02
GO:0002407	dendritic cell chemotaxis	*CXCR1, CXCR2, CXCR4*	3 / 17	6.3	7.97E-02
GO:1901741	positive regulation of myoblast fusion	*CD53, CXCL9, IL4R*	3 / 17	6.3	7.97E-02

GO:0051056	regulation of small GTPase mediated signal transduction	*RAP1GAP2, ARHGAP30, TRIO, ARHGDIB, ARHGEF2, GMIP, ARHGAP25, VAV1*	8 / 134	2.1	8.05E-02
GO:1903298	gative regulation of hypoxia-induced intrinsic apoptotic signaling pathw	*PINK1, TMBIM6*	2 / 3	23.9	8.11E-02
GO:2000670	positive regulation of dendritic cell apoptotic process	*LYN, LGALS9*	2 / 3	23.9	8.11E-02
GO:0032602	chemokine production	*S100A9, S100A8*	2 / 3	23.9	8.11E-02
GO:1903204	negative regulation of oxidative stress-induced neuron death	*PINK1, FBXO7*	2 / 3	23.9	8.11E-02
GO:0009612	response to mechanical stimulus	*RCAN1, PTCH1, P2RY1, PTK2B, CHI3L1*	5 / 59	3.0	8.14E-02
GO:0042102	positive regulation of T cell proliferation	*PTPRC, NCK2, ZP3, CORO1A, TNFSF13B*	5 / 60	3.0	8.54E-02
GO:0001556	oocyte maturation	*PTK2B, ANG, PABPC1L*	3 / 18	6.0	8.81E-02
GO:0010596	negative regulation of endothelial cell migration	*SP100, NR2F2, VASH1*	3 / 18	6.0	8.81E-02
GO:0002690	positive regulation of leukocyte chemotaxis	*CXCL9, IL6R, GPSM3*	3 / 18	6.0	8.81E-02
GO:0008625	extrinsic apoptotic signaling pathway via death domain receptors	*TNFRSF10C, TNFRSF10B, CD27, PIK3R1*	4 / 38	3.8	8.82E-02
GO:0008285	negative regulation of cell proliferation	*LYN, DDR1, CCL23, IFITM1, CDKN2C, TESC, IGFBP3, SIRT2, BMP5, RASSF5, IRF1, MXI1, NCK2, PTK2B, CSK, FEZF2, TNS2*	17 / 396	1.5	8.96E-02
GO:0051607	defense response to virus	*ISG20, BNIP3L, EXOSC5, IFITM1, CXCL9, PTPRC, IRF1, NLRC5, ZC3HAV1*	9 / 165	2.0	9.01E-02
GO:0007568	aging	*IGFBP5, P2RY1, UCP2, ITGB2, SERPING1, SCAP, EEF2, NFKB2, SNCA*	9 / 165	2.0	9.01E-02
GO:0006928	movement of cell or subcellular component	*ACTR2, MYLIP, PALM, ARHGDIB, IGF1, CORO1A*	6 / 86	2.5	9.15E-02
GO:0045668	negative regulation of osteoblast differentiation	*IGFBP5, SEMA4D, PTCH1, LRP5*	4 / 39	3.7	9.37E-02
GO:0046579	positive regulation of Ras protein signal transduction	*ITPKB, IGF1, RASGRP1*	3 / 19	5.7	9.67E-02
GO:0008589	regulation of smoothened signaling pathway	*PTCH1, TCTN1, GAS1*	3 / 19	5.7	9.67E-02

Supplementary table 4. Gene ontology analysis of differentially expressed genes: OB (0w) vs OB (2w)**.** The GO-terms colored in green are depicted in the go plot in fig.3c. Gene names given in italics.

GO-term	Pathway	Gene symbol	Number of genes / total number of genes in pathway	Fold enrichment	P-value
GO:0000398	mRNA splicing, via spliceosome	*SF3B3, CSTF2, SRRT, EIF4A3, PPWD1, WDR83, ELAVL1, U2AF1L4, SYNCRIP, METTL14, SART3, RAVER1, TRA2B, CWC22, PAPOLA, TXNL4A, HNRNPA1, NCBP1, UPF3B, PRPF40B, GTF2F1, GTF2F2, NUDT21, CD2BP2, BUD13, SNRNP48, GEMIN6, PPIH, HNRNPH3, SLU7*	30 / 222	2.1	2.76E-04
GO:0010501	RNA secondary structure unwinding	*DDX27, DDX25, AGO1, DHX36, EIF4A3, DDX20, DDX31, DDX42, DDX21, DDX10, DDX50*	11 / 44	3.8	4.34E-04
GO:2001235	positive regulation of apoptotic signaling pathway	*ING5, MAGED1, CTSH, INHBB, TP63, CTSC, TGFBR1*	7 / 25	4.3	4.61E-03
GO:0051289	protein homotetramerization	*SRR, PFKL, CRTC3, ACADL, EVL, HPRT1, SOD2, FBP1, TP63, DECR1, THG1L*	11 / 60	2.8	5.12E-03
GO:0007568	aging	*SREBF1, CNP, EPO, ITGB2, TACR3, EEF2, ADRA1A, PPP1R9B, NFKB2, GCLC, SRR, EDNRB, RPS6KB1, P2RY1, KRT25, CANX, NAPEPLD, TSPO, DMD, GCLM, CTSC*	21 / 165	1.9	5.48E-03
GO:0051056	regulation of small GTPase mediated signal transduction	*RAP1GAP2, ARHGAP9, GDI1, ITSN1, ARHGAP19, ARAP3, ARAP1, GMIP, ARHGAP24, ARHGAP4, BCR, SYDE2, FGD3, ABR, RACGAP1, ARHGDIB, RHOU, SIPA1*	18 / 134	2.1	6.37E-03
GO:0006355	regulation of transcription, DNA-templated	*NUP107, JMJD1C, CCAR2, NR3C2, HOXA9, YY2, PTTG1, ZFP30, POFUT1, RUVBL1, ZNF449, HABP4, EP300, ZNF689, SOX6, ZNF324, PDE8A, TSPYL2, TLE2, RFX3, MITF, RUNX3, GTF2F2, ZNF16, ZNF542P, THAP5, ZSCAN21, ZNF558, PREB, HOXB2, UBE2V1, ATF5, ZNF555, CLOCK, ABCG1, ZBTB8A, MAGED1, CREM, CDCA7, ZNF22, PMF1, ZNF26, TAF5L, ZBTB2, PGBD1, ATXN3, ZNF827, ZNF703, ZNF425, ZNF543, MTERF3, CNOT10, SMAD5, NFKB2, ZBTB6, ZZZ3, YEATS2, ZNF814, TADA1, ZNF775, ZNF774, SLC2A4RG, ZSCAN2, BNC2, PHF20, CHD9, TFB1M, AFF4, SNAPC5, LBH, ZNF805, MECOM, SIX4, ZNF529, ZNF407, ZNF527, ZNF761, CNOT6L, NCOA5, TSC22D2, IL16, PPHLN1, TGFBR1, TOX2, IRF2, ZNF239, ZNF358, ZNF511, VGLL3, ZNF232, KANK1, RGS19, PRKAA2, USP27X, SRRT, ZBTB43, NR2C2, MLLT3, NR2C1, ZBTB41, ZNF7, ZBTB40, ZNF507, PPP1R8, ZNF746, DMD, ZNF226, ZNF343, ZNF100, RNF20, PRRX1, ZNF184, UTP4, ZNF181, YLPM1, GTF2H3, PML, ZNF33B, KLF8, ASXL2, ZNF613, ESF1*	122 / 1504	1.2	8.40E-03
GO:0007010	cytoskeleton organization	*TBCCD1, MAST3, SH3KBP1, ARAP3, CAMSAP1, ACTB, ARHGAP4, CNN2, FGD3, CECR2, TUBB2B, STRIP2, ZMYM3, GAN, KRT25, RHOU, DMD, BRWD3, SIPA1, SH3D19*	20 / 161	1.9	8.83E-03
GO:0006364	rRNA processing	*UTP15, WDR36, DDX27, UTP4, PNO1, RPL31, WDR3, RPL12, EIF4A3, DDX21, NOLC1, NOL9, WDR12, RBFA, WDR43, BYSL, BOP1, EBNA1BP2, NOB1, DKC1, RIOK2, RIOK1, RPL28, DCAF13*	24 / 214	1.7	1.27E-02
GO:0006270	DNA replication initiation	*ORC5, PURA, POLA1, MCM3, MCM4, MCM6, MCM2*	7 / 32	3.3	1.60E-02
GO:0006750	glutathione biosynthetic process	*GGT5, GCLC, HAGH, CHAC1, GCLM*	5 / 16	4.8	1.75E-02
GO:0006461	protein complex assembly	*CD74, SF3B3, NIFK, GPAA1, FANCC, MITF, PML, TAPBP, SLC7A6, CCNB1, SLC7A7, PPP2R1B, ZW10, PPIH, KCNQ5*	15 / 116	2.0	1.88E-02
GO:0008360	regulation of cell shape	*TBCCD1, SH3KBP1, LST1, ITGB2, ARAP3, ARAP1, CORO1A, MYL12A, FGD3, FGR, STRIP2, FES, ANXA7, PLEKHO1, RHOU, MYH10, BRWD3*	17 / 140	1.9	2.03E-02
GO:0001932	regulation of protein phosphorylation	*LYN, SOCS1, CCDC88C, TADA3, TLR7, PML, PPP1R9B*	7 / 34	3.2	2.13E-02
GO:0006357	regulation of transcription from RNA polymerase II promoter	*GMEB1, CBFB, EPO, MAGED1, TSHZ1, CITED4, GLIS3, PURA, TCEAL6, TCEAL5, ZMYM3, HSF2, CREG1, RUVBL1, GTF2IRD2B, SMARCE1, BRD3, IFNAR2, SREBF1, ZNF143, ANXA4, ACTL6A, TCF12, VEZF1, MITF, ELP4, FOXP4, SOD2, RUNX3, NFKB2, TADA2B, CTDSP1, MED21, TADA3, TADA1, IRF5, ATF5, CLOCK, BRWD3, ZSCAN29, VGLL3*	41 / 441	1.4	2.20E-02
GO:0006334	nucleosome assembly	*TSPYL2, HIST1H2BM, SHPRH, SET, HIST1H2BI, HIST2H2BE, SART3, HIST1H1D, HIST1H4H, H1FX, HIST1H2BD, HIST1H3D, HIST1H4F, MCM2, TSPYL5*	15 / 119	1.9	2.30E-02
GO:0040001	establishment of mitotic spindle localization	*NUSAP1, CLASP1, KPNB1*	3 / 4	11.5	2.34E-02
GO:1900034	regulation of cellular response to heat	*DNAJC2, NUP107, SEH1L, RPA3, TPR, CHORDC1, MAPKAPK2, EP300, NUP54, CCAR2, ATR*	11 / 75	2.2	2.34E-02
GO:0006268	DNA unwinding involved in DNA replication	*PURA, MCM4, MCM6, MCM2*	4 / 10	6.1	2.35E-02
GO:0006260	DNA replication	*ACHE, SUPT16H, SET, PCNA, ORC5, ING5, POLA1, DNAJC2, RPA3, MCM3, TIMELESS, MCM4, NT5M, MCM6, DTD1, ATR, MCM2, RHNO1*	18 / 155	1.8	2.46E-02
GO:0060048	cardiac muscle contraction	*MYBPC3, CSRP3, VEGFB, DMD, TAZ, SMAD5, MYH6, MYH7*	8 / 45	2.7	2.55E-02
GO:0007080	mitotic metaphase plate congression	*CCNB1, SEH1L, ZW10, CHMP2B, RRS1, KIF22, KPNB1*	7 / 37	2.9	3.12E-02
GO:0034605	cellular response to heat	*LYN, ATXN3, HDAC2, ST8SIA1, TPR, NF1, SLU7*	7 / 37	2.9	3.12E-02
GO:0008283	cell proliferation	*KHDRBS1, ACHE, PCNA, PAK1IP1, SRRT, ADRA1D, PDS5B, GNAI2, FRAT2, CREG1, PPP1R8, ERBB2, TSPO, ANXA7, MYH10, MAP4K1, IFNAR2, CD74, USP8, SPHK2, XRCC5, WDR12, BYSL, GNL3, TRAIP, BOP1, POLA1, FES, DKC1, IRF2, MAP3K11, SIPA1, RAPGEF3, ZPR1*	34 / 366	1.4	3.71E-02
GO:0001919	regulation of receptor recycling	*ACHE, KIF16B, LAMTOR1*	3 / 5	9.2	3.73E-02
GO:0046627	negative regulation of insulin receptor signaling pathway	*KANK1, PTPRE, SOCS1, CISH, RPS6KB1, NCOA5*	6 / 29	3.2	3.77E-02
GO:0006801	superoxide metabolic process	*IMMP2L, SH3PXD2A, NOX4, CYBA, SOD2*	5 / 20	3.8	3.78E-02
GO:0006120	mitochondrial electron transport, NADH to ubiquinone	*NDUFS8, NDUFB5, NDUFB11, NDUFA3, NDUFAB1, ND1, NDUFV1, ND2*	8 / 49	2.5	3.86E-02
GO:0021762	substantia nigra development	*LDHA, CNP, HSPA5, CALM3, CKB, CALM2, ACTB, COX6B1*	8 / 49	2.5	3.86E-02
GO:0000226	microtubule cytoskeleton organization	*EML4, ATXN3, CNP, CNTN2, MAP6D1, TUBG1, MID1, CLASP1, MARK2, ZPR1*	10 / 71	2.2	4.07E-02
GO:0031124	mRNA 3'-end processing	*NUDT21, NCBP1, CSTF2, EIF4A3, PAPOLA, UPF3B, SLU7, THOC7*	8 / 50	2.4	4.25E-02
GO:0008380	RNA splicing	*RBFOX2, SF3B3, NCBP1, MTERF3, EIF4A3, CCAR2, THOC7, IWS1, SRPK1, PPP1R9B, AFF2, SYNCRIP, PPP1R8, ZNF326, SNRNP48, PPIG, HNRNPH3, ZPR1*	18 / 166	1.7	4.35E-02
GO:0071480	cellular response to gamma radiation	*XRCC5, NOX4, CYBA, ATR, TSPYL5*	5 / 21	3.6	4.43E-02
GO:0019722	calcium-mediated signaling	*AGTR1, CXCR4, CALM3, CCR5, CALM2, LACRT, CAMKK2, PPP1R9B*	8 / 51	2.4	4.66E-02
GO:0007169	transmembrane receptor protein tyrosine kinase signaling pathway	*LYN, FGR, DDR1, MUSK, ERBB2, CD7, ABL2, LCP2, CSK, GAB2, ANGPTL1, MTSS1*	12 / 96	1.9	4.80E-02
GO:0007512	adult heart development	*ADRA1A, MYH10, MYH6, MYH7*	4 / 13	4.7	4.85E-02
GO:0045088	regulation of innate immune response	*FGR, TKFC, IRF1, XIAP*	4 / 13	4.7	4.85E-02
GO:0006351	transcription, DNA-templated	*CRTC3, JMJD1C, CCAR2, NR3C2, HOXA9, YY2, ZFP30, RUVBL1, ZNF449, HABP4, ZNF326, ZNF689, SOX6, ZNF324, TP63, TSPYL2, TLE4, TLE2, ZNF281, KCTD1, RFX3, FOXP4, ILF2, PIAS2, ZNF16, ZNF542P, EID2, THAP5, ZSCAN21, ERF, TIMELESS, ZNF558, PREB, HOXB2, ZNF555, CLOCK, ZSCAN29, KHDRBS1, ZBTB8A, TSHZ1, CREM, CDCA7, ZNF22, ZNF26, ZBTB2, ATXN3, ZNF827, DHX36, ZNF703, ZNF425, GTF2IRD2B, ZNF543, XRCC5, SNAI3, CNOT10, MINA, SMAD5, TADA2B, ZBTB6, ZZZ3, AGO1, TADA3, TADA1, ZNF775, ZNF774, SLC2A4RG, ZSCAN2, BNC2, PHF20, CHD9, CITED4, CHD6, NKAP, MED19, TFB1M, ING5, CSRP3, LBH, ZNF805, MECOM, VPS72, ZNF529, ZNF407, ZNF527, YAF2, ZNF761, BRD3, TSC22D4, CNOT6L, NCOA5, ACTL6A, IL16, PPHLN1, PPRC1, KLF16, DNAJC2, RIPPLY3, TOX2, MED21, ZNF239, IRF5, ZNF358, ZNF511, VGLL3, ZNF232, KANK1, GTF3C3, HDAC2, PRKAA2, ZBTB43, ZBTB42, NR2C2, MLLT3, NR2C1, ZBTB41, IWS1, ZNF7, ZBTB40, PURA, TCEAL6, TCEAL5, ZNF507, TP53INP2, ERBB2, ATXN1L, PPP1R8, ZNF746, E2F3, ZNF226, ZNF343, ZNF100, KDM4A, ZNF184, UTP4, ZNF181, YLPM1, PML, ZNF33B, TBX2, KLF8, ASXL2, ZNF613, ESF1, RGS12, NAA15, NEUROG2*	146 / 1955	1.1	5.08E-02
GO:0032981	mitochondrial respiratory chain complex I assembly	*NDUFS8, NDUFB5, NDUFB11, NDUFA3, NDUFAB1, ND1, TAZ, NDUFV1, ND2*	9 / 63	2.2	5.15E-02
GO:0006368	transcription elongation from RNA polymerase II promoter	*PCID2, SUPT16H, TAF12, NCBP1, LEO1, ELP4, GTF2F1, GTF2H3, GTF2F2, TAF3, THOC7*	11 / 86	2.0	5.31E-02
GO:0031442	positive regulation of mRNA 3'-end processing	*CCNB1, NCBP1, LEO1*	3 / 6	7.7	5.35E-02
GO:0009437	carnitine metabolic process	*SLC22A4, SLC22A5, CROT*	3 / 6	7.7	5.35E-02
GO:0006999	nuclear pore organization	*SEH1L, TPR, NUP54*	3 / 6	7.7	5.35E-02
GO:0045727	positive regulation of translation	*RPS6KB1, ERBB2, EIF4A3, CIRBP, UPF3B, EEF2, ELAVL1, RBMS3*	8 / 53	2.3	5.55E-02
GO:0042127	regulation of cell proliferation	*RBFOX2, CDCA7, XIAP, PDS5B, NDRG1, PPP1R9B, GNL3, CNN2, FGR, PURA, EID2, FES, RPA3, MZB1, TIMELESS, AGTR1, ABL2, CSK, TNFRSF25*	19 / 185	1.6	5.84E-02
GO:0007064	mitotic sister chromatid cohesion	*POGZ, PDS5B, SMC3, WAPL*	4 / 14	4.4	5.88E-02
GO:0032968	tive regulation of transcription elongation from RNA polymerase II prom	*SUPT16H, LEO1, GTF2F1, GTF2F2*	4 / 14	4.4	5.88E-02
GO:0014823	response to activity	*GCLC, SCO2, UQCRC1, CYBA, SOD2, GCLM, SLC25A25*	7 / 43	2.5	5.90E-02
GO:1904837	beta-catenin-TCF complex assembly	*TLE4, TLE2, RUVBL1, LEO1, EP300, HIST1H4H, HIST1H4F*	7 / 43	2.5	5.90E-02
GO:0043966	histone H3 acetylation	*ING5, TAF12, YEATS2, TADA3, TADA1, ELP4, TAF5L*	7 / 43	2.5	5.90E-02
GO:0043044	ATP-dependent chromatin remodeling	*SMARCE1, CECR2, HDAC2, ACTL6A, ACTB*	5 / 23	3.3	5.92E-02
GO:0002027	regulation of heart rate	*POPDC2, CALM3, DMD, CALM2, MYH6, MYH7*	6 / 33	2.8	6.09E-02
GO:0006406	mRNA export from nucleus	*NUP107, NXT1, SEH1L, NCBP1, DDX25, TPR, EIF4A3, UPF3B, BUD13, NUP54, SLU7, THOC7*	12 / 100	1.8	6.12E-02
GO:0042493	response to drug	*HDAC2, ABCD3, HTR2C, SLC1A3, SLC6A2, ADRA1A, SRR, LDHA, CCNB1, TSPO, PMS2, PMS1, ABCA1, LYN, SREBF1, XRCC5, BAD, VEGFB, GGH, CYBA, SOD2, DHODH, NPC1, RPS6KB1, LCN2, ND1, GCLM, ATR*	28 / 304	1.4	6.38E-02
GO:0006405	RNA export from nucleus	*NXT1, NCBP1, TPR, EIF4A3, UPF3B, HNRNPA1, SLU7, THOC7*	8 / 55	2.2	6.54E-02
GO:0000082	G1/S transition of mitotic cell cycle	*ORC5, POLA1, PCNA, RPS6KB1, RPA3, EIF4EBP1, MCM3, MCM4, RHOU, MCM6, CDKN3, MCM2*	12 / 102	1.8	6.86E-02
GO:0006897	endocytosis	*SORT1, FNBP1, SGIP1, SH3KBP1, LRP4, C9ORF72, EHD1, EEA1, SNX4, NECAP1, NPC1, PACSIN3, RIN3, LDLR, RAB7A*	15 / 139	1.7	6.99E-02
GO:0031167	rRNA methylation	*TRMT112, NSUN4, DIMT1, TFB1M*	4 / 15	4.1	7.00E-02

GO:0006309	apoptotic DNA fragmentation	*CECR2, DFFA, DNASE2B, KPNB1*	4 / 15	4.1	7.00E-02
GO:0006691	leukotriene metabolic process	*GGT5, ALOX5AP, MAPKAPK2, LTA4H*	4 / 15	4.1	7.00E-02
GO:0014898	cardiac muscle hypertrophy in response to stress	*KDM4A, INPP5F, MYH6, MYH7*	4 / 15	4.1	7.00E-02
GO:0045947	negative regulation of translational initiation	*TPR, EIF4EBP1, EIF4EBP2, EIF4EBP3*	4 / 15	4.1	7.00E-02
GO:0038066	p38MAPK cascade	*MAPKAPK2, LGALS9, NR2C2*	3 / 7	6.6	7.18E-02
GO:0006282	regulation of DNA repair	*UBE2N, USP1, UBE2V1*	3 / 7	6.6	7.18E-02
GO:0048841	regulation of axon extension involved in axon guidance	*PLXND1, PLXNA2, PLXNC1*	3 / 7	6.6	7.18E-02
GO:0030388	fructose 1,6-bisphosphate metabolic process	*PFKL, ALDOC, FBP1*	3 / 7	6.6	7.18E-02
GO:0051301	cell division	*DCLRE1A, ANAPC13, SDCCAG3, SEH1L, ZWILCH, CETN3, CDCA7, PDS5B, SMC3, PMF1, GNAI2, SMC2, CCNB3, PPP1CB, CCNB1, PTTG1, TPR, POGZ, RUVBL1, TXNL4A, CLASP1, NEK4, DYNLT3, WAPL, HAUS2, ZNF16, STAG1, FAM175B, ZW10, TIMELESS, ARF6*	31 / 350	1.4	7.64E-02
GO:0000375	RNA splicing, via transesterification reactions	*SF3B3, TRA2B, WDR83, TXNL4A, SLU7*	5 / 25	3.1	7.65E-02
GO:0042254	ribosome biogenesis	*BOP1, LSG1, DDX31, URB2, MINA, GNL3*	6 / 36	2.6	8.26E-02
GO:0042795	snRNA transcription from RNA polymerase II promoter	*SNAPC5, ZNF143, NCBP1, SRRT, INTS5, INTS7, RPRD1B, GTF2F1, GTF2F2*	9 / 70	2.0	8.49E-02
GO:0006890	retrograde vesicle-mediated transport, Golgi to ER	*RINT1, RACGAP1, KIF3A, PITPNB, ZW10, COG3, COPG2, KIF22, DNAJC28, TAPBP*	10 / 82	1.9	8.59E-02
GO:0071407	cellular response to organic cyclic compound	*CCNB1, RGS19, RPS6KB1, TIPARP, P2RY1, AXIN1, CYBA, SMAD5*	8 / 59	2.1	8.83E-02
GO:0010467	gene expression	*METTL14, NCBP1, NOX4, LTB, GTF2F1, HNRNPA1, GTF2F2*	7 / 48	2.2	9.06E-02
GO:0098655	cation transmembrane transport	*SLC22A4, SLC22A5, TMEM63C, SLC41A3, GAR1, ANO10, FAM26F*	7 / 48	2.2	9.06E-02
GO:0006002	fructose 6-phosphate metabolic process	*PFKL, FBP1, PFKM*	3 / 8	5.7	9.17E-02
GO:0017183	peptidyl-diphthamide biosynthetic process from peptidyl-histidine	*DPH2, DPH6, EEF2*	3 / 8	5.7	9.17E-02
GO:0098789	pre-mRNA cleavage required for polyadenylation	*NCBP1, CSTF2, CSTF2T*	3 / 8	5.7	9.17E-02
GO:0034383	low-density lipoprotein particle clearance	*EHD1, DGAT2, LDLR*	3 / 8	5.7	9.17E-02
GO:0006337	nucleosome disassembly	*SMARCE1, SUPT16H, SET, HIST3H2A*	4 / 17	3.6	9.51E-02
GO:0006400	tRNA modification	*METTL1, URM1, TRMT6, AARS, THG1L*	5 / 27	2.8	9.59E-02
GO:0042752	regulation of circadian rhythm	*PPP1CB, PRKAA2, MAGED1, TIMELESS, CREM, PML, CCAR2*	7 / 49	2.2	9.79E-02

Supplementary table 5. Gene ontology analysis of transiently differentially expressed genes OB (0w) vs OB (2w). The GO-terms colored in green are depicted in the go plot in suppl.fig.5a**.** Gene names given in italics.

GO-term	Pathway	Gene symbol	Number of genes / total number of genes in pathway	Fold enrichment	P-value
GO:0000398	mRNA splicing, via spliceosome	*SF3B3, CSTF2, SRRT, EIF4A3, PPWD1, WDR83, ELAVL1, U2AF1L4, SYNCRIP, METTL14, SART3, RAVER1, TRA2B, CWC22, PAPOLA, TXNL4A, HNRNPA1, NCBP1, UPF3B, GTF2F1, GTF2F2, NUDT21, BUD13, SNRNP48, GEMIN6, PPIH, HNRNPH3, SLU7*	28 / 222	2.4	5.11E-05
GO:0010501	RNA secondary structure unwinding	*DDX27, DDX25, AGO1, DHX36, EIF4A3, DDX20, DDX31, DDX42, DDX21, DDX10, DDX50*	11 / 44	4.7	8.10E-05
GO:0006364	rRNA processing	*UTP15, WDR36, DDX27, UTP4, PNO1, RPL31, WDR3, RPL12, EIF4A3, DDX21, NOLC1, NOL9, WDR12, RBFA, WDR43, BYSL, BOP1, EBNA1BP2, NOB1, DKC1, RIOK2, RIOK1, RPL28, DCAF13*	24 / 214	2.1	1.05E-03
GO:0051289	protein homotetramerization	*SRR, PFKL, CRTC3, ACADL, EVL, HPRT1, SOD2, TP63, DECR1, THG1L*	10 / 60	3.1	4.17E-03
GO:0007568	aging	*SREBF1, CNP, EPO, TACR3, EEF2, ADRA1A, PPP1R9B, NFKB2, GCLC, SRR, EDNRB, RPS6KB1, KRT25, CANX, NAPEPLD, TSPO, DMD, GCLM*	18 / 165	2.1	6.81E-03
GO:0060048	cardiac muscle contraction	*MYBPC3, CSRP3, VEGFB, DMD, TAZ, SMAD5, MYH6, MYH7*	8 / 45	3.3	9.04E-03
GO:0051301	cell division	*DCLRE1A, ANAPC13, SDCCAG3, SEH1L, ZWILCH, CETN3, CDCA7, PDS5B, SMC3, PMF1, GNAI2, SMC2, CCNB3, PPP1CB, CCNB1, PTTG1, TPR, POGZ, RUVBL1, TXNL4A, NEK4, DYNLT3, WAPL, HAUS2, ZNF16, STAG1, FAM175B, ZW10, TIMELESS, ARF6*	30 / 350	1.6	1.19E-02
GO:0006406	mRNA export from nucleus	*NUP107, NXT1, SEH1L, NCBP1, DDX25, TPR, EIF4A3, UPF3B, BUD13, NUP54, SLU7, THOC7*	12 / 100	2.3	1.70E-02
GO:0006355	regulation of transcription, DNA-templated	*NUP107, JMJD1C, CCAR2, NR3C2, HOXA9, YY2, PTTG1, ZFP30, POFUT1, RUVBL1, ZNF449, HABP4, EP300, ZNF689, SOX6, ZNF324, PDE8A, TSPYL2, RFX3, MITF, RUNX3, GTF2F2, ZNF16, ZNF542P, THAP5, ZSCAN21, ZNF558, PREB, HOXB2, ATF5, ZNF555, CLOCK, ZBTB8A, CREM, CDCA7, ZNF22, PMF1, TAF5L, ZBTB2, ATXN3, ZNF827, ZNF703, ZNF425, ZNF543, MTERF3, CNOT10, SMAD5, NFKB2, ZBTB6, ZZZ3, YEATS2, ZNF814, TADA1, ZNF774, SLC2A4RG, ZSCAN2, BNC2, PHF20, CHD9, TFB1M, AFF4, SNAPC5, LBH, ZNF805, MECOM, SIX4, ZNF529, ZNF407, ZNF527, ZNF761, CNOT6L, NCOA5, TSC22D2, PPHLN1, TGFBR1, TOX2, ZNF239, ZNF358, ZNF511, ZNF232, KANK1, PRKAA2, USP27X, SRRT, NR2C2, ZBTB41, ZBTB40, ZNF507, ZNF746, DMD, ZNF343, RNF20, ZNF184, UTP4, ZNF181, YLPM1, ZNF33B, KLF8, ASXL2, ESF1*	100 / 1504	1.3	1.45E-02
GO:0008380	RNA splicing	*RBFOX2, SF3B3, NCBP1, MTERF3, EIF4A3, CCAR2, THOC7, IWS1, SRPK1, PPP1R9B, AFF2, SYNCRIP, ZNF326, SNRNP48, PPIG, HNRNPH3, ZPR1*	17 / 166	1.9	1.55E-02
GO:0019722	calcium-mediated signaling	*AGTR1, CXCR4, CALM3, CCR5, CALM2, LACRT, CAMKK2, PPP1R9B*	8 / 51	2.9	1.76E-02
GO:0006890	retrograde vesicle-mediated transport, Golgi to ER	*RINT1, RACGAP1, KIF3A, PITPNB, ZW10, COG3, COPG2, KIF22, DNAJC28, TAPBP*	10 / 82	2.3	2.96E-02
GO:0010467	gene expression	*METTL14, NCBP1, NOX4, LTB, GTF2F1, HNRNPA1, GTF2F2*	7 / 48	2.7	4.05E-02
GO:0098655	cation transmembrane transport	*SLC22A4, SLC22A5, TMEM63C, SLC41A3, GAR1, ANO10, FAM26F*	7 / 48	2.7	4.05E-02
GO:0046627	negative regulation of insulin receptor signaling pathway	*KANK1, SOCS1, CISH, RPS6KB1, NCOA5*	5 / 29	3.2	6.54E-02
GO:0051056	regulation of small GTPase mediated signal transduction	*ARHGAP9, GDI1, ITSN1, ARHGAP19, ARAP3, ARAP1, GMIP, ARHGAP24, ARHGAP4, SYDE2, FGD3, ABR, RACGAP1, RHOU*	14 / 134	2.0	2.60E-02
GO:0007512	adult heart development	*ADRA1A, MYH10, MYH6, MYH7*	4 / 13	5.8	2.86E-02
GO:0007080	mitotic metaphase plate congression	*CCNB1, SEH1L, ZW10, CHMP2B, RRS1, KIF22, KPNB1*	7 / 37	3.6	1.25E-02
GO:0031124	mRNA 3'-end processing	*NUDT21, NCBP1, CSTF2, EIF4A3, PAPOLA, UPF3B, SLU7, THOC7*	8 / 50	3.0	1.58E-02
GO:1900034	regulation of cellular response to heat	*DNAJC2, NUP107, SEH1L, RPA3, TPR, CHORDC1, EP300, NUP54, CCAR2, ATR*	10 / 75	2.5	1.76E-02
GO:0032968	tive regulation of transcription elongation from RNA polymerase II prom	*SUPT16H, LEO1, GTF2F1, GTF2F2*	4 / 14	5.4	3.50E-02
GO:0007064	mitotic sister chromatid cohesion	*POGZ, PDS5B, SMC3, WAPL*	4 / 14	5.4	3.50E-02
GO:0031442	positive regulation of mRNA 3'-end processing	*CCNB1, NCBP1, LEO1*	3 / 6	9.4	3.67E-02
GO:0006999	nuclear pore organization	*SEH1L, TPR, NUP54*	3 / 6	9.4	3.67E-02
GO:0009437	carnitine metabolic process	*SLC22A4, SLC22A5, CROT*	3 / 6	9.4	3.67E-02
GO:0006405	RNA export from nucleus	*NXT1, NCBP1, TPR, EIF4A3, UPF3B, HNRNPA1, SLU7, THOC7*	8 / 55	2.7	2.57E-02
GO:0002027	regulation of heart rate	*POPDC2, CALM3, DMD, CALM2, MYH6, MYH7*	6 / 33	3.4	2.88E-02
GO:0042795	snRNA transcription from RNA polymerase II promoter	*SNAPC5, ZNF143, NCBP1, SRRT, INTS5, INTS7, RPRD1B, GTF2F1, GTF2F2*	9 / 70	2.4	3.16E-02
GO:0006368	transcription elongation from RNA polymerase II promoter	*PCID2, SUPT16H, TAF12, NCBP1, LEO1, ELP4, GTF2F1, GTF2F2, TAF3, THOC7*	10 / 86	2.2	3.86E-02
GO:0009791	post-embryonic development	*RAB3A, TIPARP, IMPAD1, CCDC47, SOX6, SOD2, ATF5, TGFBR1, RC3H2*	9 / 73	2.3	3.93E-02
GO:0045947	negative regulation of translational initiation	*TPR, EIF4EBP1, EIF4EBP2, EIF4EBP3*	4 / 15	5.0	4.21E-02
GO:0014898	cardiac muscle hypertrophy in response to stress	*KDM4A, INPP5F, MYH6, MYH7*	4 / 15	5.0	4.21E-02
GO:0031167	rRNA methylation	*TRMT112, NSUN4, DIMT1, TFB1M*	4 / 15	5.0	4.21E-02
GO:0006461	protein complex assembly	*CD74, SLC7A6, CCNB1, SF3B3, NIFK, PPP2R1B, GPAA1, ZW10, FANCC, PPIH, MITF, TAPBP*	12 / 116	1.9	4.44E-02
GO:0006357	regulation of transcription from RNA polymerase II promoter	*GMEB1, CBFB, EPO, TSHZ1, GLIS3, PURA, HSF2, CREG1, RUVBL1, SMARCE1, BRD3, SREBF1, ZNF143, ANXA4, ACTL6A, TCF12, VEZF1, MITF, ELP4, FOXP4, SOD2, RUNX3, NFKB2, TADA2B, CTDSP1, MED21, TADA3, TADA1, IRF5, ATF5, CLOCK, BRWD3, ZSCAN29*	33 / 441	1.4	4.61E-02
GO:0007010	cytoskeleton organization	*TBCCD1, MAST3, ARAP3, ACTB, ARHGAP4, CNN2, FGD3, TUBB2B, STRIP2, GAN, KRT25, RHOU, DMD, BRWD3, SH3D19*	15 / 161	1.8	4.78E-02
GO:0000375	RNA splicing, via transesterification reactions	*SF3B3, TRA2B, WDR83, TXNL4A, SLU7*	5 / 25	3.8	4.11E-02
GO:0006351	transcription, DNA-templated	*CRTC3, JMJD1C, CCAR2, NR3C2, HOXA9, YY2, ZFP30, RUVBL1, ZNF449, HABP4, ZNF326, ZNF689, SOX6, ZNF324, TP63, TSPYL2, TLE4, ZNF281, KCTD1, RFX3, FOXP4, PIAS2, ZNF16, ZNF542P, EID2, THAP5, ZSCAN21, ERF, TIMELESS, ZNF558, PREB, HOXB2, ZNF555, CLOCK, ZSCAN29, KHDRBS1, ZBTB8A, TSHZ1, CREM, CDCA7, ZNF22, ZBTB2, ATXN3, ZNF827, DHX36, ZNF703, ZNF425, ZNF543, SNAI3, CNOT10, MINA, SMAD5, TADA2B, ZBTB6, ZZZ3, AGO1, TADA3, TADA1, ZNF774, SLC2A4RG, ZSCAN2, BNC2, PHF20, CHD9, CHD6, NKAP, TFB1M, CSRP3, LBH, ZNF805, MECOM, VPS72, ZNF529, ZNF407, ZNF527, YAF2, ZNF761, BRD3, TSC22D4, CNOT6L, NCOA5, ACTL6A, PPHLN1, PPRC1, KLF16, DNAJC2, RIPPLY3, TOX2, MED21, ZNF239, IRF5, ZNF358, ZNF511, ZNF232, KANK1, GTF3C3, HDAC2, PRKAA2, ZBTB42, NR2C2, ZBTB41, IWS1, ZBTB40, PURA, ZNF507, ATXN1L, ZNF746, ZNF343, KDM4A, ZNF184, UTP4, ZNF181, YLPM1, ZNF33B, TBX2, KLF8, ASXL2, ESF1, RGS12, NAA15, NEUROG2*	121 / 1955	1.2	4.84E-02
GO:0048841	regulation of axon extension involved in axon guidance	*PLXND1, PLXNA2, PLXNC1*	3 / 7	8.1	4.95E-02
GO:0006750	glutathione biosynthetic process	*GGT5, GCLC, CHAC1, GCLM*	4 / 16	4.7	4.99E-02
GO:0019827	stem cell population maintenance	*EIF4ENIF1, METTL14, MED21, LEO1, NKAP, ZNF358, SMC3*	7 / 50	2.6	4.81E-02
GO:0006369	termination of RNA polymerase II transcription	*NUDT21, NCBP1, CSTF2, EIF4A3, PAPOLA, UPF3B, SLU7, THOC7*	8 / 64	2.4	5.24E-02
GO:0006397	mRNA processing	*KHDRBS1, RBFOX2, SF3B3, CNOT6L, TSEN15, HNRNPLL, CCAR2, THOC7, IWS1, SRPK1, AFF2, NUDT21, AGGF1, ZNF326, HNRNPA1, ZPR1*	16 / 179	1.7	5.43E-02
GO:0000302	response to reactive oxygen species	*TXNRD2, TRA2B, GPX7, NOX4, CYBA, SOD2*	6 / 39	2.9	5.40E-02
GO:0006457	protein folding	*MESDC2, HSP90AA2P, PPWD1, CCT6B, HSP90B1, GNAI2, DNAJA1, TMX1, GNB2, DNAJC21, DNAJB11, CANX, PPIF, PPIH, PPIG, CCT5*	16 / 180	1.7	5.64E-02
GO:0045666	positive regulation of neuron differentiation	*SPAG9, KDM4A, IMPACT, TCF12, VWC2L, BNIP2, DMD, TCF4, BRINP3*	9 / 78	2.2	5.46E-02
GO:0016358	dendrite development	*HDAC2, TRAPPC4, LST1, FLRT1, PPP1R9B*	5 / 28	3.4	5.88E-02
GO:0098789	pre-mRNA cleavage required for polyadenylation	*NCBP1, CSTF2, CSTF2T*	3 / 8	7.1	6.38E-02
GO:0017183	peptidyl-diphthamide biosynthetic process from peptidyl-histidine	*DPH2, DPH6, EEF2*	3 / 8	7.1	6.38E-02
GO:0040008	regulation of growth	*SOCS1, ARMC10, CREG1, ACTL6A, RUVBL1, SOCS5, TSPYL5*	7 / 53	2.5	6.09E-02
GO:0006814	sodium ion transport	*SLC22A4, SLC4A8, SLC22A5, ATP1A4, SCN7A, SLC38A6, SLC38A8, SLC38A4, SLC9B2*	9 / 81	2.1	6.54E-02
GO:0043066	negative regulation of apoptotic process	*HDAC2, PRKAA2, XIAP, AIF1, HSP90B1, MYDGF, EDNRB, SIX4, FIGNL1, BNIP2, CTSH, AAMDC, TP63, CD74, PCID2, HSPA5, SPHK2, ANXA4, VEGFB, SOD2, YWHAZ, ZNF16, DNAJA1, GCLC, RPS6KB1, PDCD4, PPIF, NAA15, ANGPTL4, ATF5, LACRT, SQSTM1, CARD14*	33 / 455	1.4	6.61E-02
GO:0060037	pharyngeal system development	*RIPPLY3, SIX4, PLXNA2, TGFBR1*	4 / 18	4.2	6.72E-02
GO:0045022	early endosome to late endosome transport	*EEA1, KIF16B, RILP, RAB7A*	4 / 18	4.2	6.72E-02
GO:0006414	translational elongation	*EEF1G, ABTB1, EEF1E1, EEF2*	4 / 18	4.2	6.72E-02
GO:0042127	regulation of cell proliferation	*RBFOX2, CDCA7, XIAP, PDS5B, NDRG1, PPP1R9B, GNL3, CNN2, PURA, EID2, RPA3, MZB1, TIMELESS, AGTR1, ABL2, TNFRSF25*	16 / 185	1.6	6.82E-02
GO:0008543	fibroblast growth factor receptor signaling pathway	*RBFOX2, NCBP1, KIF16B, FLRT1, ANOS1, PTPN11, GTF2F1, HNRNPA1, GTF2F2*	9 / 82	2.1	6.92E-02
GO:0006260	DNA replication	*ACHE, SUPT16H, SET, PCNA, ORC5, POLA1, DNAJC2, RPA3, TIMELESS, NT5M, MCM6, DTD1, ATR, RHNO1*	14 / 155	1.7	6.93E-02
GO:0007067	mitotic nuclear division	*DCLRE1A, ANAPC13, NEK4, ZWILCH, DYNLT3, CETN3, NOLC1, KIF22, SMC3, PMF1, HAUS2, VCPIP1, STAG1, PTTG1, ZW10, TPR, TADA3, RUVBL1, TIMELESS, TXNL4A*	20 / 248	1.5	7.06E-02
GO:0000077	DNA damage checkpoint	*PTPN11, INTS7, CLOCK, ATR, RHNO1*	5 / 30	3.1	7.24E-02
GO:0001938	positive regulation of endothelial cell proliferation	*MYDGF, NRP2, NF1, VEGFB, AGGF1, CYBA, CCR3, TGFBR1*	8 / 69	2.2	7.29E-02
GO:0006749	glutathione metabolic process	*EEF1G, GGT5, GSTO2, GSTT1, EEF1E1, SOD2, CLIC1*	7 / 56	2.4	7.56E-02
GO:0043966	histone H3 acetylation	*TAF12, YEATS2, TADA3, TADA1, ELP4, TAF5L*	6 / 43	2.6	7.62E-02
GO:0014823	response to activity	*GCLC, UQCRC1, CYBA, SOD2, GCLM, SLC25A25*	6 / 43	2.6	7.62E-02

Supplementary table 6. Gene ontology analysis of differentially expressed genes: OB (0w) vs OB (12w). The GO-terms colored in green are depicted in the go plot in suppl.fig.5b. Gene names given in italics.

GO-term	Pathway	Gene symbol	Number of genes / total number of genes in pathway	Fold enrichment	P-value
GO:0006614	SRP-dependent cotranslational protein targeting to membrane	*RPL41, RPL32, RPL10, RPL23, RPL9, RPS25, RPS28, RPL24, RPL38, RPL37, RPL28, RPL26L1, RPS23, RPL19*	14 / 94	5.6	1.06E-06
GO:0019083	viral transcription	*RPL41, RPL32, RPL10, RPL23, RPL9, RPS25, RPS28, RPL24, RPL38, RPL37, RPL28, RPL26L1, RPS23, RPL19*	14 / 112	4.7	7.78E-06
GO:0000184	uclear-transcribed mRNA catabolic process, nonsense-mediated deca	*RPL41, RPL32, RPL10, RPL23, RPL9, RPS25, RPS28, RPL24, RPL38, RPL37, RPL28, RPL26L1, RPS23, RPL19*	14 / 119	4.5	1.51E-05
GO:0006413	translational initiation	*RPL41, RPL32, RPL10, RPL23, RPL9, RPS25, RPS28, EIF4E1B, RPL24, RPL38, RPL37, RPL28, RPL26L1, RPS23, RPL19*	15 / 137	4.2	1.55E-05
GO:0006364	rRNA processing	*RPL41, RPL32, RPL10, RPL23, ISG20L2, RPL9, WDR55, RPS25, RPS28, RPL24, RPL38, RPL37, RPL28, RPL26L1, RPS23, DIS3L2, RPL19*	17 / 214	3.0	1.80E-04
GO:0006334	nucleosome assembly	*HIST1H2BN, HIST1H2BM, HIST2H4B, HIST1H2BJ, HIST1H2BI, HIST1H2BF, HIST1H1D, HIST1H1E, HIST1H4D, HIST1H2BD, HIST2H2BE, HIST1H1C*	12 / 119	3.8	3.05E-04
GO:0006342	chromatin silencing	*HIST1H2AM, HIST2H2AA4, HIST3H2A, HIST1H2AE, HIST2H2AB, UBR2, HIST2H2AC*	7 / 45	5.9	1.10E-03
GO:0006412	translation	*RPL41, RPL32, RPL10, RPL23, RPL9, RPS25, SLC25A18, RPS28, RPL24, RPL38, RPL37, SLC25A45, SLC25A44, RPL28, RPL26L1, RPS23, RPL19*	17 / 253	2.5	1.13E-03
GO:0071480	cellular response to gamma radiation	*XRCC5, NOX4, ATM, CYBA, BCL2L1*	5 / 21	9.0	1.99E-03
GO:0002227	innate immune response in mucosa	*HIST1H2BJ, HIST1H2BI, HIST1H2BF, HIST1H2BD, HIST2H2BE*	5 / 25	7.6	3.86E-03
GO:0055085	transmembrane transport	*ABCC4, ABCA2, SLC12A1, ABCB8, ABCB9, ABCC10, TAPBP, TMEM245, SLC25A18, SLC37A4, SLC25A45, GPR155, SLC25A44, SLC17A3, ABCG1*	15 / 244	2.3	5.37E-03
GO:0019731	antibacterial humoral response	*HIST1H2BJ, HIST1H2BI, DEFA5, HIST1H2BF, HIST1H2BD, HIST2H2BE*	6 / 44	5.2	5.76E-03
GO:0005977	glycogen metabolic process	*PPP1CB, PPP1R3C, PHKB, SLC37A4, STK40*	5 / 29	6.5	6.68E-03
GO:2000379	positive regulation of reactive oxygen species metabolic process	*AKR1C3, AGTR1, NOX4, NOX5, TP53*	5 / 30	6.3	7.55E-03
GO:0098532	histone H3-K27 trimethylation	*HIST1H1D, HIST1H1E, HIST1H1C*	3 / 6	19.0	9.67E-03
GO:0045672	positive regulation of osteoclast differentiation	*CREB1, CSF1, CA2, PPARGC1B*	4 / 19	8.0	1.28E-02
GO:0016584	nucleosome positioning	*HIST1H1D, HIST1H1E, HIST1H1C*	3 / 8	14.2	1.74E-02
GO:0048013	ephrin receptor signaling pathway	*APH1A, EFNA1, CDC42, ACTR2, SDC2, NCK2, ARPC4*	7 / 86	3.1	2.59E-02
GO:0046902	regulation of mitochondrial membrane permeability	*BAD, TP53, BCL2L1*	3 / 10	11.4	2.70E-02
GO:0046135	pyrimidine nucleoside catabolic process	*NT5C3A, DPYD, NT5M*	3 / 11	10.3	3.25E-02
GO:0033628	regulation of cell adhesion mediated by integrin	*EFNA1, TESC, ITGB1BP1*	3 / 11	10.3	3.25E-02
GO:0060315	egative regulation of ryanodine-sensitive calcium-release channel activi	*FKBP1A, CALM3, CLIC2*	3 / 12	9.5	3.83E-02
GO:0015992	proton transport	*ATP6V0B, ATP6AP1, SLC2A13, NOX5, ATP6V0C*	5 / 49	3.9	3.96E-02
GO:0045780	positive regulation of bone resorption	*ATP6AP1, CA2, PPARGC1B*	3 / 13	8.7	4.45E-02
GO:0034097	response to cytokine	*GNAO1, JUND, AVPR2, LIFR, BCL2L1*	5 / 52	3.6	4.76E-02
GO:0042554	superoxide anion generation	*NOX4, NOX5, CYBA*	3 / 14	8.1	5.10E-02
GO:0019050	suppression by virus of host apoptotic process	*BAD, BCL2L1*	2 / 2	37.9	5.20E-02
GO:0010872	regulation of cholesterol esterification	*LAMTOR1, ABCG1*	2 / 2	37.9	5.20E-02
GO:0008286	insulin receptor signaling pathway	*BAIAP2L2, ATP6V0B, ATP6AP1, AP3S1, PIK3R1, ATP6V0C*	6 / 78	2.9	5.47E-02
GO:0030036	actin cytoskeleton organization	*CDC42, DIAPH1, PDPK1, EPB41, RAC2, PIP5K1C, FGD1, PDLIM7*	8 / 130	2.3	5.62E-02
GO:0006468	protein phosphorylation	*NEK8, MAP4K2, PDPK1, STRADB, IGFBP3, PHKB, STK19, PIK3R1, MAPK10, CREB1, PHKG1, TAOK3, ATM, MAP3K6, TRIB1, CSNK1G2, STK40, MARK2, MAPK3*	19 / 456	1.6	5.71E-02
GO:0080182	histone H3-K4 trimethylation	*HIST1H1D, HIST1H1E, HIST1H1C*	3 / 15	7.6	5.79E-02
GO:0051592	response to calcium ion	*HOMER1, GDI1, BAD, PCDH15, CALM3*	5 / 58	3.3	6.62E-02
GO:0055114	oxidation-reduction process	*BCKDHA, ABCC4, KDM2A, GDI1, MAOA, GPX6, CYP4F2, AKR1C3, JMJD1C, CYBA, CYB561A3, AKR1C4, CYP17A1, PRDX2, LDHA, NDOR1, SCO2, DPYD, UQCRC1, NOX4, NOX5, ND1, HIF1AN*	23 / 592	1.5	6.63E-02
GO:0007188	enylate cyclase-modulating G-protein coupled receptor signaling pathw	*GNAO1, AVPR2, GLP2R, CCR3*	4 / 37	4.1	7.29E-02
GO:0016311	dephosphorylation	*NT5C3A, PPM1A, PPIP5K2, NT5M, DUSP15, LPIN3*	6 / 86	2.6	7.64E-02
GO:0060154	cellular process regulating host cell cycle in response to virus	*BAD, BCL2L1*	2 / 3	25.3	7.69E-02
GO:0070315	G1 to G0 transition involved in cell differentiation	*SLC39A5, ZNF503*	2 / 3	25.3	7.69E-02
GO:0006810	transport	*ABCA2, PITPNB, GABRA4, SLC12A1, TRPV2, ABCB8, LYVE1, PITPNM1, ASNA1, CLIC2, SLC37A4, SLC25A45, ABCG1, RAB11FIP4, CNGB1*	15 / 348	1.6	7.71E-02
GO:0030225	macrophage differentiation	*CDC42, CSF1, EIF2AK1*	3 / 18	6.3	8.02E-02
GO:0000302	response to reactive oxygen species	*PRDX2, NOX4, NOX5, CYBA*	4 / 39	3.9	8.26E-02
GO:0006303	double-strand break repair via nonhomologous end joining	*KDM2A, HIST2H4B, XRCC5, ATM, HIST1H4D*	5 / 63	3.0	8.41E-02
GO:0042632	cholesterol homeostasis	*EHD1, ABCA2, LAMTOR1, SLC37A4, ABCG1*	5 / 64	3.0	8.79E-02
GO:0051493	regulation of cytoskeleton organization	*NEXN, MARK2, MAPK3*	3 / 19	6.0	8.81E-02
GO:0046330	positive regulation of JNK cascade	*CDC42, MAP4K2, TAOK3, AXIN1, DUSP15*	5 / 65	2.9	9.18E-02
GO:0070588	calcium ion transmembrane transport	*FKBP1A, TRPC4, TRPV2, CACNA2D2, OPRM1, TRPM5, TRPM3*	7 / 119	2.2	9.44E-02
GO:0009887	organ morphogenesis	*GAMT, NEK8, TLE2, CDX1, COL9A1, FGD1*	6 / 92	2.5	9.54E-02

Supplementary table 7. Gene ontology analysis of differentially expressed genes: OB (0w) vs OB (24w)**.** Gene names given in italics.

GO-term	Pathway	Gene symbol	Number of genes / total number of genes in pathway	Fold enrichment	P-value
GO:0006270	DNA replication initiation	*POLA1, MCM7, CCNE2, MCM3, MCM4, MCM6, MCM2*	7 / 32	6.9	4.24E-04
GO:0006268	DNA unwinding involved in DNA replication	*MCM7, MCM4, MCM6, MCM2*	4 / 10	12.7	3.14E-03
GO:0007399	nervous system development	*CHRM3, BEX1, GDA, ST8SIA2, KIAA2022, KALRN, PCDHA11, TTLL7, ATXN3, DLG3, NAGLU, POFUT1, ENC1, SPOCK1, EP300, PCDHB4, NRTN, PCDH1, MEF2D*	19 / 287	2.1	4.35E-03
GO:0000082	G1/S transition of mitotic cell cycle	*TRIM71, POLA1, USP37, MCM7, CCNE2, EIF4EBP1, MCM3, MCM4, MCM6, MCM2*	10 / 102	3.1	4.83E-03
GO:0006351	transcription, DNA-templated	*HIP1, THRA, ZNF491, MED19, YEATS4, ABRA, CCAR2, ING5, KHSRP, HABP4, WDR5, ZNF568, HIF1AN, MAP3K7, PIAS4, BRD3, TSC22D4, KDM2B, RING1, PRKCB, PPHLN1, ZNF90, MAK, ZNF679, ZNF513, PADI4, ZNF555, JDP2, HMX2, ASF1B, ZNF232, KANK2, KANK1, KHDRBS1, ZBTB42, ZNF22, ZNF7, HDAC7, TCEAL7, ATXN3, TCEAL6, TCEAL5, ZNF876P, CUX1, TCEAL3, TCEAL2, POLR2C, ERBB2, TCEAL1, NOTO, ZNF229, ZNF226, PATZ1, ZNF621, ZBTB7A, ZNF785, MEF2D, ZSCAN18, ZNF420, HEXIM2, CBX6, TCF7L1, SETD1B, ZNF180, YLPM1, SMAD9, NR1D2, MINA, VAX1, ZNF33B, ZBTB6, ZZZ3, ZFP62, NFIC, AGO2, ZNF416, ZNF613, ESF1, POLR3H, INSM1, ZNF775, ZNF257*	82 / 1955	1.3	5.56E-03
GO:0006260	DNA replication	*ING5, POLA1, MCM7, LIG1, NFIC, MCM3, MCM4, LIG3, SPHAR, MCM6, DTD1, MCM2*	12 / 155	2.5	9.94E-03
GO:0007169	transmembrane receptor protein tyrosine kinase signaling pathway	*DDR1, NTRK3, ERBB2, CD7, ABL2, NRTN, MET, FRK, MTSS1*	9 / 96	3.0	1.07E-02
GO:0046627	negative regulation of insulin receptor signaling pathway	*SOCS2, KANK1, SOCS1, PRKCB, TSC1*	5 / 29	5.5	1.23E-02
GO:0007568	aging	*SRR, KRTAP4-9, KRTAP4-8, OGG1, ITGB2, ATP6, CAT, FBXO4, DMD, EEF2, FOXO3, SNCA*	12 / 165	2.3	1.53E-02
GO:0051593	response to folic acid	*OGG1, MTHFR, EEF2*	3 / 7	13.6	1.87E-02
GO:0000122	negative regulation of transcription from RNA polymerase II promoter	*KANK2, TCERG1, NEDD4L, ZBTB42, FOXO3, RBPJ, GLI3, STRN3, HDAC7, CUX1, UBC, TCEAL1, EP300, HOXA7, FEZF2, ZBTB7A, SNCA, PIAS4, WWTR1, HEXIM2, CBX6, KDM2B, FST, HFE2, MINA, VAX1, EFNA1, YEATS2, NFIC, ZNF613, TCF4, INSM1, JDP2, FRK*	34 / 720	1.5	1.97E-02
GO:0009083	branched-chain amino acid catabolic process	*MCCC1, PPM1K, ACADSB, BCAT2*	4 / 19	6.7	2.06E-02
GO:0006355	regulation of transcription, DNA-templated	*HIP1, THRA, ZNF491, OGG1, YEATS4, CCAR2, POFUT1, KHSRP, HABP4, EP300, ZNF568, PKNOX1, EMX1, PPHLN1, ZNF90, MAK, PPIE, ZNF679, ZNF513, PADI4, ZNF555, ABCG1, HMX2, ASF1B, ZNF232, KANK1, MAGED1, SRRT, RNF38, ZNF22, ZNF7, HDAC7, PDLIM1, ATXN3, ZNF876P, NOTO, ZNF229, DMD, ZNF226, PATZ1, ZNF621, ZNF785, ZNF420, RNF20, WWTR1, TCF7L1, CDX1, SETD1B, ZNF180, YLPM1, NR1D2, GTF2H3, ZNF33B, ZBTB6, ZZZ3, YEATS2, ZFP62, ZNF416, ZNF613, ESF1, ZNF775, ZNF257*	62 / 1504	1.3	2.33E-02
GO:0007288	sperm axoneme assembly	*SPAG16, PLA2G3, TPGS1*	3 / 8	11.9	2.44E-02
GO:0006283	transcription-coupled nucleotide-excision repair	*LIG1, POLR2C, UBC, EP300, PPIE, LIG3, GTF2H3*	7 / 74	3.0	2.88E-02
GO:0043687	post-translational protein modification	*FN3KRP, MINA, ARSB, SUMF2*	4 / 22	5.8	3.05E-02
GO:0007010	cytoskeleton organization	*CECR2, FMNL1, TUBB6, HIP1, ZMYM3, PLCE1, FITM2, YEATS4, DMD, OBSL1, CAMSAP1*	11 / 161	2.2	3.11E-02
GO:0016567	protein ubiquitination	*WWTR1, FBXO18, RNF38, FBXO25, WWP1, NEDD4L, FBXL15, KLHL3, ASB11, ASB12, FBXO43, KLHL30, PJA1, SOCS2, SOCS1, RAB40C, ASB8, ENC1, FBXO4*	19 / 359	1.7	3.54E-02
GO:0001558	regulation of cell growth	*SOCS2, DDR1, KIAA1109, IGFBP3, AGTR1, SPOCK1, PLCE1*	7 / 80	2.8	4.00E-02
GO:0001933	negative regulation of protein phosphorylation	*WWTR1, NTRK3, CD109, IGFBP3, INSM1, SNCA*	6 / 61	3.1	4.25E-02
GO:0045892	negative regulation of transcription, DNA-templated	*PIAS4, SMARCE1, KHDRBS1, HEXIM2, TSC22D4, THRA, RING1, MAGED1, WWP1, NR1D2, MINA, PTPRK, RBPJ, CCAR2, GLI3, STRN3, DKK3, TCEAL7, YEATS2, BCL7A, HOXA7, FEZF2, PATZ1, ZBTB7A*	24 / 499	1.5	4.38E-02
GO:0006048	UDP-N-acetylglucosamine biosynthetic process	*AMDHD2, PGM3, RENBP*	3 / 11	8.7	4.50E-02
GO:0043966	histone H3 acetylation	*ING5, YEATS2, WDR5, ELP4, MAP3K7*	5 / 43	3.7	4.54E-02
GO:0008285	negative regulation of cell proliferation	*KANK2, DDR1, CGREF1, CDKN2B, KLF13, TESC, IGFBP3, TSC1, PTPRK, ADARB1, RBPJ, GLI3, ING5, FABP3, DLG3, FTH1, CDH13, INSM1, FEZF2, FRK*	20 / 396	1.6	4.55E-02
GO:0072661	protein targeting to plasma membrane	*TESC, BSG, ANK3, MYL12A*	4 / 26	4.9	4.70E-02
GO:0007067	mitotic nuclear division	*DCLRE1A, USP37, PLK5, ZWILCH, SMC5, YEATS4, CCSAP, KLHL42, STAG1, WEE1, STAG2, RAD21, FSD1, MAPRE2*	14 / 248	1.8	5.05E-02
GO:0051301	cell division	*DCLRE1A, USP37, PLK5, LIG1, ZWILCH, SMC5, LIG3, CCSAP, KLHL42, STAG1, WEE1, STAG2, CCNE2, RAD21, POGZ, MAP4, FSD1, MAPRE2*	18 / 350	1.6	5.13E-02
GO:0014065	phosphatidylinositol 3-kinase signaling	*ERBB2, PIK3R2, PIK3R1, NYAP2*	4 / 27	4.7	5.17E-02
GO:0035329	hippo signaling	*WWTR1, AMOTL2, AMOTL1, TJP2*	4 / 27	4.7	5.17E-02
GO:0051492	regulation of stress fiber assembly	*TSC1, PIK3R1, SYNPO*	3 / 13	7.3	6.12E-02
GO:0021592	fourth ventricle development	*KDM2B, MYH10*	2 / 2	31.7	6.19E-02
GO:1902856	negative regulation of nonmotile primary cilium assembly	*MAK, MAP4*	2 / 2	31.7	6.19E-02
GO:0051012	microtubule sliding	*SPAG16, MAP4*	2 / 2	31.7	6.19E-02
GO:0007275	multicellular organism development	*CDX1, SIAH3, EMP1, BTBD7, TPGS1, RPS4Y1, WNT16, ZNF7, DKK3, ARVCF, TEF, CUX1, ZMYM3, FES, MAK, ENC1, GNRH2, TLL2, PAEP, ZNF568, CLC, LEFTY2, ZBTB7A, ASF1B*	24 / 521	1.5	6.43E-02
GO:0006357	regulation of transcription from RNA polymerase II promoter	*SMARCE1, BRD3, TCF7L1, THRA, PRKCB, MAGED1, UCP1, ELP4, FOXO3, TCEAL7, TCEAL6, TCEAL5, TEF, CUX1, ZMYM3, TCEAL3, TCEAL2, RAD21, TAF4, JDP2, ZSCAN18*	21 / 441	1.5	6.72E-02
GO:0007625	grooming behavior	*CTNS, HPRT1, QRFP*	3 / 14	6.8	7.00E-02
GO:0031667	response to nutrient levels	*UCP1, CYBA, FOXO3, ACACB*	4 / 31	4.1	7.26E-02
GO:0030100	regulation of endocytosis	*HIP1, RAB4B, CDH13, ABL2*	4 / 31	4.1	7.26E-02
GO:0042542	response to hydrogen peroxide	*ND6, STK25, CAT, HBA2, EEF2*	5 / 51	3.1	7.59E-02
GO:0045947	negative regulation of translational initiation	*AGO2, EIF4EBP1, EIF4EBP3*	3 / 15	6.3	7.91E-02
GO:0034375	high-density lipoprotein particle remodeling	*APOM, PLTP, ABCG1*	3 / 15	6.3	7.91E-02
GO:0046037	GMP metabolic process	*DLG3, GMPR2, TJP2*	3 / 15	6.3	7.91E-02
GO:0070555	response to interleukin-1	*IRAK2, MTHFR, CYBA, SNCA*	4 / 33	3.8	8.42E-02
GO:0006468	protein phosphorylation	*MAP4K2, PLK5, DMPK, PRKCB, STRADB, IGFBP3, PRKAG1, SMAD9, PHKB, STK19, PIK3R1, KALRN, MYLK4, FES, IRAK2, STK25, MAK, ERBB2, ALPK1, PRKACA, FRK*	21 / 456	1.5	8.48E-02
GO:0007525	somatic muscle development	*NEB, PPP2R3A*	2 / 3	21.2	9.14E-02
GO:1903426	regulation of reactive oxygen species biosynthetic process	*UCP1, SNCA*	2 / 3	21.2	9.14E-02
GO:0021678	third ventricle development	*KDM2B, MYH10*	2 / 3	21.2	9.14E-02
GO:0015012	heparan sulfate proteoglycan biosynthetic process	*GLCE, NDST3, B3GALT6*	3 / 17	5.6	9.84E-02

Supplementary table 8. Gene ontology analysis of differentially expressed genes: OB (0w) vs OB (52w). The GO-terms colored in green are mentionned in the text. Gene names given in italics.

GO-term	Pathway	Gene symbol	Number of genes / total number of genes in pathway	Fold enrichment	P-value
GO:0005977	glycogen metabolic process	*PPP1R1A, PPP1R3B, PPP1R3E, PPP1R3D, PPP1R2P3, PRKAG2, PHKB, PYGL, SLC37A4, STK40*	10 / 29	5.3	6.19E-05
GO:0006468	protein phosphorylation	*TP53RK, CCNH, MAST2, PRKAG2, MYLK4, RPS6KA3, RPS6KA5, PDK4, SCYL2, PRKG1, MAP4K2, CHUK, MMD, STRADB, PRKCD, DAPK3, ERN1, FGR, BCR, MAPKAPK2, FASTKD5, SGK1, STK40, GUCY2D, PIK3R1, NLK, KALRN, AURKA, MAPK8, NUAK2, GRK4, ERBB2, STK38, STK38L, CSK, MAPK6, LYN, MAP3K2, SRPK3, CDK19, DMPK, LIMK1, SMAD9, PHKB, MERTK, BCKDK, MAPK10, PINK1, CDK7, FES, STK25, AAK1*	52 / 456	1.8	1.08E-04
GO:0034976	response to endoplasmic reticulum stress	*ERO1A, XBP1, FAM129A, TXNDC11, ATP2A1, PIK3R2, PIK3R1, PARK2, ERN1, ERP44, DDRGK1, CREB3L2, UBQLN1, NRBF2, ATF4*	15 / 75	3.1	3.01E-04
GO:0007050	cell cycle arrest	*KHDRBS1, PRNP, CDKN2B, CDC123, CAB39, CUL3, STRADB, AK1, PRKAG2, DHCR24, VASH1, MLF1, PML, UHMK1, ERN1, PPM1A, CDK7, PCBP4, RRAGB, IRF1, LAMTOR1, MAP2K6*	22 / 141	2.4	3.10E-04
GO:0006915	apoptotic process	*PLEKHF1, PPP1R13B, SH3KBP1, ITGB2, APIP, CIB1, ARRB1, LITAF, CASP14, RPS6KA3, WDR92, ADAMTSL4, RASSF5, CASP4, PEA15, CASP1, CTSC, HTATIP2, AREL1, APAF1, PRKCD, DAPK3, AXIN1, MUL1, FBXO10, ZDHHC16, TRAIP, NISCH, TRAF3, IRF1, CDIP1, MAL, CHI3L1, FAM32A, SGK1, S100A9, S100A8, BIRC3, MCM2, BEX2, FAM188A, NUAK2, RNF216, RAD21, CD14, RNF130, SMNDC1, SNCA, MAP2K6, TIGAR, BAD, STAT1, SIAH3, DHCR24, PML, ACTC1, LCN2, CIAPIN1*	58 / 567	1.6	7.05E-04
GO:0070059	sic apoptotic signaling pathway in response to endoplasmic reticulum s	*ERN1, ERO1A, XBP1, APAF1, CASP4, TMBIM6, ATP2A1, PML, ATF4*	9 / 33	4.2	1.01E-03
GO:0000209	protein polyubiquitination	*PSMD12, DTX3L, SMURF1, CUL3, UBR2, MARCH5, PARK2, PSMB9, DDB2, RNF41, PSMA5, ZNRF1, NPEPPS, PSMD7, HECTD2, PSMD2, UBR5, MKRN1, PSME4, FBXO6, UBE2V1, LONRF1, ASB2, TRIM21*	24 / 184	2.0	1.97E-03
GO:0015671	oxygen transport	*HBM, HBG2, HBG1, HBB, HBA2, HBD*	6 / 15	6.1	2.00E-03
GO:0043161	proteasome-mediated ubiquitin-dependent protein catabolic process	*PSMD12, CUL3, RNF38, ARRB1, RNF6, FBXO45, PARK2, ZNRF1, PSMD7, RNF216, KAT5, PSMD2, CDC26, TBL1X, EDEM3, SMURF1, SIAH3, AXIN1, WWP1, KCTD5, RAD23B, PML, PSMB9, PSMA5, PSME4*	25 / 203	1.9	3.34E-03
GO:0016567	protein ubiquitination	*CUL3, RNF38, ARRB1, FBXO45, ASB11, DCAF5, DTX4, ASB18, BACH1, PARK2, RNF135, VCPIP1, RAB40C, TRIM28, FBXO7, RNF130, TRIM21, DCAF12, SMURF1, BTBD18, MUL1, WWP1, LMO7, FBXO32, FBXO10, MID2, FBXO30, TRAIP, RNF168, SH3RF2, PINK1, KLHL5, NEURL2, TRAF3, NEDD4, ASB5, PDZRN4, BIRC3*	38 / 359	1.6	3.79E-03
GO:0005975	carbohydrate metabolic process	*CHID1, GUSBP11, ST8SIA1, IDH3G, SLC2A3, PYGL, BPGM, NAGPA, KIAA1161, GALC, LDHB, LDHA, FGGY, ALDH2, MAN2A2, AMDHD2, MLEC, ST3GAL5, CHI3L1, GLA, GALK2, LDHAL6A*	22 / 174	1.9	4.58E-03
GO:0031648	protein destabilization	*XBP1, CCDC88C, MUL1, TRIM21, PARK2, ISOC2, NOMO2, SNCA*	8 / 35	3.5	6.54E-03
GO:0006342	chromatin silencing	*HIST1H2AM, HIST2H2AA4, HIST3H2A, H2AFZ, HIST1H2AK, HIST1H2AE, HIST2H2AB, UBR2, HIST1H2AC*	9 / 45	3.1	7.84E-03
GO:0001867	complement activation, lectin pathway	*FCN3, KRT1, MASP2, MASP1*	4 / 7	8.8	7.89E-03
GO:0035556	intracellular signal transduction	*DDX3X, GUCY2D, CAB39, DGKA, MAST2, PSEN2, PRKAG2, ASB11, NLK, ASB18, KALRN, ADCY7, RPS6KA3, RPS6KA5, NUAK2, RASSF5, STK38, STK38L, GPR155, POLR2M, MAP4K2, MAP3K3, SMAD4, PRKCD, DAPK3, BCR, PINK1, TYROBP, NEURL2, PPP1R1A, NRG4, PPP1R1C, PRKCQ, ASB5, ASB2, SGK1, PLCD1, SIPA1, RAPGEF3, GCOM1*	40 / 403	1.5	8.57E-03
GO:0033209	tumor necrosis factor-mediated signaling pathway	*PSMD12, TNFRSF6B, STAT1, COMMD7, PSMB9, TNFSF13B, TNFRSF1A, PSMA5, PSMD7, TRAF3, PSMD2, TNFSF4, PSME4, CD27, CDIP1, BIRC3*	16 / 118	2.1	9.60E-03
GO:1903076	regulation of protein localization to plasma membrane	*VAMP8, KALRN, GBP1, VTI1B*	4 / 8	7.7	1.20E-02
GO:1903204	negative regulation of oxidative stress-induced neuron death	*PINK1, FBXO7, ATF4*	3 / 3	15.3	1.21E-02
GO:0046320	regulation of fatty acid oxidation	*FABP3, PDK4, PRKAG2*	3 / 3	15.3	1.21E-02
GO:0090141	positive regulation of mitochondrial fission	*PINK1, MUL1, DDHD1, MARCH5, PARK2*	5 / 15	5.1	1.37E-02
GO:0010508	positive regulation of autophagy	*XBP1, PLEKHF1, BAD, TP53INP2, PIK3CB, TRIM21, MID2, MTDH*	8 / 40	3.1	1.37E-02
GO:0051865	protein autoubiquitination	*TRIM71, UBE2T, RNF8, TRIM21, MARCH5, RNF141, PARK2, RNF41, DDB2*	9 / 50	2.8	1.47E-02
GO:0031647	regulation of protein stability	*KHDRBS1, DPM2, XBP1, RNF128, SIAH3, USP3, MUL1, QRSL1, FBXO7, PARK2, AURKA*	11 / 70	2.4	1.47E-02
GO:0060333	interferon-gamma-mediated signaling pathway	*STAT1, IFNGR2, IRF1, PRKCD, IRF2, HLA-C, NMI, IFI30, TRIM21, GBP1, PML*	11 / 71	2.4	1.62E-02
GO:0006853	carnitine shuttle	*CPT1A, PRKAG2, SLC25A20, ACACB*	4 / 9	6.8	1.72E-02
GO:0090244	Wnt signaling pathway involved in somitogenesis	*RGS19, AXIN1, PPP2R3A, LRP6*	4 / 9	6.8	1.72E-02
GO:0071353	cellular response to interleukin-4	*ALAD, XBP1, TUBA1B, CORO1A, PML, MCM2*	6 / 24	3.8	1.74E-02
GO:0031623	receptor internalization	*CALCRL, CXCR1, GRK4, NEDD4, ITGB2, CLTC, CD9, SNCA*	8 / 43	2.9	2.00E-02
GO:0045727	positive regulation of translation	*UCN, PINK1, DDX3X, SERP1, DDX39B, ERBB2, CIRBP, FAM129A, RBMS3*	9 / 53	2.6	2.04E-02
GO:0042997	negative regulation of Golgi to plasma membrane protein transport	*LYPLA1, PKDCC, CSK*	3 / 4	11.5	2.33E-02
GO:0001771	immunological synapse formation	*DOCK8, NCK2, DOCK2, CORO1A*	4 / 10	6.1	2.34E-02
GO:0009615	response to virus	*IFNAR2, IFITM2, DDX3X, BTBD17, CHUK, IFNGR2, ODC1, ZC3HAV1, IFIT3, IFIT2, IFIH1, FGR, TNFSF4, LCN2*	14 / 110	2.0	2.64E-02
GO:0006979	response to oxidative stress	*UCN, PRNP, ABCC2, SRXN1, TAT, KRT1, DHCR24, PARK2, PDLIM1, ALAD, PINK1, ALS2, STK25, HAO1*	14 / 110	2.0	2.64E-02
GO:0010800	positive regulation of peptidyl-threonine phosphorylation	*CAB39, EGF, AXIN1, CHI3L1, PRKAG2, CALM1*	6 / 27	3.4	2.82E-02
GO:0014065	phosphatidylinositol 3-kinase signaling	*XBP1, ERBB2, PIK3R2, PIK3CB, PIK3R1, NYAP2*	6 / 27	3.4	2.82E-02
GO:0070534	protein K63-linked ubiquitination	*RNF168, UBE2T, NEDD4, RNF8, UBE2O, UBE2V1, PARK2*	7 / 37	2.9	3.08E-02
GO:0046777	protein autophosphorylation	*LYN, DDR1, MAP3K3, MYLK2, DAPK3, PRKX, NLK, AURKA, UHMK1, ERN1, BCR, FGR, TRIM28, FES, STK25, MAPKAPK2, ERBB2, AAK1, CSK*	19 / 172	1.7	3.14E-02
GO:0006357	regulation of transcription from RNA polymerase II promoter	*ZNF395, ENY2, TSHZ3, THRA, MAGED1, CITED4, FOXK2, UBP1, ARID4B, CHD3, TRAK1, LITAF, TCEAL6, DBP, TCEAL5, ZMYM3, TCEAL3, ZNF704, RAD21, TBL1Y, ZNF446, GTF2IRD2B, ZNF641, PKNOX2, HTATIP2, ZSCAN18, IFNAR2, KDM4C, ZSCAN5B, ARID5A, ELP2, SNF8, FOXP4, FOXN1, TFDP1, TFDP2, LPIN1, NFKBID, ATF4, VGLL3*	40 / 441	1.4	3.19E-02
GO:0035666	TRIF-dependent toll-like receptor signaling pathway	*CHUK, TRAF3, LY96, CD14, TANK, BIRC3*	6 / 28	3.3	3.25E-02
GO:0007528	neuromuscular junction development	*CHRNA1, ALS2, NEDD4, ERBB2, CACNA2D2, COL4A5*	6 / 28	3.3	3.25E-02
GO:0006513	protein monoubiquitination	*UBE2T, CUL3, NEDD4, UBE2O, TRIM21, PARK2*	6 / 28	3.3	3.25E-02
GO:0070373	negative regulation of ERK1 and ERK2 cascade	*LYN, ERRFI1, XBP1, SMAD4, DUSP3, CSK, ARRB1, VRK3, GBP1*	9 / 58	2.4	3.33E-02
GO:0036498	IRE1-mediated unfolded protein response	*ERN1, DNAJC3, SEC61A1, XBP1, SERP1, CXXC1, SRPRB, HDGF, EXTL3*	9 / 59	2.3	3.64E-02
GO:0071407	cellular response to organic cyclic compound	*RAP1B, P2RY6, SLC16A1, RGS19, STAT1, P2RY1, IL18, AXIN1, SMAD9*	9 / 59	2.3	3.64E-02
GO:0043456	regulation of pentose-phosphate shunt	*TIGAR, PGAM2, BPGM*	3 / 5	9.2	3.71E-02
GO:1900103	positive regulation of endoplasmic reticulum unfolded protein response	*ERN1, XBP1, PIK3R1*	3 / 5	9.2	3.71E-02
GO:0010637	negative regulation of mitochondrial fusion	*MUL1, OMA1, PARK2*	3 / 5	9.2	3.71E-02
GO:0015833	peptide transport	*TAP1, ABCB9, DISP1*	3 / 5	9.2	3.71E-02
GO:0010991	negative regulation of SMAD protein complex assembly	*PPM1A, PMEPA1, LDLRAD4*	3 / 5	9.2	3.71E-02
GO:0051583	dopamine uptake involved in synaptic transmission	*SLC6A2, PARK2, SNCA*	3 / 5	9.2	3.71E-02
GO:0044314	protein K27-linked ubiquitination	*UBE2T, RNF6, PARK2*	3 / 5	9.2	3.71E-02
GO:0001887	selenium compound metabolic process	*TXNRD1, SCLY, SEPHS2*	3 / 5	9.2	3.71E-02
GO:0006089	lactate metabolic process	*LDHB, LDHA, SLC37A4*	3 / 5	9.2	3.71E-02
GO:1901985	positive regulation of protein acetylation	*XBP1, KAT5, RAPGEF3*	3 / 5	9.2	3.71E-02
GO:0045746	negative regulation of Notch signaling pathway	*MEG3, DLX1, HEY1, BCL6, NUMB, DLK1*	6 / 29	3.2	3.73E-02
GO:0042981	regulation of apoptotic process	*BNIP3L, BEX2, PPP1R13B, MSTN, APAF1, STAT1, MAGED1, DAPK3, FBXO10, ETS1, TNFRSF1A, BCL6, TRAF3, RASSF5, CASP4, PEA15, CASP1, CARD16, SGK1, BIRC3, ZNF420, BCL2L1*	22 / 213	1.6	3.77E-02
GO:0014070	response to organic cyclic compound	*LYN, ABCC4, CPT1A, ND1, PLIN2, SLC37A4, ACACB, ACAT1*	8 / 49	2.5	3.81E-02
GO:0060394	negative regulation of pathway-restricted SMAD protein phosphorylatio	*XBP1, PMEPA1, LDLRAD4, ENG*	4 / 12	5.1	3.89E-02
GO:0055008	cardiac muscle tissue morphogenesis	*EGLN1, MYLK2, ACTC1, TTN*	4 / 12	5.1	3.89E-02
GO:0042787	ein ubiquitination involved in ubiquitin-dependent protein catabolic proc	*AREL1, SMURF1, SIAH3, CUL3, WWP1, FBXO45, FBXO10, PARK2, AURKA, HERC4, RNF128, HECTD2, CDC26, NEDD4, UBR5, ASB2, FBXO7*	17 / 153	1.7	4.09E-02
GO:0010506	regulation of autophagy	*XBP1, RRAGB, DAPK3, CASP1, PIP4K2A, PIK3R2, NRBF2, PARK2*	8 / 50	2.5	4.19E-02
GO:0002474	antigen processing and presentation of peptide antigen via MHC class	*SEC24A, SAR1B, TAP2, HLA-C, TAP1, ABCB9*	6 / 30	3.1	4.24E-02
GO:0050729	positive regulation of inflammatory response	*CDK19, TNFSF4, IL18, LPL, S100A12, ETS1, S100A9, LDLR, S100A8, TNFRSF1A*	10 / 73	2.1	4.67E-02
GO:0045765	regulation of angiogenesis	*EGLN1, WARS, ERBB2, KRT1, ETS1, HTATIP2*	6 / 31	3.0	4.80E-02
GO:0019752	carboxylic acid metabolic process	*LDHB, LDHA, MIF, LDHAL6A*	4 / 13	4.7	4.81E-02
GO:0045088	regulation of innate immune response	*RNF135, FGR, IRF1, BIRC3*	4 / 13	4.7	4.81E-02
GO:0001817	regulation of cytokine production	*LYN, TRAF3, CSK, LITAF*	4 / 13	4.7	4.81E-02
GO:0055114	oxidation-reduction process	*ERO1A, ALDH1L1, MTRR, SRXN1, NDUFA10, TXNDC12, IFI30, CYB561D2, SH3PXD2A, FTH1, FDXR, QSOX1, DHRS4L2, LBR, HTATIP2, ABCC4, SORD, HSD11B1L, CYB561A3, CYP27A1, POR, ASPH, SCO2, ALDH1A2, KIAA1456, GFOD2, ND1, GMPR2, HSD17B14, HSD17B8, FOXRED1, LDHB, LDHA, HSD17B1, SNCA, CYP2J2, EGLN1, HSD3B7, KDM4C, RNLS, TXNRD1, TXNL1, GSR, AKR1C3, AKR1C2, DHCR24, DHRS7, NDUFAF5, ALDH8A1, LDHAL6A*	50 / 592	1.3	4.92E-02
GO:0045087	innate immune response	*DDX3X, NLRC5, LY96, IL27, ZC3HAV1, CORO1A, IFIH1, RNF135, TRIM28, CLEC7A, CASP4, S100A12, CSK, CD14, TRIM21, CHID1, LYN, MAP4K2, APOBEC3G, CHUK, SH2D1B, HLA-C, MIF, MID2, PML, FGR, IGLL5, TYROBP, FES, ELF4, TRAF3, LCN2, CD300E, PADI4, CD46, S100A9, LGR4, S100A8*	38 / 430	1.4	4.97E-02

GO:0006511	ubiquitin-dependent protein catabolic process	*USP48, XBP1, USP38, USP6, CUL3, USP3, USP11, USP12, RNF8, RNF6, UCHL3, FBXO32, UCHL5, RNF168, PINK1, NPLOC4, TP53INP2, UBR5, FBXO7*	19 / 182	1.6	5.02E-02
GO:0042472	inner ear morphogenesis	*BCR, PRRX1, INSIG1, CHD7, FOXI1, OTX1, SOBP, HMX2*	8 / 52	2.4	5.03E-02
GO:0050727	regulation of inflammatory response	*LYN, IL20, BCL6, TNFSF4, CASP4, CASP1, NR1D2, PIK3AP1, BIRC3*	9 / 63	2.2	5.08E-02
GO:0090002	establishment of protein localization to plasma membrane	*TSPAN14, EFR3A, JUP, TSPAN5, EFR3B, FAM126A, TNFRSF1A*	7 / 42	2.6	5.30E-02
GO:0002489	presentation of endogenous peptide antigen via MHC class Ib via ER p	*TAP2, TAP1, ABCB9*	3 / 6	7.7	5.33E-02
GO:0032237	activation of store-operated calcium channel activity	*STIM1, ASPH, TMEM110*	3 / 6	7.7	5.33E-02
GO:0002485	presentation of endogenous peptide antigen via MHC class I via ER pa	*TAP2, TAP1, ABCB9*	3 / 6	7.7	5.33E-02
GO:0002591	ulation of antigen processing and presentation of peptide antigen via M	*TAP2, TAP1, ABCB9*	3 / 6	7.7	5.33E-02
GO:0042632	cholesterol homeostasis	*EHD1, XBP1, FABP3, IL18, LPL, LAMTOR1, SLC37A4, LDLR, ABCG1*	9 / 64	2.2	5.49E-02
GO:0030512	tive regulation of transforming growth factor beta receptor signaling pat	*PPM1A, XBP1, CHST11, SMURF1, HTRA4, PMEPA1, LDLRAD4, MTMR4, ENG*	9 / 64	2.2	5.49E-02
GO:0008299	isoprenoid biosynthetic process	*IDI1, COQ2, IDI2, ISPD*	4 / 14	4.4	5.84E-02
GO:0001678	cellular glucose homeostasis	*HKDC1, PIK3R2, CARTPT, PIK3R1*	4 / 14	4.4	5.84E-02
GO:0018105	peptidyl-serine phosphorylation	*DMPK, CAB39, MAST2, PRKCD, PRKX, VRK3, UHMK1, PINK1, MAPK8, STK38, MAPKAPK2, STK38L, PRKCQ, SGK1*	14 / 125	1.7	6.32E-02
GO:0050790	regulation of catalytic activity	*DPM2, DMPK, BAG2, PPP1R3B, RCAN2, PRKAG2, PPP2R3A, SGK1, TTN*	9 / 66	2.1	6.37E-02
GO:0090090	negative regulation of canonical Wnt signaling pathway	*TLE2, PSMD12, RGS19, CUL3, AXIN1, PRICKLE1, PPP2R3A, MLLT3, PARK2, PSMB9, LRP6, PSMA5, PSMD7, PSMD2, PSME4, LZTS2, SCYL2*	17 / 163	1.6	6.56E-02
GO:0006412	translation	*COA1, WARS, DHPS, MRPL27, RPL36A, MRPL34, GTF2H3, MRPS6, SLC25A37, COPS5, SLC25A39, RPL18A, SLC25A19, UCP3, AGO2, SLC25A10, RPS2, SLC25A20, RPS11, CPEB2, SLC25A44, RPL28, FARSB, RPL19*	24 / 253	1.5	6.58E-02
GO:0006006	glucose metabolic process	*NISCH, CPT1A, SERP1, SORD, PDK4, OMA1, BPGM, SLC37A4, PGM2L1*	9 / 67	2.1	6.84E-02
GO:0042993	positive regulation of transcription factor import into nucleus	*XBP1, TRIM28, PIK3R2, PIK3R1*	4 / 15	4.1	6.96E-02
GO:0045948	positive regulation of translational initiation	*KHDRBS1, CDC123, DDX3X, UHMK1*	4 / 15	4.1	6.96E-02
GO:1904707	positive regulation of vascular smooth muscle cell proliferation	*ERN1, XBP1, DDX39B, MMP9*	4 / 15	4.1	6.96E-02
GO:0001964	startle response	*UCN, PCDH15, PARK2, GRIN2D*	4 / 15	4.1	6.96E-02
GO:0030968	endoplasmic reticulum unfolded protein response	*ERO1A, EDEM3, XBP1, SERP1, CREB3L2, EDEM2, AARS*	7 / 45	2.4	7.00E-02
GO:0014894	response to denervation involved in regulation of muscle adaptation	*CASQ1, MTMR4, FBXO32*	3 / 7	6.6	7.14E-02
GO:1903077	negative regulation of protein localization to plasma membrane	*CLTC, NUMB, GBP1*	3 / 7	6.6	7.14E-02
GO:0070233	negative regulation of T cell apoptotic process	*DOCK8, CD27, PRKCQ*	3 / 7	6.6	7.14E-02
GO:0006032	chitin catabolic process	*CHID1, CHI3L1, CTBS*	3 / 7	6.6	7.14E-02
GO:0010890	positive regulation of sequestering of triglyceride	*LPL, OSBPL11, PLIN5*	3 / 7	6.6	7.14E-02
GO:0070050	neuron cellular homeostasis	*CHRNA1, FGGY, PARK2*	3 / 7	6.6	7.14E-02
GO:0002481	sing and presentation of exogenous protein antigen via MHC class Ib, T	*TAP2, TAP1, ABCB9*	3 / 7	6.6	7.14E-02
GO:0046689	response to mercury ion	*ALAD, TAT, PGAM2*	3 / 7	6.6	7.14E-02
GO:0007049	cell cycle	*LIN54, BEX2, CDC123, LIN37, PRKCD, CHTF18, USP39, AURKA, PTP4A1, RPS6KA3, CCPG1, PPP1R1C, CINP, CDK2AP1, FAM32A, E2F3, MAPK6, EVI5, TRIM21, MCM2, AVPI1*	21 / 217	1.5	7.34E-02
GO:0032922	circadian regulation of gene expression	*PER1, MAGED1, CRY2, CARTPT, LGR4, PML, ATF4, CIART*	8 / 57	2.2	7.55E-02
GO:2001235	positive regulation of apoptotic signaling pathway	*ING5, APAF1, MAGED1, PRKCD, CTSC*	5 / 25	3.1	7.58E-02
GO:0002223	stimulatory C-type lectin receptor signaling pathway	*LYN, PSMA5, PSMD12, PSMD7, RPS6KA5, CHUK, CLEC7A, PSMD2, PRKCD, PSME4, UBE2V1, PSMB9*	12 / 105	1.8	7.95E-02
GO:0051592	response to calcium ion	*HOMER1, BAD, NEDD4, KCNMA1, FGG, PCDH15, CALM1, TTN*	8 / 58	2.1	8.13E-02
GO:0035269	protein O-linked mannosylation	*DPM2, B4GAT1, ISPD, POMGNT2*	4 / 16	3.8	8.16E-02
GO:1902187	negative regulation of viral release from host cell	*TRIM28, TRIM21, MID2, PML*	4 / 16	3.8	8.16E-02
GO:0051642	centrosome localization	*NIN, ASUN, SYNE2, AURKA*	4 / 16	3.8	8.16E-02
GO:0032418	lysosome localization	*BLOC1S1, BLOC1S2, HPS6, LAMTOR1*	4 / 16	3.8	8.16E-02
GO:0009409	response to cold	*THRA, UCP3, CIRBP, LPL, HSPA2, METRNL*	6 / 36	2.6	8.19E-02
GO:0098869	cellular oxidant detoxification	*GSTZ1, SRXN1, TXNRD1, SESN2, GSR, TXNL1, HBB, HBA2, S100A9*	9 / 70	2.0	8.39E-02
GO:0045944	positive regulation of transcription from RNA polymerase II promoter	*UCN, DDX3X, THRA, CCNH, CITED4, CHD7, SLC40A1, FOXI1, MED19, SCX, ARRB1, ARID4B, BACH1, ETS1, PARK2, RPS6KA3, RPS6KA5, DBP, KAT5, HEY1, TRIM28, CREB3L2, KPNA6, NCK2, CCNL2, HOXA7, FEZF2, CHUK, H2AFZ, NCOA3, ZBTB38, IL18, RFX3, TNFRSF1A, NPAS4, MED25, TFDP1, TFDP2, ELF4, TFR2, IRF1, RFX4, IRF2, MET, ATF4, ENG, BEX1, NLRC5, FOXK2, GATA5, PIK3R2, PIK3R1, LRP6, IFT74, NHLH2, RAD21, P2RY1, OTX1, TBL1X, EGLN1, CDKN2B, XBP1, SMAD4, PRRX1, CDX1, STAT1, ESRRG, GRHL2, PML, PER1, CDK7, COPS5, CCPG1, AGO2, ZNF613, LPIN1*	76 / 981	1.2	8.43E-02
GO:0046854	phosphatidylinositol phosphorylation	*EFR3A, NRG4, IMPA2, EGF, ERBB2, PIP4K2A, PIK3R2, PIK3CB, PIK3R1, EFR3B, FAM126A*	11 / 94	1.8	8.47E-02
GO:0071260	cellular response to mechanical stimulus	*MAP3K2, MAG, MAPK8, BAD, IRF1, CASP1, SCX, ENG, TNFRSF1A*	9 / 71	1.9	8.94E-02
GO:0051898	negative regulation of protein kinase B signaling	*LMBRD1, MSTN, MUL1, CIB1, LEMD2, PHLDA3*	6 / 37	2.5	8.98E-02
GO:0090263	positive regulation of canonical Wnt signaling pathway	*PSMD12, JUP, DAPK3, AXIN1, PPP2R3A, PSMB9, LRP6, PSMA5, PSMD7, PSMD2, UBR5, PSME4, LGR4*	13 / 120	1.7	9.08E-02
GO:0085020	protein K6-linked ubiquitination	*UBE2T, RNF6, PARK2*	3 / 8	5.8	9.12E-02
GO:1903214	regulation of protein targeting to mitochondrion	*BNIP3L, PINK1, PARK2*	3 / 8	5.8	9.12E-02
GO:0090277	positive regulation of peptide hormone secretion	*RASL10B, TFR2, FGG*	3 / 8	5.8	9.12E-02
GO:0001766	membrane raft polarization	*CMTM8, MAL, DOCK2*	3 / 8	5.8	9.12E-02
GO:0042987	amyloid precursor protein catabolic process	*PSEN2, DHCR24, ABCG1*	3 / 8	5.8	9.12E-02
GO:0042102	positive regulation of T cell proliferation	*TNFSF4, DHPS, NCK2, PRKCQ, LILRB2, CD46, CORO1A, TNFSF13B*	8 / 60	2.0	9.36E-02
GO:0032728	positive regulation of interferon-beta production	*IFIH1, RNF135, DDX3X, IRF1, ZC3HAV1*	5 / 27	2.8	9.51E-02
GO:0042752	regulation of circadian rhythm	*MAPK10, PER1, MAPK8, MAGED1, CRY2, NR1D2, PML*	7 / 49	2.2	9.69E-02
GO:0008625	extrinsic apoptotic signaling pathway via death domain receptors	*DDX3X, BAD, BLOC1S2, CD27, PIK3R1, TNFRSF1A*	6 / 38	2.4	9.82E-02
GO:0030049	muscle filament sliding	*ACTC1, DES, MYH3, MYBPC2, NEB, TTN*	6 / 38	2.4	9.82E-02
GO:0007257	activation of JUN kinase activity	*ERN1, MAP3K2, MAP4K2, DBNL, AXIN1, MUL1*	6 / 38	2.4	9.82E-02
GO:0050900	leukocyte migration	*LYN, SLC16A1, ITGA3, ITGB2, PIK3R2, PIK3CB, PIK3R1, MIF, MERTK, MMP9, MAG, SLC7A7, SIRPA*	13 / 122	1.6	9.96E-02

Supplementary table 9. Gene ontology analysis: differentially expressed genes affected by DNA methylation (52 w). The GO-terms colored in green are depicted in the go plot in fig.4b

GO-term	Pathway	Gene symbol	Number of genes / total number of genes in pathway	Fold enrichment	P-value
GO:0006468	protein phosphorylation	GUCY2D, TP53RK, CCNH, MAST2, PRKAG2, PIK3R1, NLK, KALRN, MYLK4, MAPK8, RPS6KA5, GRK4, STK38, PDK4, STK38L, CSK, PRKG1, LYN, MAP4K2, CDK19, DMPK, MMD, STRADB, LIMK1, SMAD9, PHKB, MERTK, FGR, ERN1, BCR, PINK1, FES, STK25, MAPKAPK2, AAK1, SGK1, STK40	37 / 456	2.4	1.6E-06
GO:0005977	glycogen metabolic process	PPP1R1A, PPP1R3B, PPP1R3E, PPP1R3D, PRKAG2, PHKB, PYGL, SLC37A4, STK40	9 / 29	9.3	3.3E-06
GO:0034976	response to endoplasmic reticulum stress	ERN1, ERP44, CREB3L2, FAM129A, UBQLN1, TXNDC11, PIK3R2, ATP2A1, PIK3R1, PARK2, ATF4	11 / 75	4.4	1.8E-04
GO:0007050	cell cycle arrest	CDKN2B, CAB39, CUL3, STRADB, AK1, PRKAG2, DHCR24, VASH1, PML, ERN1, PPM1A, IRF1, LAMTOR1, MAP2K6	14 / 141	3.0	8.7E-04
GO:0035556	intracellular signal transduction	GUCY2D, CAB39, DGKA, MAST2, PRKAG2, NLK, ASB18, KALRN, ADCY7, RPS6KA5, RASSF5, STK38, STK38L, GPR155, POLR2M, MAP4K2, MAP3K3, BCR, PINK1, PPP1R1A, PRKCQ, ASB5, ASB2, SGK1, SIPA1, RAPGEF3, GCOM1	27 / 403	2.0	1.1E-03
GO:0006853	carnitine shuttle	CPT1A, PRKAG2, SLC25A20, ACACB	4 / 9	13.3	2.7E-03
GO:0031623	receptor internalization	CXCR1, GRK4, NEDD4, ITGB2, CLTC, CD9, SNCA	7 / 43	4.9	2.8E-03
GO:0046320	regulation of fatty acid oxidation	FABP3, PDK4, PRKAG2	3 / 3	30.0	3.2E-03
GO:0000209	protein polyubiquitination	DTX3L, SMURF1, CUL3, PARK2, PSMB9, DDB2, RNF41, ZNRF1, NPEPPS, PSMD2, UBR5, PSME4, UBE2V1, LONRF1, ASB2	15 / 184	2.4	3.5E-03
GO:0050900	leukocyte migration	LYN, MAG, SLC7A7, SLC16A1, ITGB2, SIRPA, PIK3R2, PIK3CB, PIK3R1, MERTK, MMP9	11 / 122	2.7	7.6E-03
GO:0043161	proteasome-mediated ubiquitin-dependent protein catabolic process	SMURF1, SIAH3, CUL3, AXIN1, WWP1, ARRB1, KCTD5, RAD23B, PARK2, PML, PSMB9, ZNRF1, RNF216, PSMD2, PSME4	15 / 203	2.2	8.2E-03
GO:0018105	peptidyl-serine phosphorylation	PINK1, MAPK8, DMPK, CAB39, MAST2, STK38, MAPKAPK2, STK38L, PRKCQ, SGK1, VRK3	11 / 125	2.6	9.0E-03
GO:0010991	negative regulation of SMAD protein complex assembly	PPM1A, PMEPA1, LDLRAD4	3 / 5	18.0	1.0E-02
GO:0010800	positive regulation of peptidyl-threonine phosphorylation	CAB39, EGF, AXIN1, PRKAG2, CALM1	5 / 27	5.6	1.2E-02
GO:0090002	establishment of protein localization to plasma membrane	EFR3A, JUP, TSPAN5, EFR3B, FAM126A, TNFRSF1A	6 / 42	4.3	1.2E-02
GO:0046777	protein autophosphorylation	LYN, DDR1, MAP3K3, MYLK2, NLK, ERN1, BCR, FGR, FES, STK25, MAPKAPK2, AAK1, CSK	13 / 172	2.3	1.3E-02
GO:0016192	vesicle-mediated transport	NSF, RAB10, ACTR1A, AP1G1, FNBP1, SAR1B, CLTC, NCK2, SPIRE1, KALRN, PARK2, VTI1B	12 / 152	2.4	1.3E-02
GO:0046854	phosphatidylinositol phosphorylation	EFR3A, IMPA2, EGF, PIP4K2A, PIK3R2, PIK3CB, PIK3R1, EFR3B, FAM126A	9 / 94	2.9	1.3E-02
GO:0006513	protein monoubiquitination	UBE2T, CUL3, NEDD4, UBE2O, PARK2	5 / 28	5.4	1.3E-02
GO:0030168	platelet activation	LYN, C6ORF25, DGKA, FGG, CD9, PRKCQ, ARRB1, PIK3CB, PIK3R1, MERTK	10 / 115	2.6	1.5E-02
GO:0032237	activation of store-operated calcium channel activity	STIM1, ASPH, TMEM110	3 / 6	15.0	1.5E-02
GO:0030512	negative regulation of transforming growth factor beta receptor signaling pathway	PPM1A, CHST11, SMURF1, HTRA4, PMEPA1, LDLRAD4, ENG	7 / 64	3.3	1.9E-02
GO:0031398	positive regulation of protein ubiquitination	PINK1, RASSF5, AXIN1, UBQLN1, ARRB1, PRICKLE1, BIRC3	7 / 64	3.3	1.9E-02
GO:0071901	negative regulation of protein serine/threonine kinase activity	CDKN2B, PRKAG2, CIB1, LRP6	4 / 18	6.7	2.1E-02
GO:0070050	neuron cellular homeostasis	CHRNA1, FGGY, PARK2	3 / 7	12.9	2.1E-02
GO:0001867	complement activation, lectin pathway	FCN3, KRT1, MASP1	3 / 7	12.9	2.1E-02
GO:0014070	response to organic cyclic compound	LYN, ABCC4, CPT1A, SLC37A4, ACACB, ACAT1	6 / 49	3.7	2.3E-02
GO:0070059	intrinsic apoptotic signaling pathway in response to endoplasmic reticulum stress	ERN1, TMBIM6, ATP2A1, PML, ATF4	5 / 33	4.5	2.3E-02
GO:0008285	negative regulation of cell proliferation	LYN, DDR1, MEG3, CDKN2B, WARS, TESC, DHCR24, CIB1, ETS1, PML, RNF41, FABP3, BCL6, RASSF5, IRF1, ALDH1A2, FTH1, NCK2, CD9, CSK, QSOX1, FEZF2	22 / 396	1.7	2.5E-02
GO:0051865	protein autoubiquitination	TRIM71, UBE2T, RNF141, PARK2, RNF41, DDB2	6 / 50	3.6	2.5E-02
GO:0007165	signal transduction	ARRB1, PIK3CB, TNFSF13B, IMPA2, NKIRAS2, NCK2, PRKG1, FGG, AXIN1, PPP2R5A, FAM126A, TNFRSF1A, BCR, APBB1IP, TRAF3, FEZ2, RIN3, PPP1R12B, MAPRE2, RAPGEF3, NSMAF, CHRNA1, CSF2RB, PIK3R2, PIK3R1, KALRN, CD79B, GRK4, SPOCK2, SPOCK1, MAP2K6, CAP2, LYN, JUP, EGF, TXNRD1, LIMK1, WWP1, ARHGAP28, ARHGAP26, NR2F6, GRIN2D, ARHGAP32, CLCN6, ARHGAP30, GNAL, PPP1R1A, STK25, HDGF, ASB2, LRP12, SIPA1	52 / 1161	1.3	2.6E-02
GO:1903214	regulation of protein targeting to mitochondrion	BNIP3L, PINK1, PARK2	3 / 8	11.2	2.7E-02
GO:1903076	regulation of protein localization to plasma membrane	KALRN, GBP1, VTI1B	3 / 8	11.2	2.7E-02
GO:0034097	response to cytokine	STAT1, ALDH1A2, MAPKAPK2, IL6R, PML, BCL2L1	6 / 52	3.5	2.9E-02
GO:0030036	actin cytoskeleton organization	NISCH, BCR, BCL6, EPB41, LIMK1, SPIRE1, DOCK2, ARHGAP26, PRKG1, PDLIM7	10 / 130	2.3	3.0E-02
GO:0005975	carbohydrate metabolic process	GALC, FGGY, GUSBP11, ST8SIA1, AMDHD2, MLEC, ST3GAL5, PYGL, NAGPA, KIAA1161, GALK2, LDHAL6A	12 / 174	2.1	3.1E-02
GO:0016567	protein ubiquitination	SMURF1, CUL3, WWP1, ARRB1, DCAF5, DTX4, FBXO32, ASB18, BACH1, PARK2, SH3RF2, PINK1, RAB40C, KLHL5, TRAF3, NEDD4, ASB5, RNF130, PDZRN4, BIRC3	20 / 359	1.7	3.2E-02
GO:0070534	protein K63-linked ubiquitination	UBE2T, NEDD4, UBE2O, UBE2V1, PARK2	5 / 37	4.1	3.4E-02
GO:0038095	Fc-epsilon receptor signaling pathway	LYN, MAPK8, PSMD2, PSME4, PIK3R2, PRKCQ, GAB2, UBE2V1, PIK3CB, PIK3R1, CALM1, PSMB9	12 / 178	2.0	3.6E-02
GO:0007173	epidermal growth factor receptor signaling pathway	RPS6KA5, FES, EGF, NCK2, CSK, PIK3R1	6 / 56	3.2	3.8E-02
GO:0045944	positive regulation of transcription from RNA polymerase II promoter	CCNH, CHD7, NLRC5, GATA5, FOXK2, ARRB1, ARID4B, PIK3R2, PIK3R1, ETS1, BACH1, PARK2, LRP6, RPS6KA5, RAD21, CREB3L2, KPNA6, NCK2, OTX1, HOXA7, FEZF2, EGLN1, CDKN2B, PRRX1, CDX1, STAT1, ZBTB38, RFX3, ESRRG, GRHL2, PML, TNFRSF1A, MED25, COPS5, TFDP1, CCPG1, TFR2, IRF1, RFX4, IRF2, AGO2, LPIN1, ATF4, ENG	44 / 981	1.3	3.9E-02
GO:0071398	cellular response to fatty acid	CPT1A, XRCC5, PDK4, LDLR	4 / 23	5.2	4.0E-02
GO:0001771	immunological synapse formation	DOCK8, NCK2, DOCK2	3 / 10	9.0	4.2E-02
GO:0051272	positive regulation of cellular component movement	LYN, BCL6, ETS1	3 / 10	9.0	4.2E-02
GO:0038083	peptidyl-tyrosine autophosphorylation	LYN, FGR, DDR1, FES, CSK	5 / 40	3.7	4.3E-02
GO:0070373	negative regulation of ERK1 and ERK2 cascade	LYN, DUSP3, CSK, ARRB1, VRK3, GBP1	6 / 58	3.1	4.3E-02
GO:0033209	tumor necrosis factor-mediated signaling pathway	TNFRSF6B, TRAF3, STAT1, PSMD2, PSME4, TNFSF13B, PSMB9, BIRC3, TNFRSF1A	9 / 118	2.3	4.3E-02
GO:0007596	blood coagulation	LYN, EHD1, GGCX, HBG2, C6ORF25, DOCK8, IRF1, FGG, IRF2, GATA5, ANO6, SERPINA5	12 / 184	2.0	4.4E-02
GO:0036498	IRE1-mediated unfolded protein response	ERN1, DNAJC3, SEC61A1, SERP1, HDGF, EXTL3	6 / 59	3.0	4.6E-02
GO:0016579	protein deubiquitination	USP48, USP38, COPS5, WDR48, USP3, USP12, UCHL3, USP39	8 / 99	2.4	4.7E-02
GO:0001933	negative regulation of protein phosphorylation	LYN, FAM129A, CIB1, PARK2, LRP6, SNCA	6 / 61	2.9	5.2E-02
GO:0000082	G1/S transition of mitotic cell cycle	TRIM71, CCNH, CUL3, MCM4, MCM5, EIF4E, GSPT1, MCM2	8 / 102	2.4	5.4E-02
GO:0060394	negative regulation of pathway-restricted SMAD protein phosphorylation	PMEPA1, LDLRAD4, ENG	3 / 12	7.5	5.9E-02
GO:0042574	retinal metabolic process	ALDH1A2, AKR1C3, ALDH8A1	3 / 12	7.5	5.9E-02
GO:0098779	mitophagy in response to mitochondrial depolarization	PINK1, SMURF1, PARK2	3 / 12	7.5	5.9E-02
GO:0014065	phosphatidylinositol 3-kinase signaling	PIK3R2, PIK3CB, PIK3R1, NYAP2	4 / 27	4.4	5.9E-02
GO:0006470	protein dephosphorylation	PPM1A, PTPRE, PDP2, MYH3, PPM1L, PPP1R3B, PPP1R3D, CPPED1, LHPP	9 / 126	2.1	6.0E-02
GO:0042632	cholesterol homeostasis	EHD1, FABP3, LAMTOR1, SLC37A4, LDLR, ABCG1	6 / 64	2.8	6.2E-02
GO:0001934	positive regulation of protein phosphorylation	RAP2A, PINK1, FAM129A, AXIN1, CDK2AP1, CIB1, ARRB1, MMP9, ENG	9 / 127	2.1	6.2E-02
GO:0055114	oxidation-reduction process	ALDH1L1, MTRR, NDUFA10, HSD17B8, CYB561D2, SH3PXD2A, FTH1, QSOX1, LBR, SNCA, EGLN1, ABCC4, KDM4C, RNLS, TXNRD1, TXNL1, GSR, AKR1C3, HSD11B1L, DHCR24, CYP27A1, POR, ASPH, ALDH1A2, NDUFAF5, GFOD2, ALDH8A1, LDHAL6A	28 / 592	1.4	6.2E-02
GO:0043388	positive regulation of DNA binding	EGF, CALM1, MMP9, PARK2	4 / 28	4.3	6.5E-02
GO:0010872	regulation of cholesterol esterification	LAMTOR1, ABCG1	2 / 2	30.0	6.5E-02
GO:0050992	dimethylallyl diphosphate biosynthetic process	IDI1, IDI2	2 / 2	30.0	6.5E-02
GO:0009240	isopentenyl diphosphate biosynthetic process	IDI1, IDI2	2 / 2	30.0	6.5E-02
GO:0001817	regulation of cytokine production	LYN, TRAF3, CSK	3 / 13	6.9	6.8E-02
GO:0045088	regulation of innate immune response	FGR, IRF1, BIRC3	3 / 13	6.9	6.8E-02
GO:0050790	regulation of catalytic activity	DPM2, DMPK, BAG2, PPP1R3B, PRKAG2, SGK1	6 / 66	2.7	6.8E-02
GO:0015031	protein transport	ENY2, PKDCC, RAB5C, LMAN2L, PIK3R2, ARRB1, ABCB9, SCAMP2, ARCN1, KTN1, SEC61A1, C16ORF62, AP5M1, SERP1, RAB36, CHMP1B, SPIRE1, EXOC6, SLC15A4, TMED5	20 / 395	1.5	7.0E-02
GO:0042787	protein ubiquitination involved in ubiquitin-dependent protein catabolic process	HERC4, AREL1, SMURF1, SIAH3, CUL3, NEDD4, UBR5, WWP1, ASB2, PARK2	10 / 153	2.0	7.0E-02
GO:0007163	establishment or maintenance of cell polarity	CRB1, STK25, SYNE2, CAP2	4 / 29	4.1	7.1E-02
GO:0048812	neuron projection morphogenesis	KIRREL3, DBNL, ZSWIM6, NYAP2, SGK1	5 / 48	3.1	7.5E-02
GO:0042573	retinoic acid metabolic process	SCPEP1, ALDH1A2, ALDH8A1	3 / 14	6.4	7.7E-02
GO:0008299	isoprenoid biosynthetic process	IDI1, IDI2, ISPD	3 / 14	6.4	7.7E-02
GO:0048148	behavioral response to cocaine	HOMER1, KALRN, SNCA	3 / 14	6.4	7.7E-02
GO:0005513	detection of calcium ion	STIM1, ASPH, CALM1	3 / 14	6.4	7.7E-02
GO:0051056	regulation of small GTPase mediated signal transduction	RAP1GAP2, BCR, ARHGAP32, ARHGAP30, PIK3R2, ARHGAP28, ARHGAP26, KALRN, SIPA1	9 / 134	2.0	7.9E-02
GO:0042147	retrograde transport, endosome to Golgi	CLTC, UBE2O, DENND5A, EVI5, CLN5, VTI1B	6 / 69	2.6	8.0E-02
GO:0045765	regulation of angiogenesis	EGLN1, WARS, KRT1, ETS1	4 / 31	3.9	8.3E-02
GO:1904707	positive regulation of vascular smooth muscle cell proliferation	ERN1, DDX39B, MMP9	3 / 15	6.0	8.7E-02
GO:0001953	negative regulation of cell-matrix adhesion	BCL6, PIK3R1, HOXA7	3 / 15	6.0	8.7E-02
GO:0090141	positive regulation of mitochondrial fission	PINK1, DDHD1, PARK2	3 / 15	6.0	8.7E-02
GO:2000060	positive regulation of protein ubiquitination involved in ubiquitin-dependent protein catabolic process	SMURF1, EGF, AXIN1	3 / 15	6.0	8.7E-02
GO:0060333	interferon-gamma-mediated signaling pathway	STAT1, IFNGR2, IRF1, IRF2, GBP1, PML	6 / 71	2.5	8.8E-02
GO:0006974	cellular response to DNA damage stimulus	LYN, TIGAR, DTX3L, BCL6, UBE2T, ZBTB38, UBR5, MAPKAPK2, PSME4, SUSD6, CIB1, SGK1	12 / 208	1.7	8.9E-02
GO:0042127	regulation of cell proliferation	FGR, TNFRSF6B, JUP, CHST11, BCL6, FES, CSK, CIB1, SGK1, ENG, TNFRSF1A	11 / 185	1.8	9.1E-02
GO:0043547	positive regulation of GTPase activity	DOCK8, ARRB1, CSF2RB, PIK3R2, KALRN, ADAP2, NCK2, DENND5A, RAP1GAP2, FNBP1, EGF, HPS1, AXIN1, ARHGAP28, ARHGAP26, GRIN2D, BCR, ARHGAP32, SNX18, ARHGAP30, RIN3, LAMTOR1, DOCK2, CALM1, SIPA1, RAPGEF3	26 / 565	1.4	9.2E-02
GO:0006915	apoptotic process	PPP1R13B, ITGB2, APIP, CIB1, ARRB1, RNF216, WDR92, RASSF5, RAD21, RNF130, SNCA, MAP2K6, TIGAR, AREL1, STAT1, SIAH3, AXIN1, DHCR24, ZDHHC16, PML, NISCH, TRAF3, IRF1, SGK1, BIRC3, MCM2	26 / 567	1.4	9.2E-02

GO:0042472	inner ear morphogenesis	BCR, PRRX1, CHD7, OTX1, SOBP	5 / 52	2.9	9.4E-02
GO:0032880	regulation of protein localization	TBC1D1, MAPK8, MMD, AAK1, B9D1	5 / 52	2.9	9.4E-02
GO:0002931	response to ischemia	TIGAR, RNLS, CIB1, MAP2K6	4 / 33	3.6	9.6E-02
GO:2000116	regulation of cysteine-type endopeptidase activity	PSMB9, BIRC3	2 / 3	20.0	9.7E-02
GO:1903204	negative regulation of oxidative stress-induced neuron death	PINK1, ATF4	2 / 3	20.0	9.7E-02
GO:1903202	negative regulation of oxidative stress-induced cell death	PINK1, PARK2	2 / 3	20.0	9.7E-02
GO:1900407	regulation of cellular response to oxidative stress	PINK1, PARK2	2 / 3	20.0	9.7E-02
GO:1903298	negative regulation of hypoxia-induced intrinsic apoptotic signaling pathway	PINK1, TMBIM6	2 / 3	20.0	9.7E-02
GO:1901525	negative regulation of macromitophagy	TIGAR, RNF41	2 / 3	20.0	9.7E-02
GO:1902803	regulation of synaptic vesicle transport	PINK1, PARK2	2 / 3	20.0	9.7E-02
GO:0051642	centrosome localization	NIN, ASUN, SYNE2	3 / 16	5.6	9.8E-02
GO:0035269	protein O-linked mannosylation	DPM2, B4GAT1, ISPD	3 / 16	5.6	9.8E-02
GO:0031396	regulation of protein ubiquitination	PINK1, UBQLN1, PARK2	3 / 16	5.6	9.8E-02
GO:0045727	positive regulation of translation	PINK1, SERP1, DDX39B, FAM129A, RBMS3	5 / 53	2.8	9.9E-02

Supplementary table 10. Gene ontology analysis of correlated genes to fasting glucose levels. The lines colored in green are listed in the text.

GO-term	Pathway	Gene symbol	Number of genes / total number of genes in pathway	Fold enrichment	P-value
GO:0007605	sensory perception of sound	*TBX1, MARVELD2, GJB3, HOMER2, MYO3A, PCDH15, SLC26A4, OTOR, POU3F4*	9 / 133	4.26	1.3E-03
GO:0071395	cellular response to jasmonic acid stimulus	*AKR1C1, AKR1C3, AKR1C4*	3 / 4	47.17	1.5E-03
GO:2000463	positive regulation of excitatory postsynaptic potential	*STX1B, NLGN1, NRXN1, PRKCZ*	4 / 20	12.58	3.7E-03
GO:0044597	daunorubicin metabolic process	*AKR1C1, AKR1C3, AKR1C4*	3 / 8	23.58	6.6E-03
GO:0044598	doxorubicin metabolic process	*AKR1C1, AKR1C3, AKR1C4*	3 / 8	23.58	6.6E-03
GO:0016338	calcium-independent cell-cell adhesion via plasma membrane cell-adhesion molecules	CLDN22, CLDN20, CLDN14	3 / 21	8.98	4.3E-02
GO:0007010	cytoskeleton organization	FGD3, ABLIM1, ZMYM3, MAST4, MAST1, C21ORF2, KRT6B	7 / 161	2.73	4.3E-02
GO:0097091	synaptic vesicle clustering	*NLGN1, NRXN1*	2 / 3	41.93	4.7E-02
GO:0055114	oxidation-reduction process	*BCKDHA, GPX2, MAOB, HGD, AKR1C1, AKR1C3, MOXD2P, AKR1C4, DHRSX, CYP11B2, IYD, HSD17B2, RDH16, PYROXD2, TXNDC8, PRODH*	16 / 592	1.70	5.0E-02
GO:0051260	protein homooligomerization	*P2RX6, NLGN1, SLC22A6, KCNC1, AKR1C1, DERL1, KCTD16*	7 / 177	2.49	6.3E-02
GO:0015721	bile acid and bile salt transport	*AKR1C1, AKR1C4, ABCB11*	3 / 27	6.99	6.8E-02
GO:0050434	positive regulation of viral transcription	*CTDP1, POLR2F, TMEM229A*	3 / 29	6.51	7.7E-02
GO:0097114	NMDA glutamate receptor clustering	*NLGN1, NRXN1*	2 / 5	25.16	7.7E-02
GO:0031427	response to methotrexate	*SLC22A6, ABCC2*	2 / 5	25.16	7.7E-02
GO:0030321	transepithelial chloride transport	*SLC26A6, BEST1*	2 / 5	25.16	7.7E-02
GO:0097104	postsynaptic membrane assembly	*NLGN1, NRXN1*	2 / 6	20.96	9.1E-02
GO:0097119	postsynaptic density protein 95 clustering	*NLGN1, NRXN1*	2 / 6	20.96	9.1E-02
GO:0023041	neuronal signal transduction	*NLGN1, NRXN1*	2 / 6	20.96	9.1E-02
GO:2001054	negative regulation of mesenchymal cell apoptotic process	*TBX1, POU3F4*	2 / 6	20.96	9.1E-02
GO:0009749	response to glucose	*ACVR1C, UCN3, ILDR2, KHK*	4 / 68	3.70	9.3E-02

Suppl. Figure 1. Time course of changes in muscle lipid intermediates.

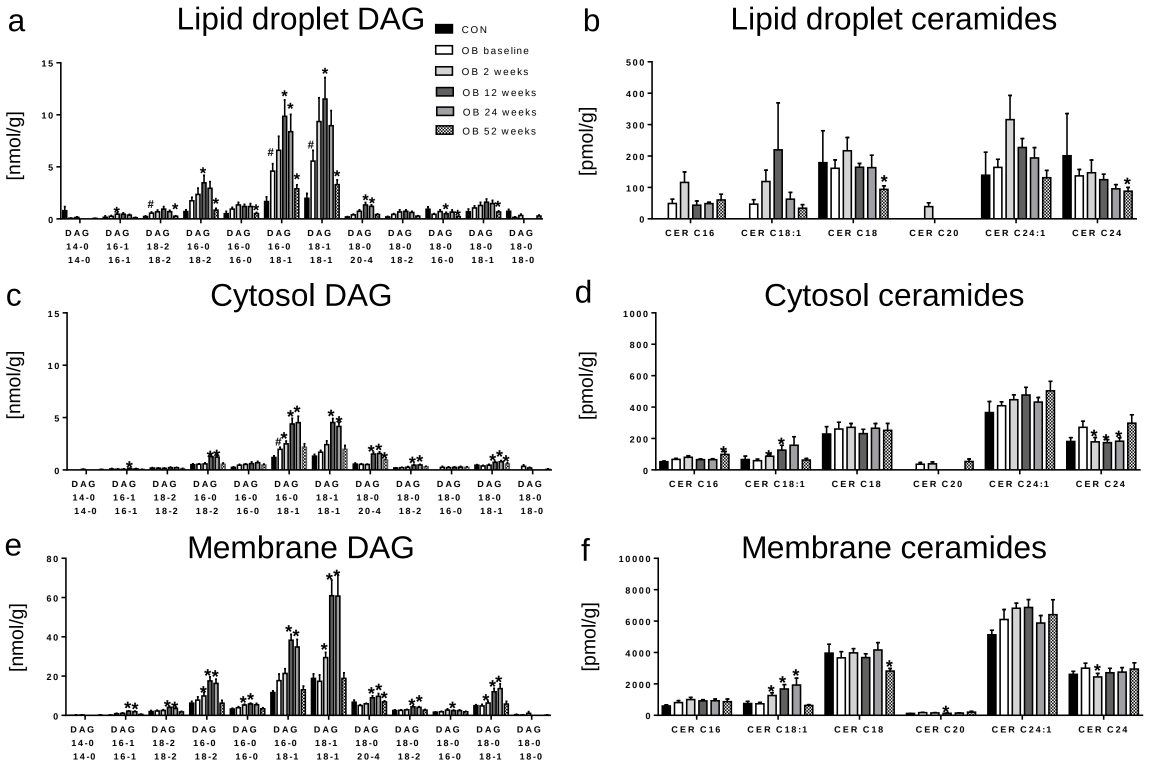

Diacylglycerol (DAG) concentrations in lipid droplet (a), cytosolic (c) and membrane (e) fractions. Ceramide concentrations in lipid droplet (b), cytosolic (d) and membrane (f) fractions. Mean±SEM, *p<0.05 vs OB at baseline using covariance pattern model for repeated measures analysis, #p<0.05 vs CON using unpaired 2-tailed t-test. CON-nonobese humans, OB-obese humans.

Suppl. Figure 2. Time course of changes in muscle mitochondrial electron transport chain complexes.

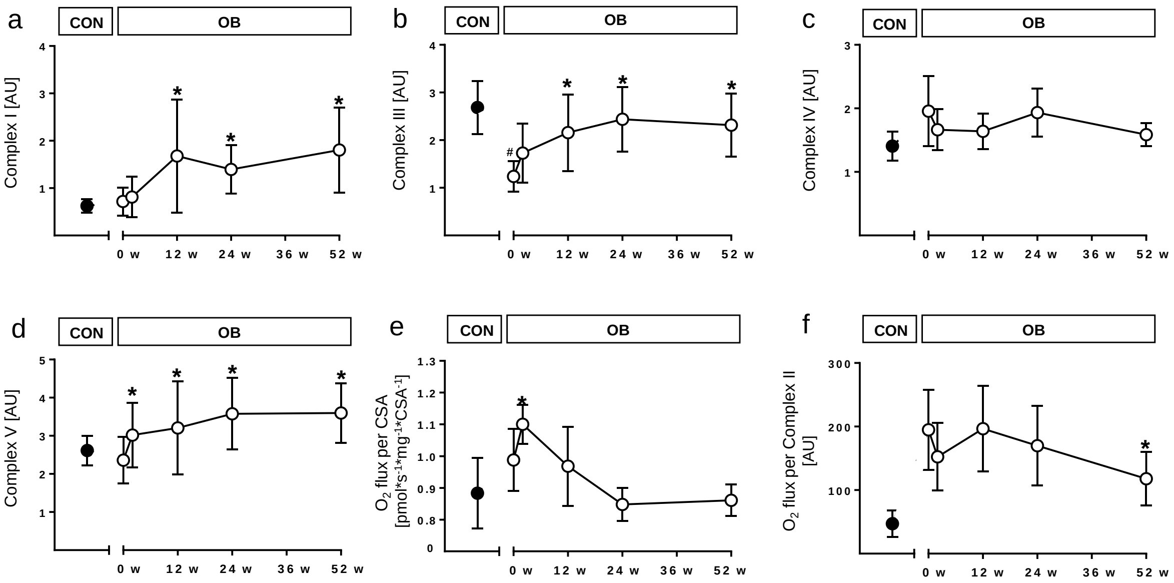

Complex I (NADH:ubiquinone oxidoreductase subunit B8) (a), complex III (ubiquinol-cytochrome C reductase core protein 2) (b), complex IV (cytochrome c oxidase subunit IV) (c), complex V (ATP synthase F1 subunit alpha) (d), maximum mitochondrial respiration per citrate synthase activity (CSA) (e) and maximum mitochondrial respiration per complex II content (f) in obese (empty circles) and in nonobese humans at baseline (black circles). Mean±SEM, *p<0.05 vs OB at baseline using covariance pattern model for repeated measures analysis, #p<0.05 vs CON using unpaired 2-tailed t-test. CON-nonobese humans, OB-obese humans.

Suppl. Figure 3. Time course of changes in muscle protein content of mitochondrial fusion and fission markers.

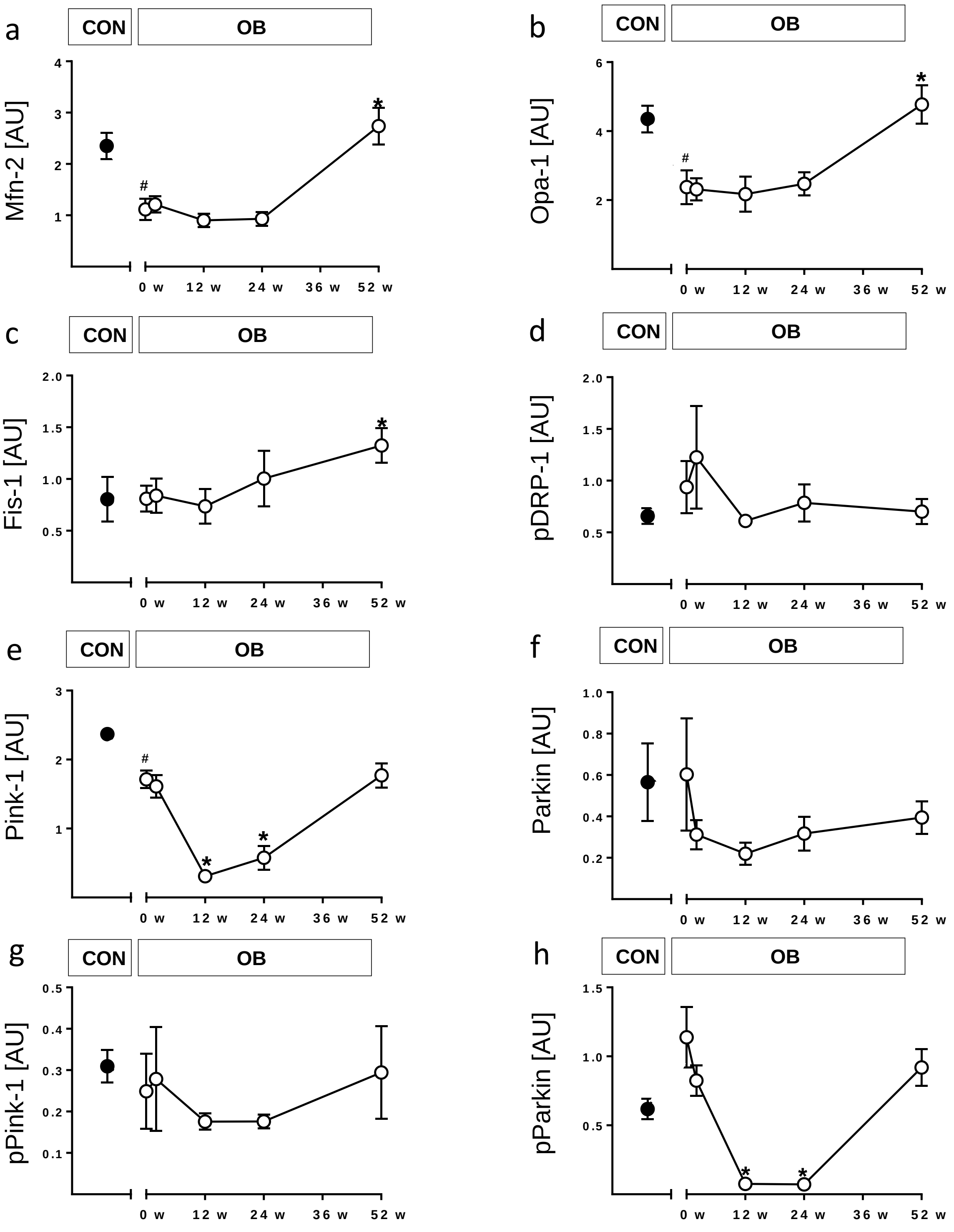

Mitofusin-2 (Mfn-2) (a), optic atrophy 1 protein (Opa-1) (b), mitochondrial fission 1 protein (Fis-1) (c), phospho-Ser^{616}-DRP1 (d), Pink-1 (e), Parkin (f), phospho-Thr^{257}-Pink1 (g) and phospho-Ser^{65}-Parkin (h) in obese (empty circles) and in nonobese humans at baseline (black circles).

Mean±SEM, *p<0.05 vs OB at baseline, using covariance pattern model for repeated measures analysis, #p<0.05 vs CON using unpaired 2-tailed t-test. CON-nonobese humans, OB-obese humans.

Suppl. Figure 4 Mitochondrial volume density from electron microscopy analysis.

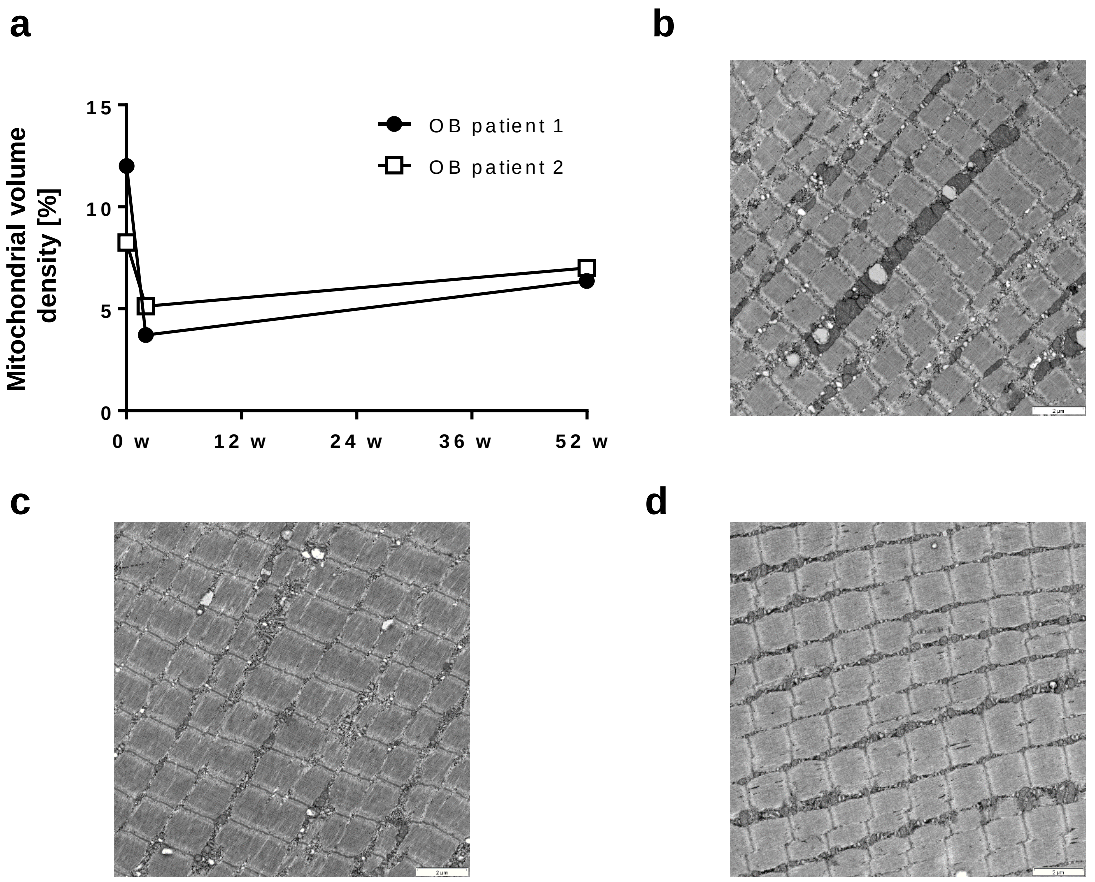

Data from two obese patients (a) and representative micrographs from baseline (b), 2 weeks (c) and 52 weeks (d) after metabolic surgery. Empty squares – obese patient Nr.1, black circles – obese patient Nr. 2. OB-obese humans.

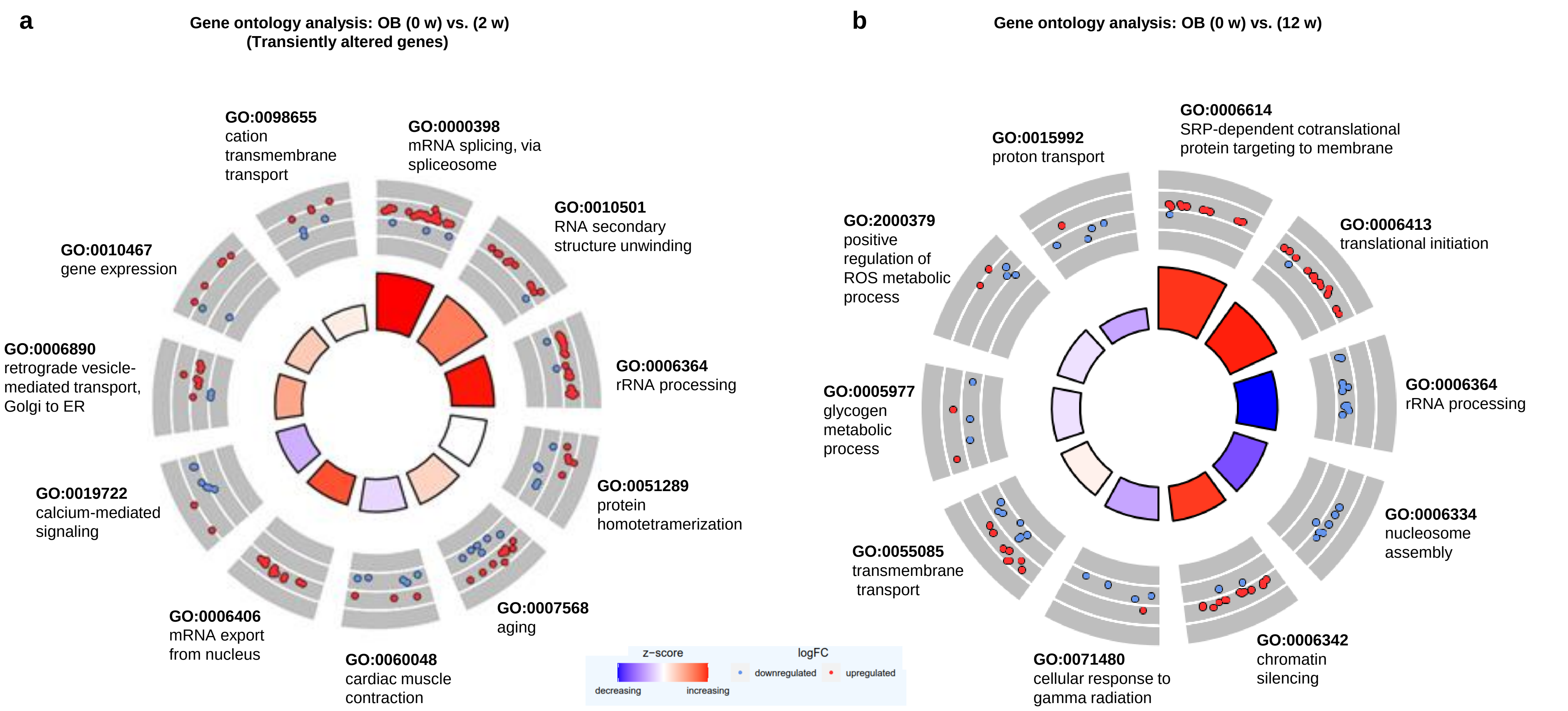

Suppl. Figure 5. Gene ontology analysis of transiently altered at 2 w (a) and transcripts altered at 12 w (b). The inner circle depicts the main processes to be increased (blue) or decreased (red) in obese participants transiently at 2 weeks (a) and 12 weeks (b). The outer circle shows scaled scatter plots for affected genes and their regulation within the most-enriched biological pathway.

Suppl. Figure 6. Alterations in skeletal muscle DNA methylation at 2 and 52 weeks after metabolic surgery.

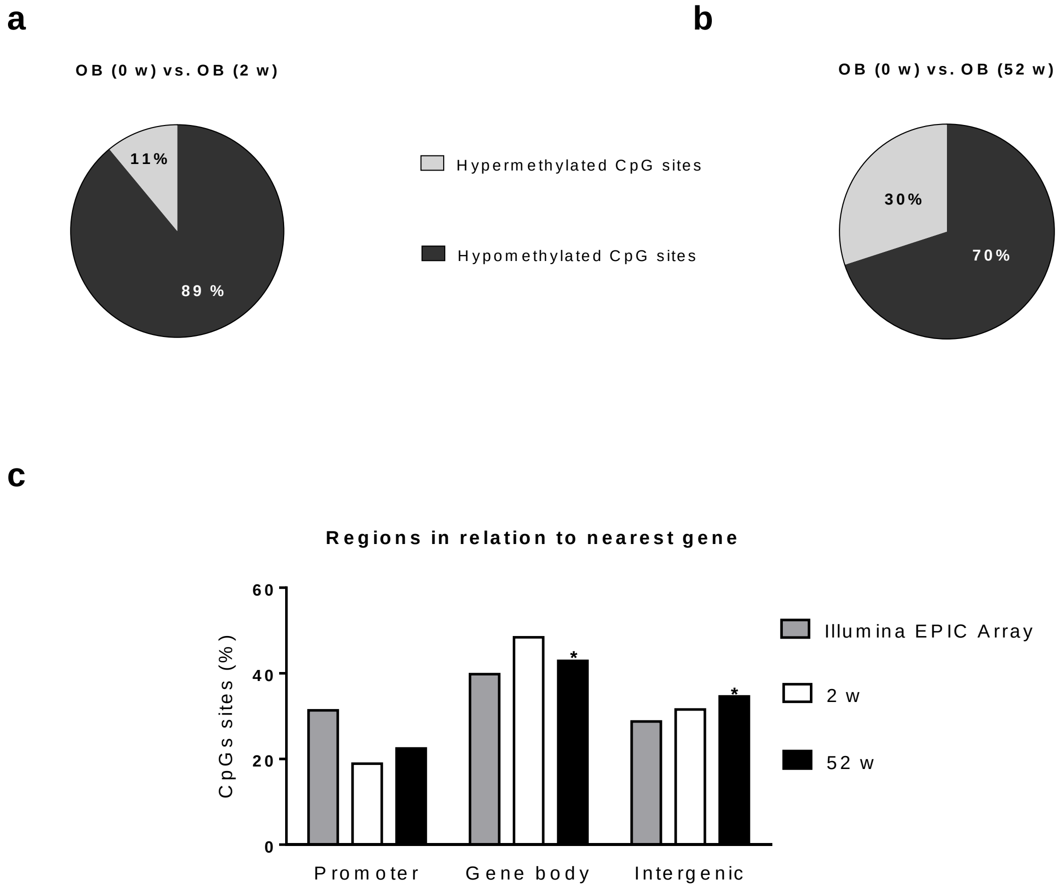

The pie charts show the percentage of individual CpG sites exhibiting significantly increased (bright grey) and decreased (dark grey) methylation at baseline vs. 2 weeks (a) and vs. 52 weeks (b). Localization of differentially methylated CpGs in relation to the nearest gene region (c). Chi-square analysis was performed to test over- or under-representation of sequence features among the differentially methylated CpGs vs. all analyzed CpGs.*p<0.01 (paired t test, n=16), *p<0.05

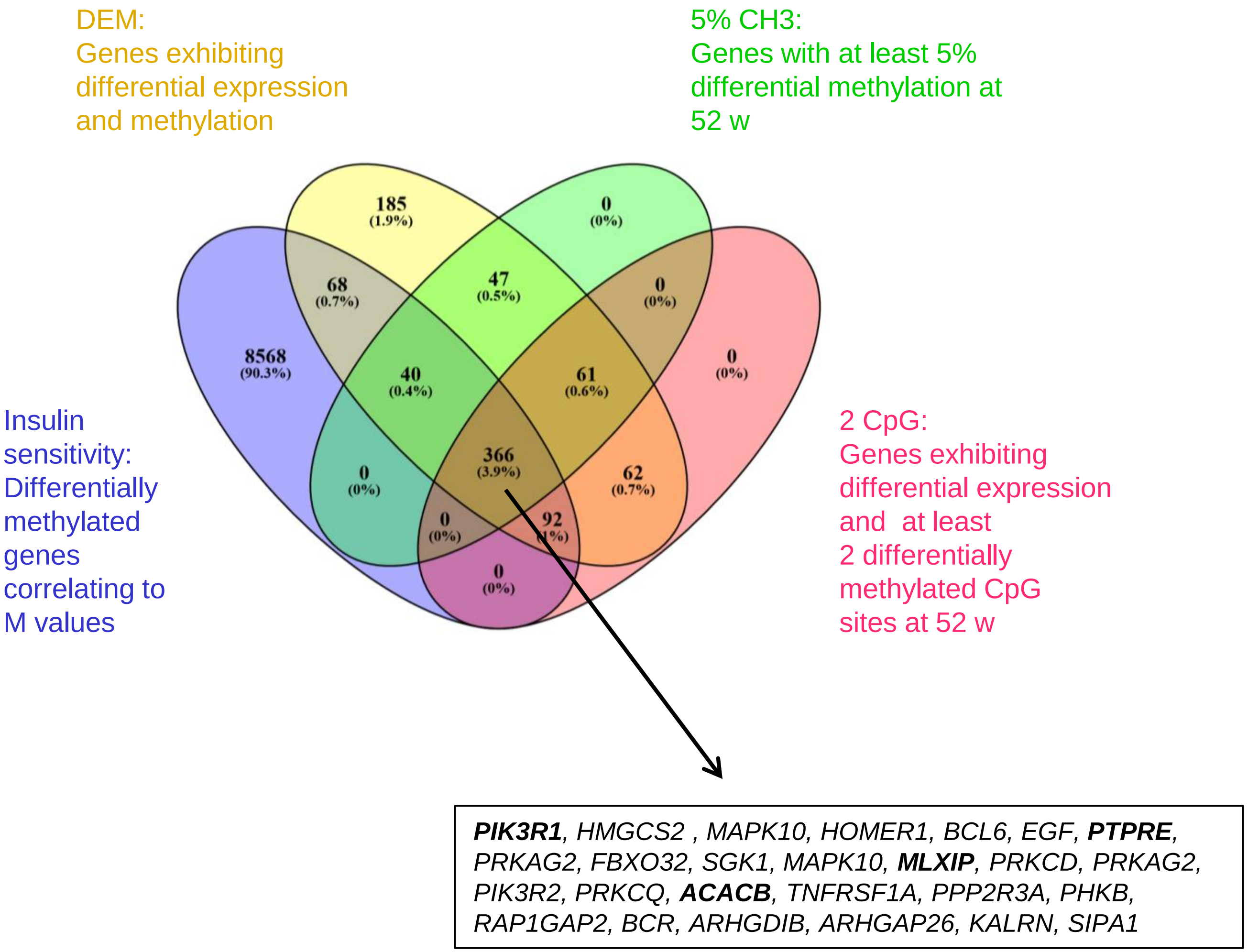

Suppl. Figure 7. Venn diagram summarizing the strategy for the identification of novel candidate genes involved in the improvement of the insulin sensitivity after bariatric surgery. For insulin sensitivity Pearson correlation was performed with all individual DNA methylation
at 0, 2, and 52 w

Suppl. Figure 8. Study design.

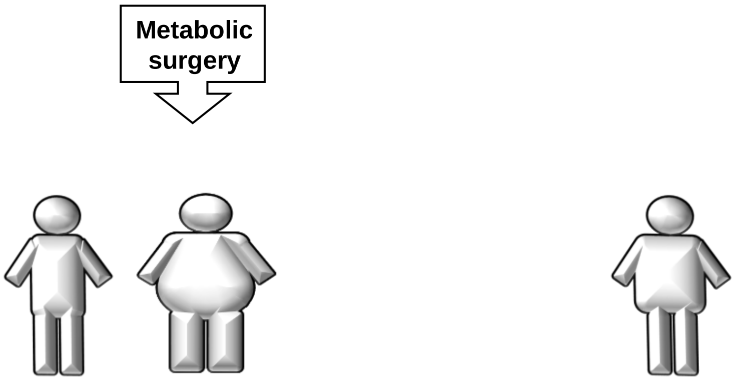

	0 w	2 w	12 w	24 w	52 w
Anthropometry	✔	✔	✔	✔	✔
Lab analyses	✔	✔	✔	✔	✔
Hyperinsulinemic-euglycemic clamp	✔	✔	✔	✔	✔
Muscle mitochondrial densitiy and function	✔	✔	✔	✔	✔
Muscle lipidomics	✔	✔	✔	✔	✔
Muscle genome-wide transcriptomics	✔	✔	✔	✔	✔
Muscle genome-wide methylomics	✔	✔			✔

Obese participants (OB) were intensively characterized at 0 (baseline), 2, 12, 24 and 52 weeks after metabolic surgery. Nonobese humans (CON) were studied at baseline.

Suppl. Figure 9. Time course of mRNA expression of indicated genes

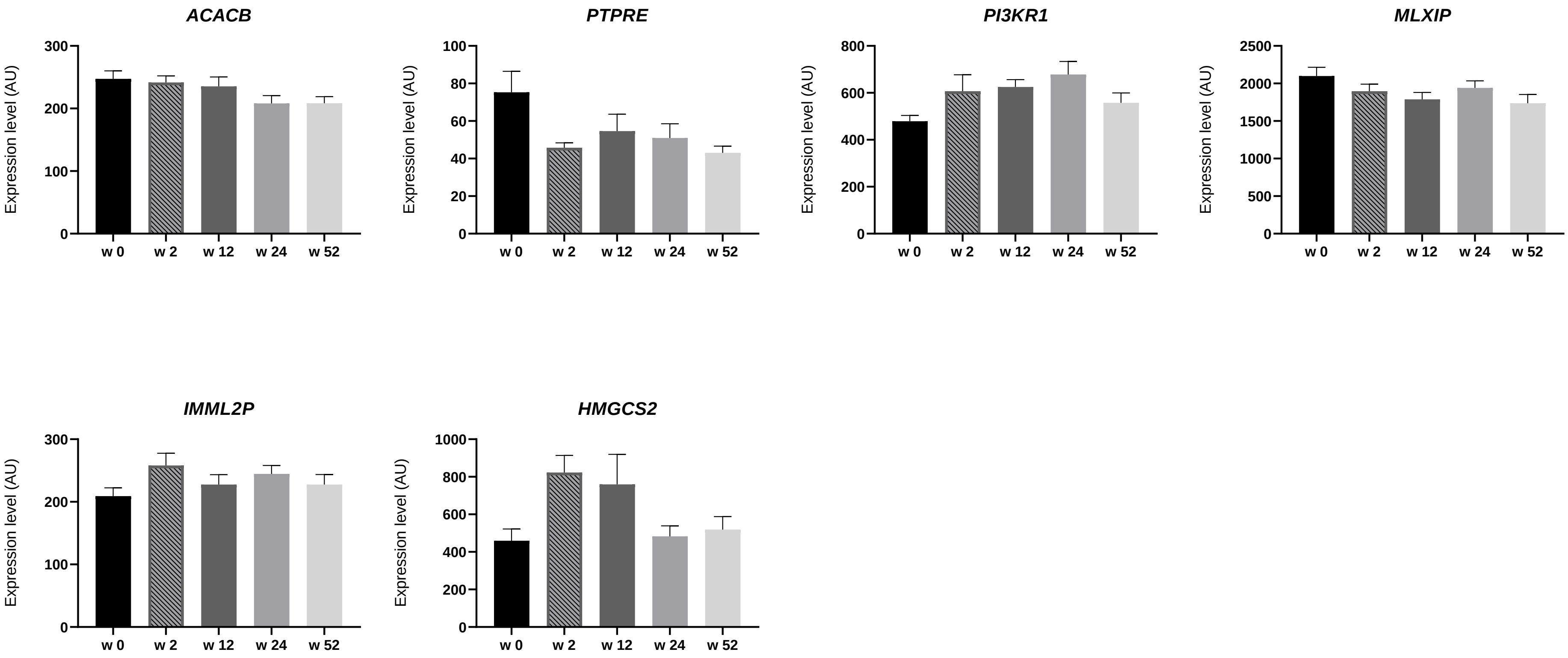

Suppl. Figure 10. DNA methylation levels of *PTPRE* and *PIK3R1* are correlated to peripheral insulin M-value.

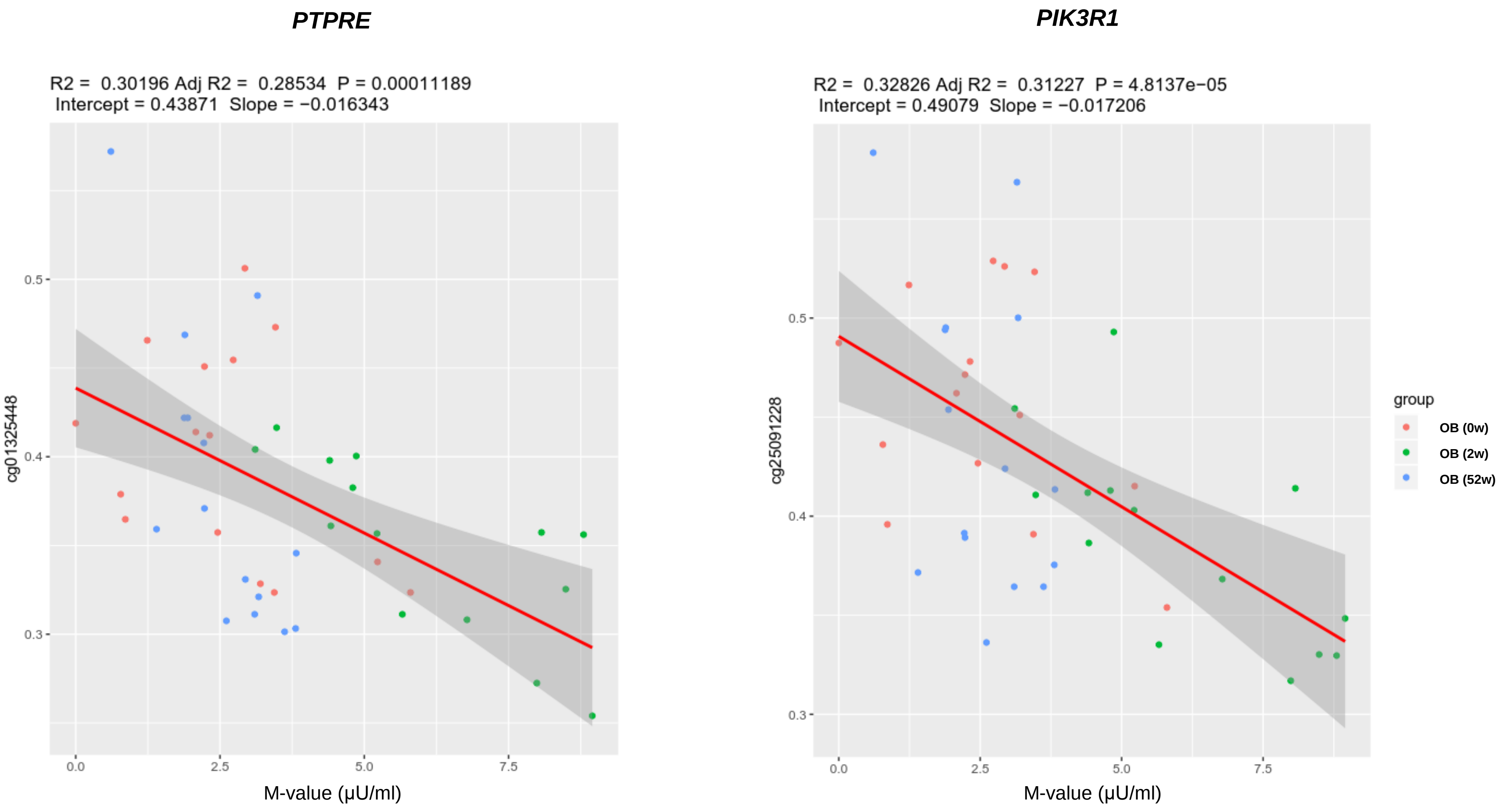

Suppl. Fig. 11. Representative blots for proteins from Western blot analysis

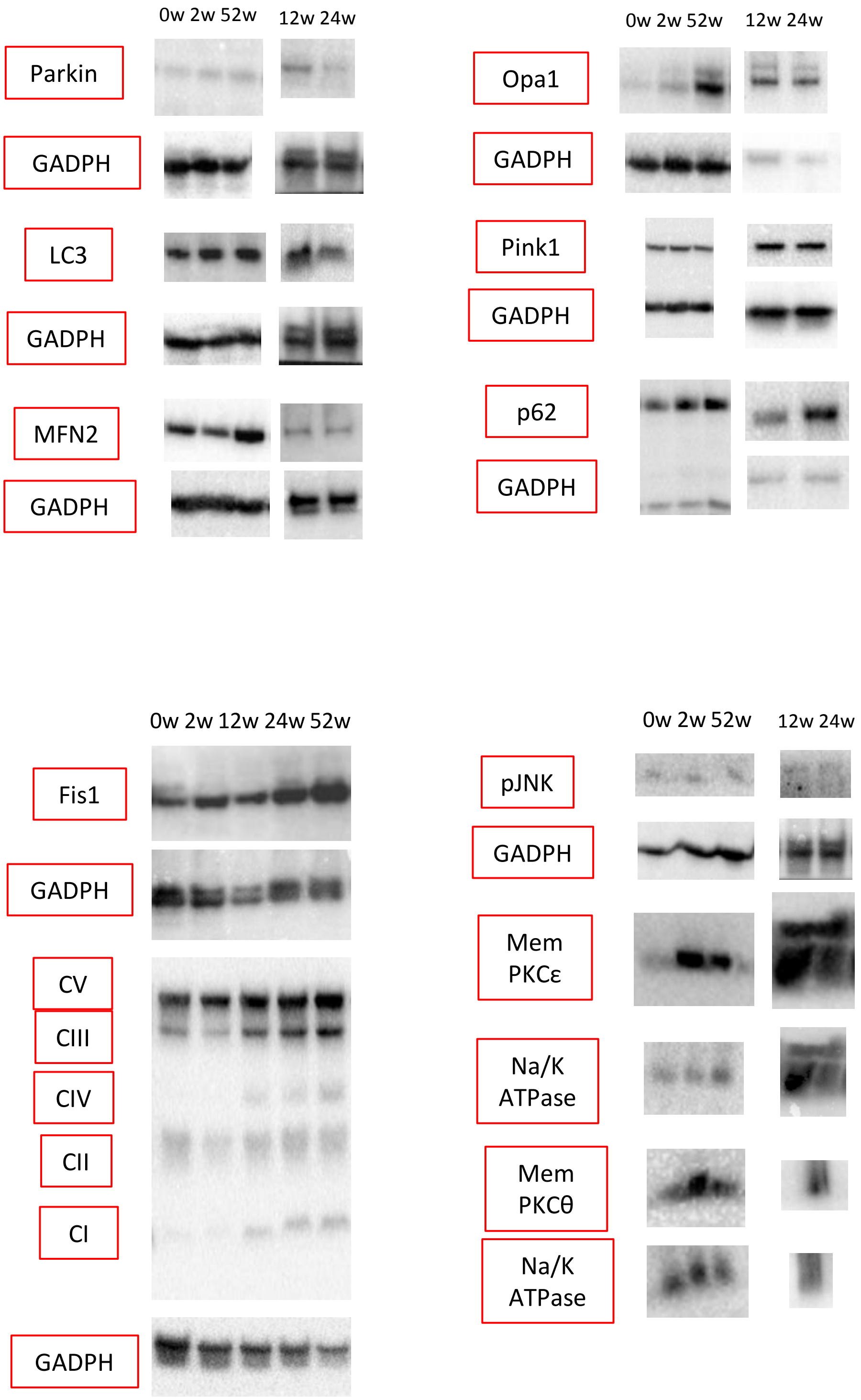

Supplement: Supplementary file 1 — Supplementary Information [file 41467_2019_12081_MOESM1_ESM.pdf]
