# Supplementary material for: Author Correction: Dynamic changes of muscle insulin sensitivity after metabolic surgery
Source: Nat Commun. 2022 Jun 10;13:3353. doi: 10.1038/s41467-022-29350-0 (PMC9187703; doi:10.1038/s41467-022-29350-0)

Supplementary information

Gancheva et al. Dynamic changes of muscle insulin sensitivity after metabolic surgery

Supplementary table 1. Top 13 genes with higher expression in skeletal muscle of OB at 2 weeks after metabolic surgery as compared to baseline.

Gene symbol	Gene name	logFold change	P value	Gene function
MTUS1	Microtubule-associated tumor suppressor 1	-0.97	0.033	Located in mitochondria
TRMT6	tRNA (adenine(58)-N(1))-e non-catalytic subunit TRM6	-0.77	0.037	Methyltransferase responsible for 1-methyl-adenosine at position 58 of human mitochondrial tRNAs
ATP2C2	Calcium-transporting ATPase type 2C member 2	-0.75	0.003	Magnesium-dependent enzyme catalyzing ATP hydrolysis coupled with calcium transport
RAB3D	Ras-related protein Rab-3D	-0.72	0.012	GTPase involved in protein transport
GBP4	Guanylate-binding protein 4	-0.66	10^{-4}	Guanylate-binding protein activated by interferon.
SNAPIN	SNARE-associated protein Snapin	-0.66	0.017	Component of lysosome-related organelle complex required for biogenesis of lysosome-related organelles
FFAR1	Free fatty acid receptor 1	-0.65	0.0082	G-protein coupled receptor for medium- and long-chain (un)saturated fatty acids and involved in glucose homeostasis
SLC27A4	Long-chain fatty acid transport protein 4	-0.64	0.0021	A fatty acid transporter
ZNF329	Zinc finger protein 329	-0.61	0.017	Zinc finger protein
TASP1	Threonine aspartase 1	-0.60	0.021	Endopeptidase cleaving specific substrates following aspartate residues
ABCD4	ATP-binding cassette sub-family D member 4	-0.56	0.002	A member of the ALD subfamily involved in peroxisomal import of fatty acids and/or fatty acyl-CoAs
GTF2E2	Transcription initiation factor IIE subunit beta	-0.55	0.004	Transcription factor that is part of the RNA polymerase II transcription initiation complex
IL18RAP	Interleukin-18 receptor accessory protein	-0.55	0.03	Involved in IL18-dependent signal transduction leading to NF-kappa-B and JNK activation

*p<0.05 (paired t test, n=16). Gene names given in italics.

Supplementary table 2. Top 13 genes with higher expression in skeletal muscle of OB at 52 weeks after bariatric surgery as compared to baseline.

Gene symbol	Gene name	logFold change	P value	Gene function
BACH1	BTB and CNC homology 1	-2.16	0.01	Transcription factor involved in oxidative stress
ALS2CR11	C2 calcium-dependent domain-containing protein 6	-1.62	0.009	
GAMT	Guanidinoacetate N-methyltransferase	-1.33	0.038	Guanidinoacetate N-methyltransferase
SREK1IP1	SREK1-interacting protein 1	-0.97	0.020	
VASH1	Vasohibin 1	-0.88	0.04	
TOP3A	Topoisomerase (DNA) III alpha	-0.85	0.03	
ITIH1	Inter-alpha-trypsin inhibitor heavy chain 1	-0.83	0.03	Inter-alpha-trypsin inhibitor heavy chain 1
DTNA	Dystrobrevin. alpha	-0.78	0.047	
CCDC87	Coiled-coil domain containing 87	-0.76	0.04	Microtubule cytoskeleton organization
INPP5B	Type II inositol 1.4.5-trisphosphate 5-phosphatase	-0.75	0.005	Inositol polyphosphate-5-phosphatase
SNORD32A	Small nucleolar RNA, C/D box 32A	-0.71	0.037	Small nucleolar RNA
ZNF250	Zinc finger protein 250	-0.68	0.03	Transcription factor
TPP	Tubulin polymerization promoting protein	-0.68	0.009	Tubulin polymerization promoting protein

*p<0.05 (paired t test, n=16)

Supplementary table 3. Gene ontology analysis of differentially expressed genes: CON vs OB (0 w)

GO-term	Pathway	Gene symbol	Number of genes/total number of genes in pathway	Fold enrichment	P-value
GO:0098609	Cell-cell adhesion	*CKAP5, PDLIM5, PI4KA, KTN1, EPS15L1, SH3GLB2, DHX29, EIF3E*	14 /271	2.3	0.0076
GO:0002479	Antigen presentation of exogenous antigen via MHC class I	*PSMB5, PSMF1, HLA-C, PSMA8, HLA-G, HLA-F*	6 / 63	4.3	0.0128
GO:0035338	Long-chain fatty-acyl-CoA biosynthetic process	*ACSBG2, ACOT13, PPT1, SCD5, ACSL3*	5 / 42	5.4	0.0137
GO:0043252	Sodium-independent organic anion transport	*SLCO4C1, SLC22A24, SLC22A8, SLCO2B1*	4 / 23	7.8	0.0137
GO:0016236	Macroautophagy	*PRKAG3, DYNLL2, LAMTOR2, ATG4B, PRKAB2, RRAGC*	6 / 76	3.6	0.0137
GO:0006633	Fatty acid biosynthetic process	*PRKAG3, CH25H, PRKAB2, SCD5, ACSL3*	5 / 52	4.3	0.0279
GO:0015031	Protein transport	*SDAD1, RAB3B, VPS52, KTN1, PPT1, AP4M1, STXBP5L, BBIP1, TMED5, CCT6B, PLEKHA8, ATG4B, IST1, AAGAB, RAMP1, HAP1*	16 / 395	1.8	0.0308
GO:0072593	Reactive oxygen species metabolic process	*NOX4, AOX1, BNIP3, LRRK2*	4 / 35	5.1	0.0419

Gene names given in italics.

Supplementary table 4. Gene ontology analysis of differentially expressed genes: OB (0 w) vs. (2 w)

**GO-term**	**Pathway**	**Gene symbol**	**Number of genes/total number of genes in pathway**	**Fold enrichment**	**P-value**
GO:0016310	Phosphorylation	*CIITA, N4BP2, ALDH18A1, PANK3, PAK2, NOL9, PI4KA, FN3KRP, PMS2P1, SGMS1, NMRK2*	11 / 100	3.3	0.0017
GO:0006629	Lipid metabolic process	*PLCL2, GDE1, HNF4A, PTGDS, TPP1, PITPNA, CPNE7, JAZF1, PLPP3, GDPD1, UGT2B7, SLC27A4*	12 /157	2.3	0.0153
GO:0019886	Antigen presentation of exogenous antigen via MHC class II	*DYNC1I1, HLA-DQB1, RAB7A, AP1B1, SEC13, TRAF6, SEC24D, CTSF*	8 / 92	2.6	0.0326
GO:0070534	Protein K63-linked ubiquitination	*TRIM56, WWP2, ITCH, TRAF6, UBE2S*	5 / 37	4.1	0.0329
GO:0010629	Negative regulation of gene expression	*SLC35C2, XDH, ZNF281, CDC42, WWP2, FLOT2, CRH, NDFIP2, FLCN, KLF4*	10 / 137	2.2	0.0382
GO:0030819	Positive regulation of cAMP biosynthetic process	*ADORA2A, GPR161, RXFP2, CRH, MC4R*	5 / 40	3.8	0.0422
GO:0030163	Protein catabolic process	*LONP2, PSMC3, TPP1, PPT1, NAPSA*	5 /41	3.7	0.0456

Gene names given in italics.

Supplementary table 5. Gene ontology analysis of transiently differentially expressed genes: OB (0 w) vs. (2 w)

GO-term	Pathway	Gene symbol	Number of genes/total number of genes in pathway	Fold enrichment	P-value
GO:0007188	Adenylate cyclase-modulating G-protein coupled receptor signaling pathway	*RXFP1, ADORA2A, RXFP2, ADRA1B, MC4R*	5 / 37	5.1	0.020
GO:0016310	Phosphorylation	*CIITA, N4BP2, ALDH18A1, PAK2, PI4KA, FN3KRP, PMS2P1, NMRK2*	8 / 100	3	0.020
GO:0006629	Lipid metabolic process	*GDE1, PTGDS, TPP1, PITPNA, CPNE7, JAZF1, PLPP3, GDPD1, UGT2B7, MTTP*	10 / 157	2.4	0.030
GO:0006954	Inflammatory response	*CIITA, IL23R, IL18RAP, OLR1, ADORA2A, TIRAP, BDKRB1, MAPKAPK2, APOL3, SLC11A1, TNFRSF11B, FOLR2, CXCL13, IL1RAP, CRH, BCL6, ITCH, THEMIS2*	18 / 379	1.8	0.030
GO:0045943	Positive regulation of transcription from RNA polymerase I promoter	*UBTF, ERBB2, PHF8*	3 / 10	11.2	0.030
GO:0000122	Negative regulation of transcription from RNA polymerase II promoter	*E2F6, TAF9B, MED25, CNOT2, FLCN, VDR, WWP2, BCL6, E4F1, CIITA, ZNF281, SCRT2, FZD8, TAF3, KLF12, ZFP57, ZHX2, SKI, NR0B1, NKX6-1, DKK1, HBZ, JAZF1, ZNF431, NFE2L3, TBX18, SMARCA2, RERE, KLF4*	29 / 720	1.5	0.030
GO:0010629	Negative regulation of gene expression	*XDH, ZNF281, CD3E, WWP2, FLOT2, CRH, NDFIP2, FLCN, KLF4 449*	9 / 137	2.5	0.030
GO:0051973	Positive regulation of telomerase activity	*NVL, PKIB, KLF4, NEK7*	4/ 29	5.2	0.040

Gene names given in italics.

Supplementary table 6. Gene ontology analysis of differentially expressed genes: OB (0w) vs. (12w).

**GO-term**	**Pathway**	**Gene symbol**	**Number of genes/total number of genes in pathway**	**Fold enrichment**	**P-value**
GO:0016032	Viral process	*KAT2A, CRTC2, SLC25A4, UNG, SYNCRIP, SNW1, SNAPIN, HLA-C, HLA-B, RCC1, GTF2B, VDAC1, WDR48, RAB6A*	14 / 299	2	0.024
GO:0042593	Glucose homeostasis	*ASPSCR1, CRTC2, PYGL, BHLHA15, SOX4, SIRT6, USF1*	7 / 101	3	0.034
GO:0006814	Sodium ion transport	*SLC12A1, CATSPER3, SLC17A4, SLC9B2, SLC5A6, SCNN1G*	6 / 81	3.1	0.041
GO:0010923	Negative regulation of phosphatase activity	*ZCCHC9, LMTK3, NIFK, DLG2, FARP1*	5 / 51	4.1	0.031
GO:0035902	Response to immobilization stress	*UCN3, LRP11, TPH1, NR0B1*	4 / 21	11.2	0.010
GO:0007259	JAK-STAT cascade	*NMI, STAT5B, JAK2, IFNAR1*	4 / 32	1.5	0.038

Gene names given in italics.

Supplementary table 7. Gene ontology analysis of differentially expressed genes: OB (0w) vs. (24w).

GO-term	Pathway	Gene symbol	Number of genes/total number of genes in pathway	Fold enrichment	P-value
GO:0043547	Positive regulation of GTPase activity	*DENND6A, BCR, PDGFB, LAMTOR2, PDGFA, FFAR1, DENND2C, CDKL5, STXBP5L, RIC8A, CCL11, ANKRD27, ARHGAP6, TBC1D17, CCL3L3, DLG4, SPTBN1, JAK2, LRRK2, EIF2B3, CDC42EP3, EPS8L1*	22 / 565	2	0.003
GO:0046777	Protein autophosphorylation	*STK33, BCR, CDK12, JAK2, LRRK2, ATP13A2, EPHB4, CDKL5, CAMKK2*	9 / 172	2.7	0.02
GO:0034220	Ion transmembrane transport	*ANXA6, SGK1, ATP2C2, SLC12A1, DLG4, SLC24A5, SLC9B2, CALHM3, ATP13A2*	9 / 210	2.2	0.047
GO:0070588	Calcium ion transmembrane transport	*TRPC1, ATP2C2, TRPM5, SLC24A3, SLC24A5, CACNB2, MCOLN1, PKD2L2*	8 /119	3.5	0.007
GO:0048008	Platelet-derived growth factor receptor signaling pathway	*BCR, PDGFB, PDGFA, JAK2*	4 / 29	7.2	0.01

Gene names given in italics.

Supplementary table 8. Gene ontology analysis of differentially expressed genes: OB (0 w) vs. (52 w)

GO-term	Pathway	Gene symbol	Number of genes/total number of genes in pathway	Fold enrichment	P-value
GO:0060333	Interferon-gamma-mediated signaling pathway	*NMI. CD44. TRIM31. HLA-C. HLA-B. HLA-E. MID1. IFNGR2*	8 / 71	3.4	0.0091
GO:0008380	RNA splicing	*PPP4R2. RBM20. SYNCRIP. SF3B4. IWS1. DDX47. JMJD6. RBM8A. SNRNP40. PTBP3. THOC5. HNRNPC. SREK1IP1*	13 / 166	2.4	0.0094
GO:0034765	Ion transmembrane transport	*K1. GRIK1. ATP10A. SLC24A5. CLCNKB. ATP2B2. ATP2C2. ATP2A2. WWP1. CALM3. ANO5. NALCN. CASQ1. HTR3B*	14 / 210	2.0	0.024
GO:0035725	Sodium ion transmembrane transport	*TRPM5. SLC4A11. SLC24A3. SLC17A1. SCN2A. SLC24A5. NALCN*	7 / 40	2.9	0.034
GO:0070588	Calcium ion transmembrane transport	*ATP2B2. ATP2C2. TRPM5. ATP2A2. PSEN1. SLC24A3. SLC24A5. CACNG4. NALCN*	9 / 51	2.3	0.045

Gene names given in italics.

Supplementary table 9. Gene ontology analysis: differentially expressed genes affected by DNA methylation (52 w)

GO-term	Pathway	Gene symbol	Number of genes/total number of genes in pathway	Fold enrichment	P-value
GO:0051149	Positive regulation of muscle cell differentiation	*MYOD1, SPAG9, SETD3, CTNNA2*	4 / 24	8.6	0.0108
GO:0007040	Lysosome organization	*VPS18, GNPTAB, MYO7A, LRRK2*	4 / 36	5.7	0.0320
GO:0030819	Positive regulation of cAMP biosynthetic process	*SCT, ADCY7, GPR161, RAMP1*	4 / 40	5.2	0.0419
GO:0006468	Protein phosphorylation	*PRKAG3, MYOD1, SGK1, STK17B, PRKD1, CCNE1, IKBKE, ICK,VRK1, MAP3K8, MERTK, LRRK2,CDK15, CAMK1D, AATK*	16 / 456	1.8	0.033
GO:0035023	Regulation of Rho protein signal transduction	*MCF2L2, ARHGEF2, FARP1, ARHGEF10, EPS8L1, NET1*	6 / 81	3.8	0.0204
GO:0006979	Response to oxidative stress	*PSMB5, PTGS2, PSEN1, LIAS, CYGB, LRRK2, OXR1*	7 / 110	3.3	0.0201
GO:0006979	Covalent chromatin modification	*BAG6, MBTD1, SMARCD3, SMARCC1, DNAJC2, NCOR1, ARID2*	7 / 113	3.2	0.0226
GO:0070588	Calcium ion transmembrane transport	*ATP2B2, ATP2C2, TRPM5, ATP2A2, PSEN1, CACNG4, NALCN*	7 / 119	3.0	0.0282
GO:0008380	RNA splicing	*DDX47, PPP4R2, JMJD6, RBM20, PTBP3, SNRNP40, HNRNPC, IWS1*	8 / 166	2.5	0.0429
GO:0034220	Ion transmembrane transport	*ATP2B2, SGK1, ATP2C2, ATP2A2, WWp1, ATP10A, CALM3, ANO5, NALCN, HTR3B*	10 / 210	2.5	0.0214
GO:0008152	Metabolic process	*ACSM3, ECH1, SUCLG2, HOGA1, ECHDC2, FLAD1, SCP2, ENOSF1*	8 / 168	2.5	0.0453
GO:0035556	Intracellular signal transduction	*PRKAG3, CAPS, ARHGEF2, SGK1, ADCY7, STK17B, DGKH, CISH, PRKD1, TNS3, ICK, PSEN1, RASSF1, PLCD3, ASB3, LRRK2, NET1*	17 / 403	2.2	0.0054

Gene names given in italics.

Supplementary table 10: Gene ontology analysis of correlated genes to fasting glucose levels

**GO-term**	**Pathway**	**Gene symbol**	**Number of genes/total number of genes in pathway**	**Fold enrichment**	**P-value**
GO:0043388	positive regulation of DNA binding	*HDAC5, TNF, SKI, MAP3K13*	4 / 70	8.9	0.01
GO:0009057	macromolecule catabolic process	*SUMO3, DDB1, MAGOH, CBL, PPP2R5C, RING1, ZHX2, BIRC6, ERN2, CHI3L2, YME1L1, USP15*	12 / 781	2.4	0.01
GO:0051099	positive regulation of binding	*HDAC5, TNF, SKI, MAP3K13*	4 / 78	8	0.013
GO:0006887	exocytosis	*STEAP2, SLC17A9, TNP2, EXOC6B*	4 / 115	5.4	0.037
GO:0032940	secretion by cell	*STEAP2, SLC17A9, TNP2, APBA1, EXOC6B*	5 / 207	3.8	0.043

Gene names given in italics.

Suppl. Figure 1. **Time course of changes in muscle lipid intermediates.**

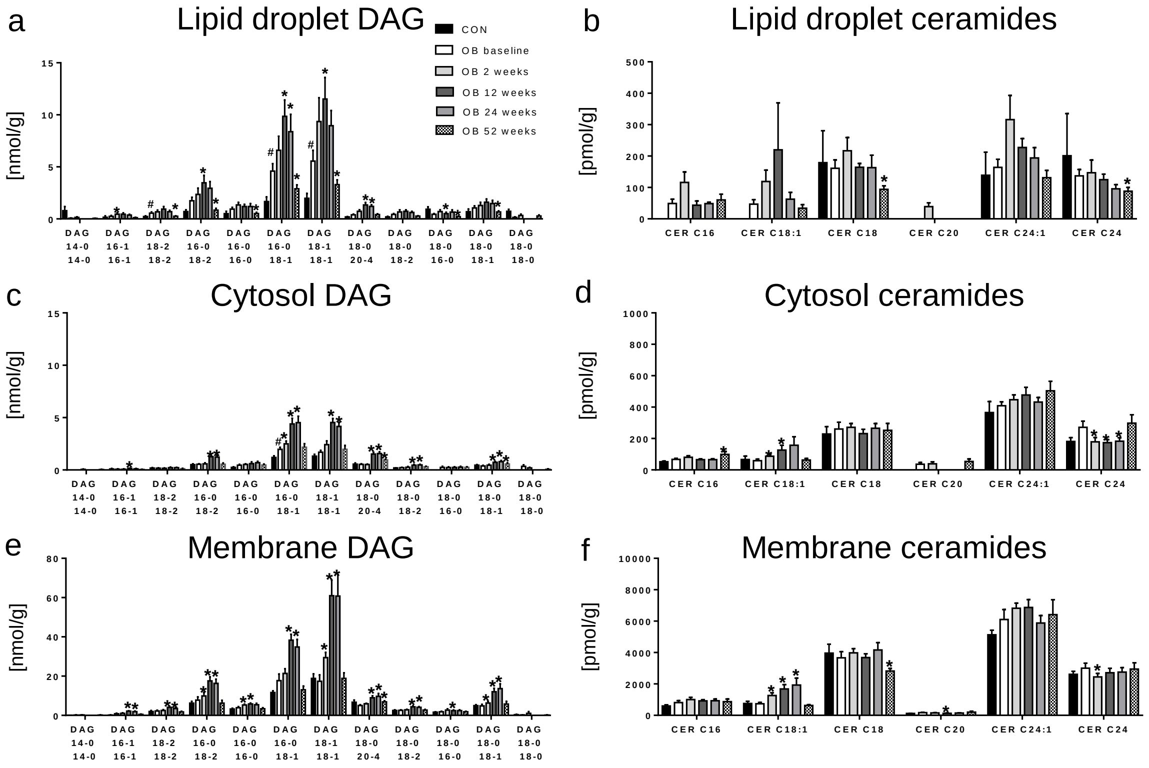

Diacylglycerol (DAG) concentrations in lipid droplet (a), cytosolic (c) and membrane (e) fractions. Ceramide concentrations in lipid droplet (b), cytosolic (d) and membrane (f) fractions. Mean±SEM, *p<0.05 vs OB at baseline using covariance pattern model for repeated measures analysis, #p<0.05 vs CON using unpaired 2-tailed t-test. CON-nonobese humans, OB-obese humans.

Suppl. Figure 2. **Time course of changes in muscle mitochondrial electron transport chain complexes**.

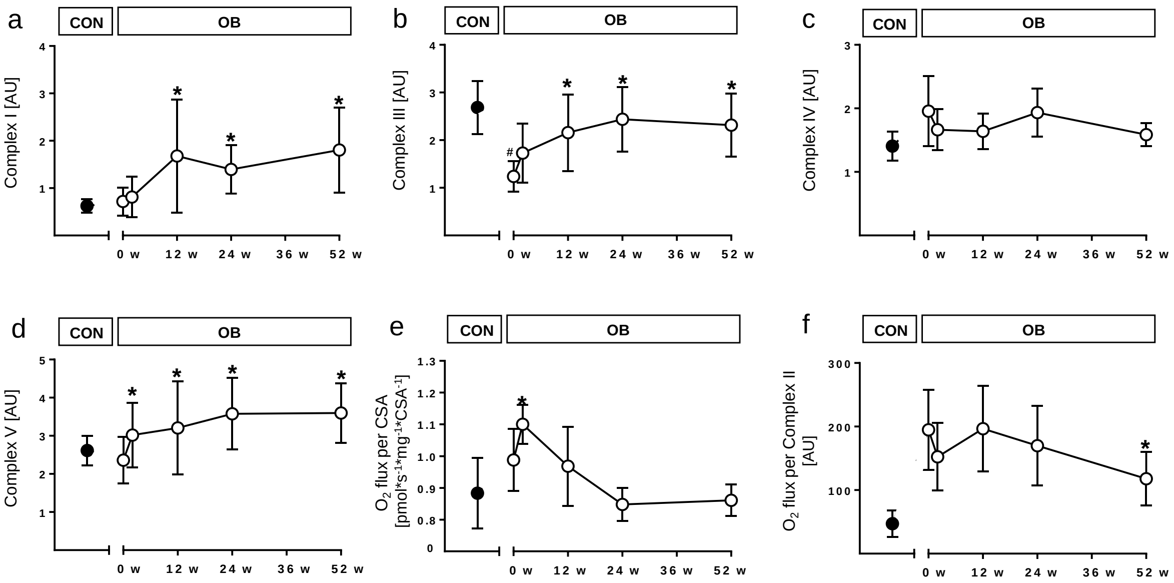

Complex I (NADH:ubiquinone oxidoreductase subunit B8) (a), complex III (ubiquinol-cytochrome C reductase core protein 2) (b), complex IV (cytochrome c oxidase subunit IV) (c), complex V (ATP synthase F1 subunit alpha) (d), maximum mitochondrial respiration per citrate synthase activity (CSA) (e) and maximum mitochondrial respiration per complex II content (f) in obese (empty circles) and in nonobese humans at baseline (black circles). Mean±SEM, *p<0.05 vs OB at baseline using covariance pattern model for repeated measures analysis, #p<0.05 vs CON using unpaired 2-tailed t-test. CON-nonobese humans, OB-obese humans.

Suppl. Figure 3. Time course of changes in muscle protein content of mitochondrial fusion and fission markers.

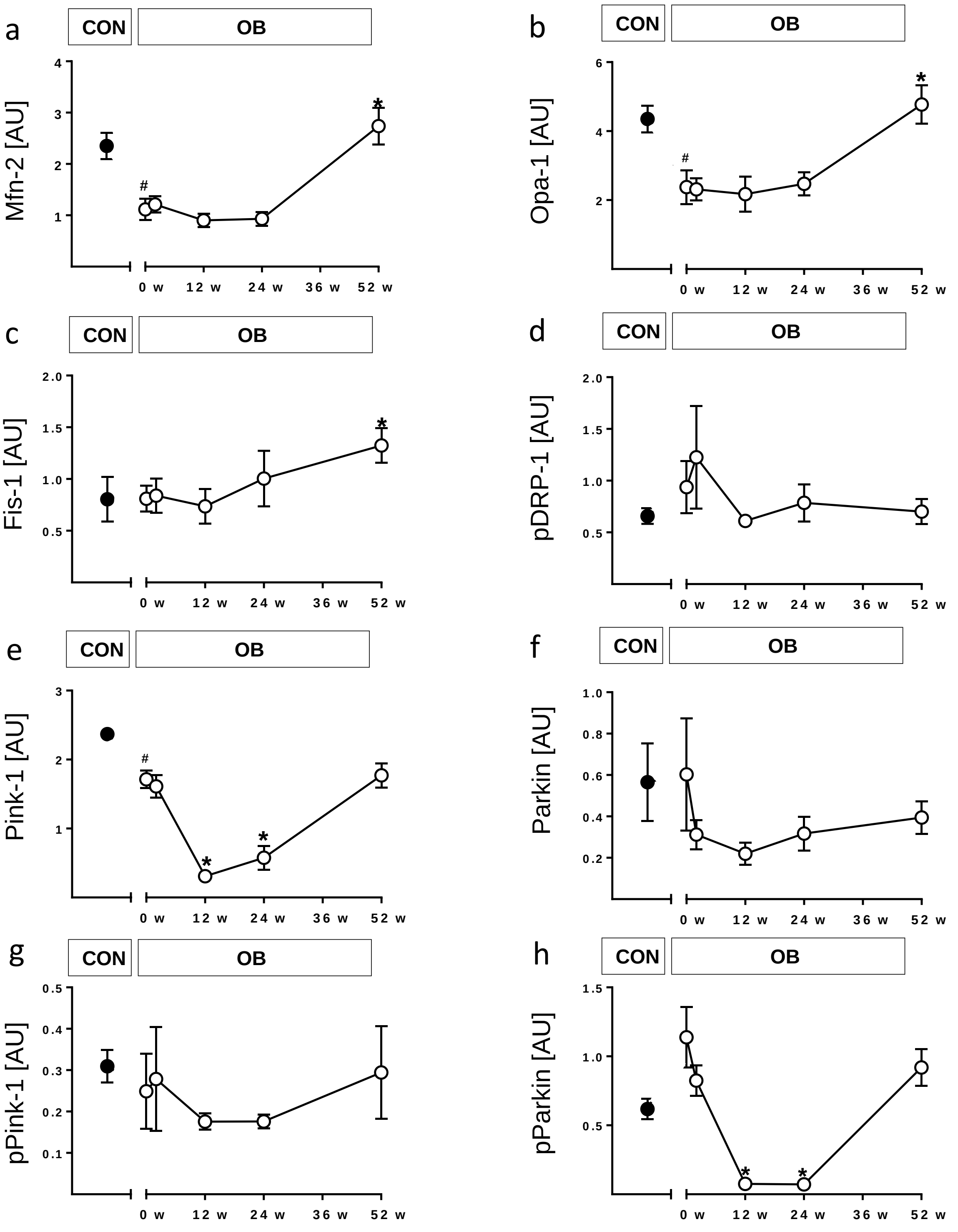

Mitofusin-2 (Mfn-2) (a), optic atrophy 1 protein (Opa-1) (b), mitochondrial fission 1 protein (Fis-1) (c), phospho-Ser^{616}-DRP1 (d), Pink-1 (e), Parkin (f), phospho-Thr^{257}-Pink1 (g) and phospho-Ser^{65}-Parkin (h) in obese (empty circles) and in nonobese humans at baseline (black circles).

Mean±SEM, *p<0.05 vs OB at baseline, using covariance pattern model for repeated measures analysis, #p<0.05 vs CON using unpaired 2-tailed t-test. CON-nonobese humans, OB-obese humans.

Suppl. Figure 4 Mitochondrial volume density from electron microscopy analysis.

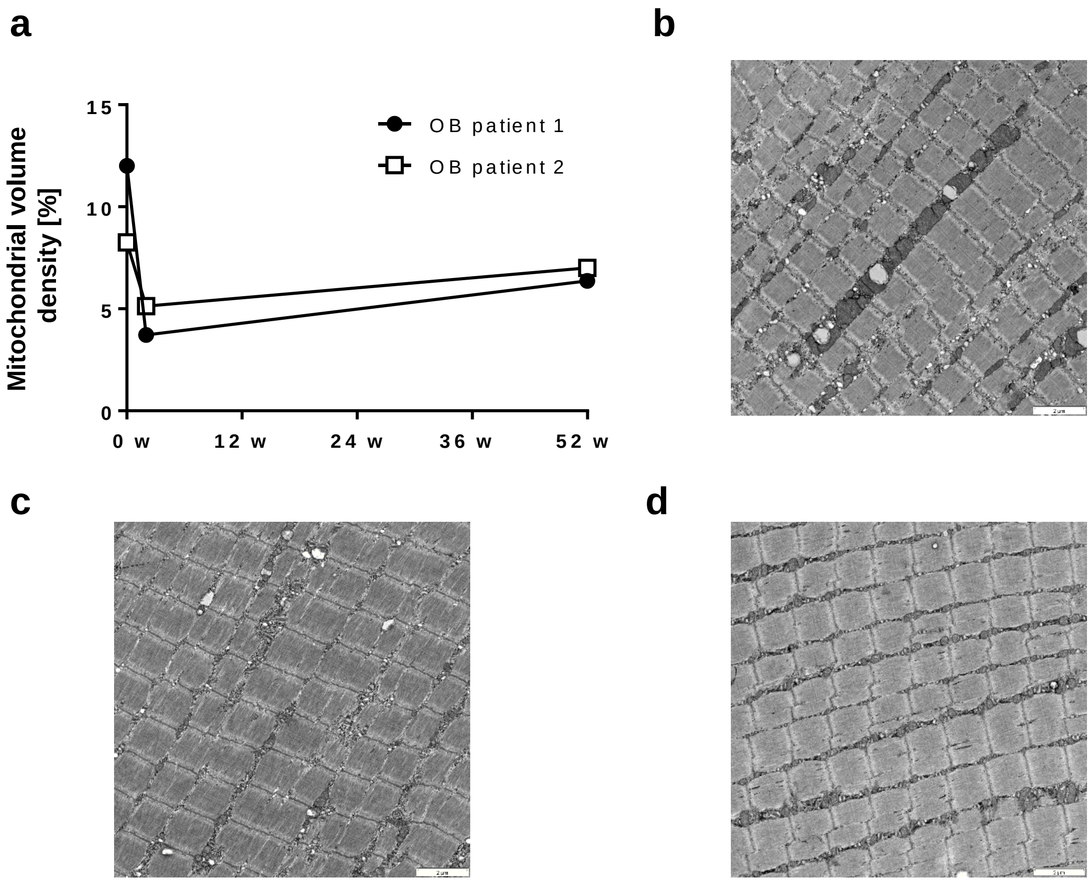

Data from two obese patients (a) and representative micrographs from baseline (b), 2 weeks (c) and 52 weeks (d) after metabolic surgery. Empty squares – obese patient Nr.1, black circles – obese patient Nr. 2. OB-obese humans.

Suppl. Figure 5. Gene ontology analysis of transiently altered transcripts.

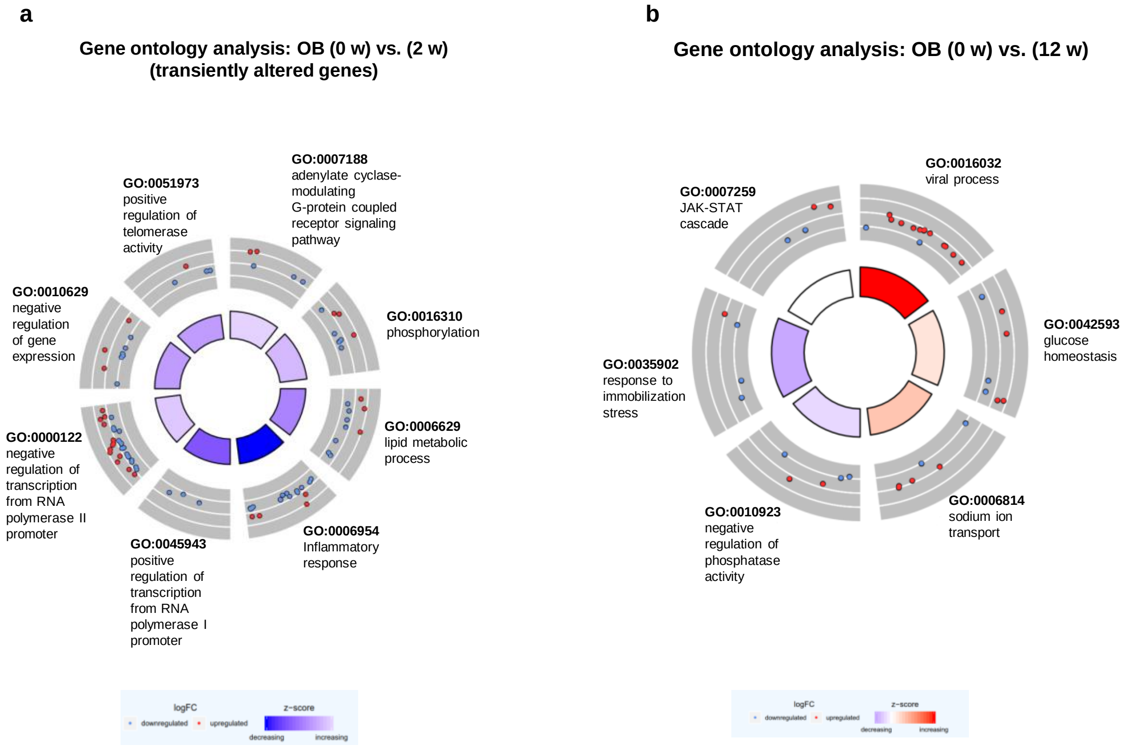

Transcripts at 2 weeks after metabolic surgery (a) and at 52 weeks (b). The inner circle depicts the main processes to be increased (blue) or decreased (red) in obese participants transiently at 2 weeks (a) and 52 weeks (b). The outer circle shows scaled scatter plots for affected genes and their regulation within the most-enriched biological pathway.

Suppl. Figure 6. **Alterations in skeletal muscle DNA methylation at 2 and 52 weeks after metabolic surgery.**

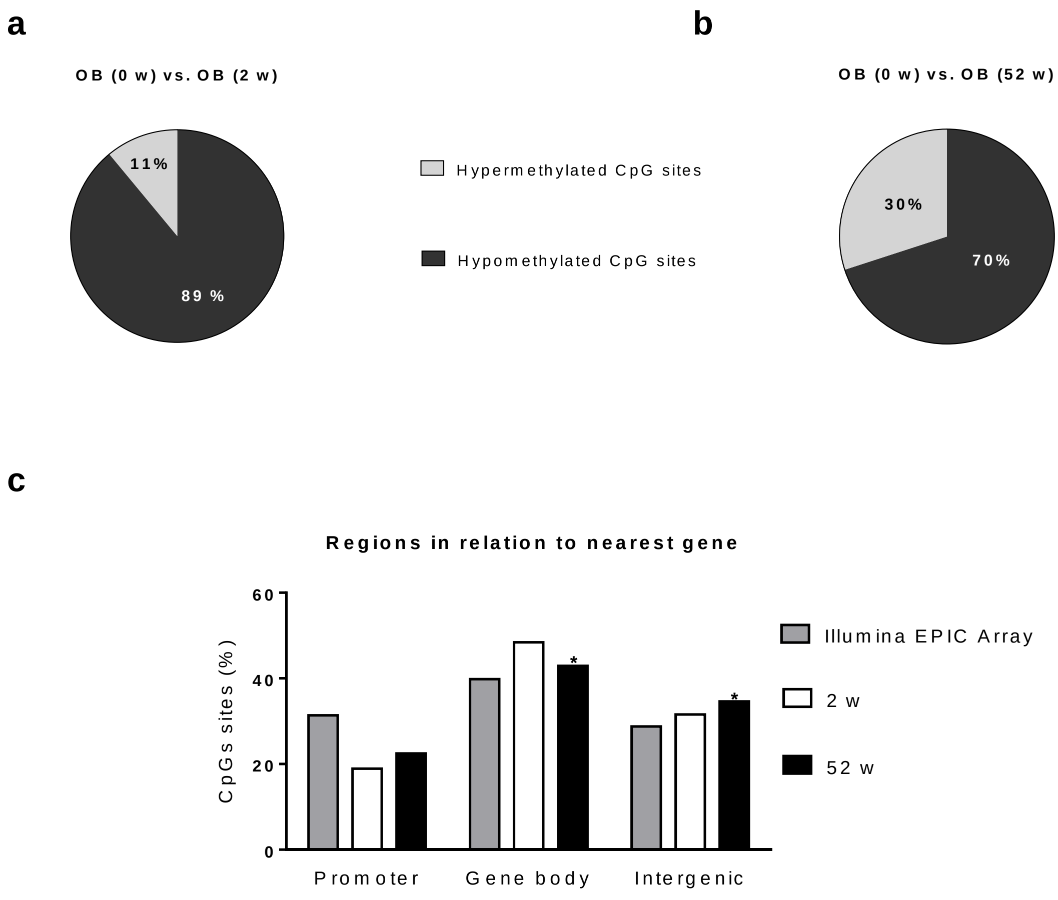

The pie charts show the percentage of individual CpG sites exhibiting significantly increased (bright grey) and decreased (dark grey) methylation at baseline vs. 2 weeks (a) and vs. 52 weeks (b). Localization of differentially methylated CpGs in relation to the nearest gene region (c). Chi-square analysis was performed to test over- or under-representation of sequence features among the differentially methylated CpGs vs. all analyzed CpGs.*p<0.01 (paired t test, n=16), *p<0.05

Suppl. Figure 7. Venn diagram summarizing the strategy for the identification of novel candidate genes involved in the improvement of the insulin sensitivity after bariatric surgery.

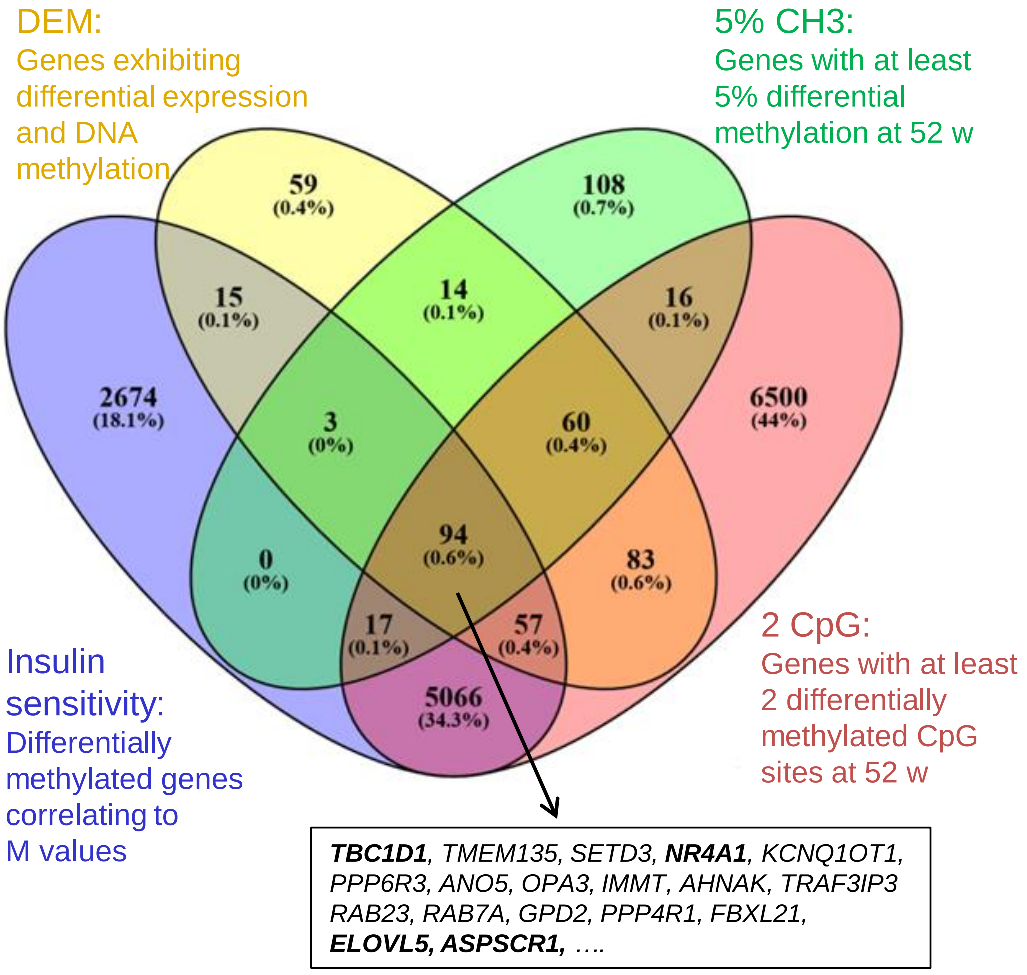

For insulin sensitivity Pearson correlation was performed with all individual DNA methylation at 0, 2, and 52 w.

Suppl. Figure 8. Study design.

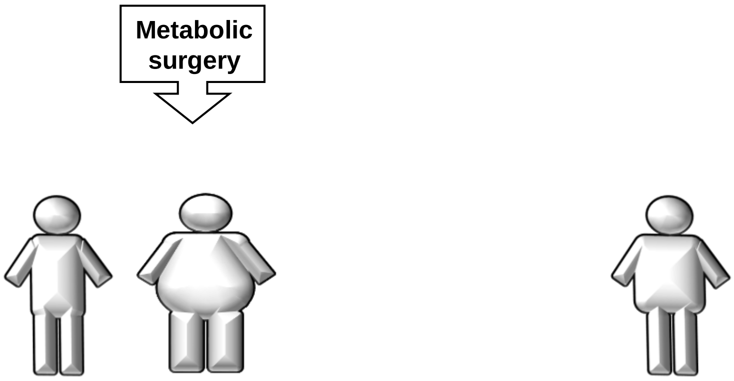

	0 w	2 w	12 w	24 w	52 w
Anthropometry	✔	✔	✔	✔	✔
Lab analyses	✔	✔	✔	✔	✔
Hyperinsulinemic-euglycemic clamp	✔	✔	✔	✔	✔
Muscle mitochondrial densitiy and function	✔	✔	✔	✔	✔
Muscle lipidomics	✔	✔	✔	✔	✔
Muscle genome-wide transcriptomics	✔	✔	✔	✔	✔
Muscle genome-wide methylomics	✔	✔			✔

Obese participants (OB) were intensively characterized at 0 (baseline), 2, 12, 24 and 52 weeks after metabolic surgery. Nonobese humans (CON) were studied at baseline.

Suppl. Figure 9. Time course of mRNA expression of indicated genes.

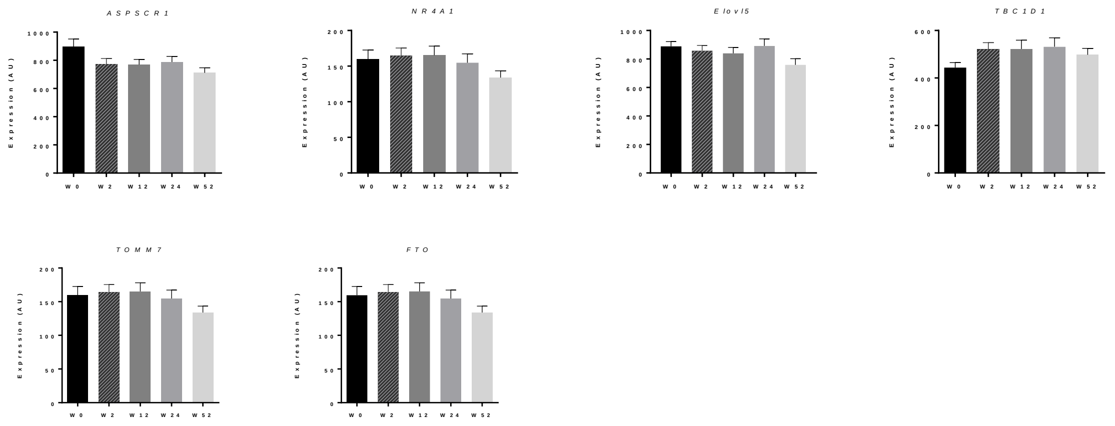

Suppl. Figure 10. Correlation of DNA methylation level of *ELOVL5* and *ASPSCR1* with M-values.

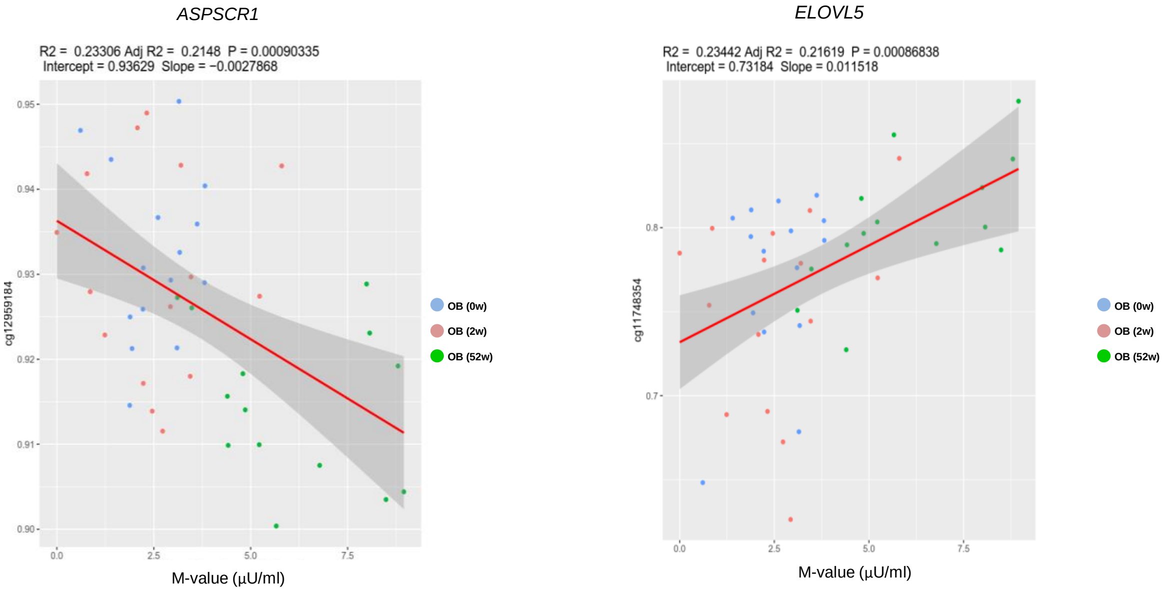

Suppl. Fig. 11. Representative blots for proteins from Western blot analysis

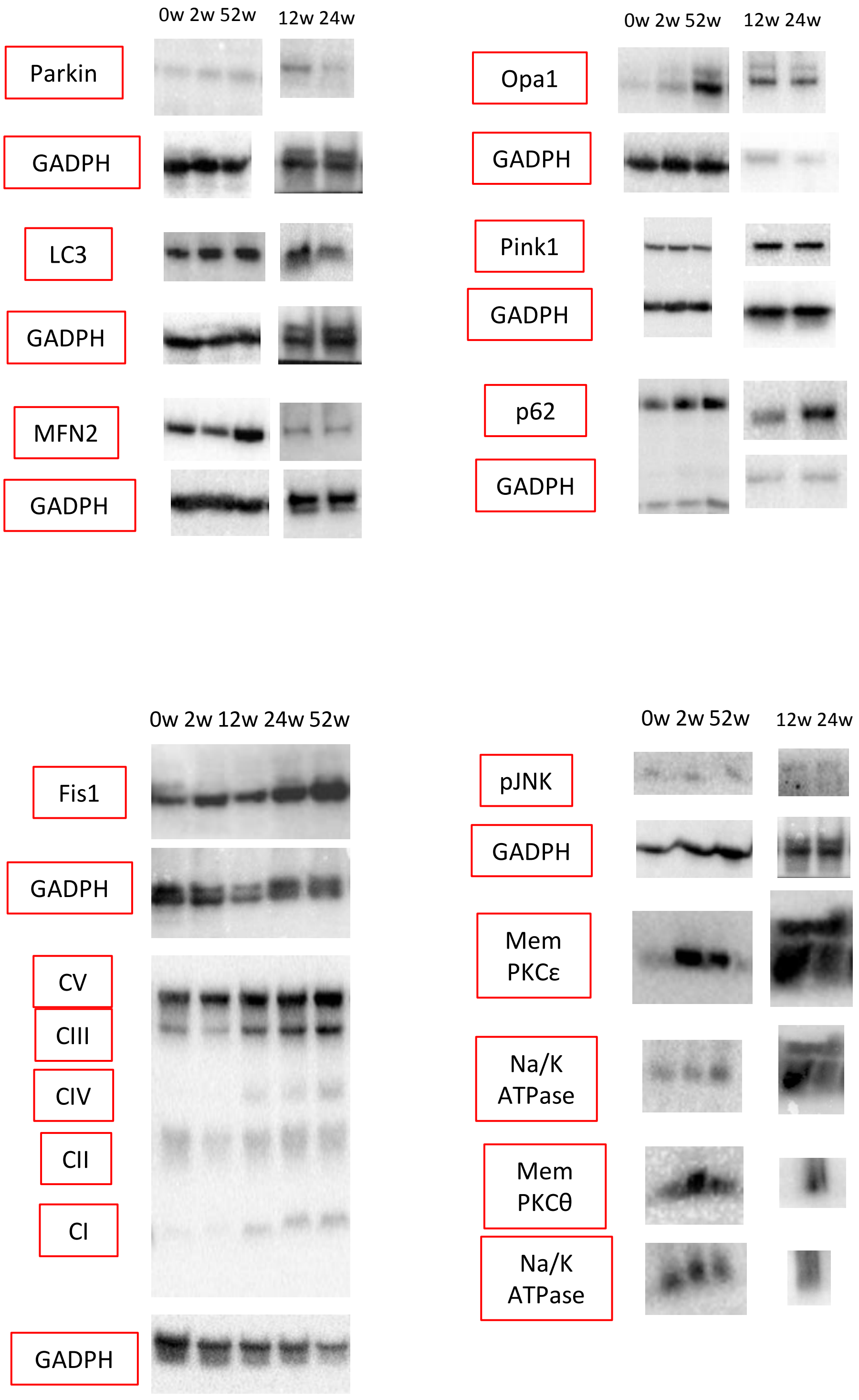

Supplement: Supplementary file 1 — Supplementary information [file 41467_2022_29350_MOESM1_ESM.pdf]
